# Sequential splicing of a group II twintron in the marine cyanobacterium *Trichodesmium*

**DOI:** 10.1038/srep16829

**Published:** 2015-11-18

**Authors:** Ulrike Pfreundt, Wolfgang R. Hess

**Affiliations:** 1 University of Freiburg, Faculty of Biology, Schänzlestr. 1, D-79104 Freiburg, Germany

## Abstract

The marine cyanobacterium *Trichodesmium* is unusual in its genomic architecture as 40% of the genome is occupied by non-coding DNA. Although the majority of it is transcribed into RNA, it is not well understood why such a large non-coding genome fraction is maintained. Mobile genetic elements can contribute to genome expansion. Many bacteria harbor introns whereas twintrons, introns-in-introns, are rare and not known to interrupt protein-coding genes in bacteria. Here we show the sequential *in vivo* splicing of a 5400 nt long group II twintron interrupting a highly conserved gene that is associated with RNase HI in some cyanobacteria, but free-standing in others, including *Trichodesmium erythraeum*. We show that twintron splicing results in a putatively functional mRNA. The full genetic arrangement was found conserved in two geospatially distinct metagenomic datasets supporting its functional relevance. We further show that splicing of the inner intron yields the free intron as a true circle. This reaction requires the spliced exon reopening (SER) reaction to provide a free 5′ exon. The fact that *Trichodesmium* harbors a functional twintron fits in well with the high intron load of these genomes, and suggests peculiarities in its genetic machinery permitting such arrangements.

The diazotrophic *Trichodesmium* is a tropical marine cyanobacterium of global importance^1^. *Trichodesmium* is unusual in its genomic architecture, characterized by the presence of a large non-coding fraction, encompassing about 40% of the total genome length, compared to the cyanobacterial average of only 15%^2^,^3^. Natural populations of *Trichodesmium* possess a similarly high non-coding genome share^4^, but why and how it is maintained is unknown. Interestingly, in laboratory studies about 80% of the non-coding fraction was found to be transcribed into non-coding RNAs^4^. Part of this non-coding genome fraction is occupied by introns^5^,^6^,^7^. In the model strain *Trichodesmium erythraeum* IMS101 (*T. erythraeum*), several genes are interrupted by one or multiple introns, primarily group II introns. Group II introns constitute the largest class of prokaryotic introns. They are frequently self-splicing, mobile ribozymes with conserved structural motifs and often possess an internal open reading frame (ORF) for an intron encoded protein (IEP). These IEPs may aid splicing through a maturase domain^8^,^9^ and confer mobility due to their reverse transcriptase and sequence-specific DNA endonuclease activity^10^. Frequently, these genes appear truncated, missing the endonuclease domain or encoding only the maturase domain. Due to their identical splicing chemistry, group II introns have been hypothesized to be the evolutionary ancestors of nuclear spliceosomal introns and the spliceosomal snRNAs^11^,^12^ and, in fact, to have contributed significantly to the evolution of the eukaryotic nucleus^13^.

*Trichodesmium* harbors a large number of introns within protein-coding genes. Of particular interest are ORF-less group II introns, because their splicing may depend on *trans*-encoded but co-expressed maturases^5^. From the 17 group II introns in *T. erythraeum* documented to splice *in vivo*, ten lack an intron-located ORF^3^. Thus, these must either splice autocatalytically or depend on *trans-*encoded splicing factors. The latter has been shown through heterologous expression in *E. coli*^5^. Both the dependence on *trans-*encoded splicing factors and the location within protein-coding genes is rather typical for organellar group II and eukaryotic spliceosomal introns, but less usual for bacterial group II introns that are mostly located in intergenic regions^11^,^14^,^15^,^16^. Along the evolutionary trajectory from bacterial via organellar to spliceosomal introns, the substitution of IEPs by *trans*-encoded protein factors facilitating splicing is an essential step^11^,^17^. Therefore, it is interesting to study the introns in *T. erythraeum* to gain further insight into processes fundamental to RNA biology and into their possible connection to the excessive non-coding fraction in this organism.

A twintron is a rare genetic arrangement in which one intron is nested within another intron. Adding to the list of unusual genome features of *Trichodesmium*, this genome contains a group II twintron which is also the only bacterial twintron known to date to be located within a protein-coding gene. The two introns comprising the twintron are described in the Calgary Group II intron database including splice site and structure prediction^15^. Here we document the transcription and sequential splicing of this twintron *in vivo*. The entire genetic element is more than 5,400 nt long and consists of two distantly related group II introns, each containing a different IEP ORF. We found the host gene, which codes for a highly conserved cyanobacterial hypothetical protein, to be mis-annotated as RNase H, but instead to constitute a separate protein domain that is fused to RNase HI in several cyanobacteria. Splicing of this twintron produces a putatively functional mRNA.

To our knowledge this is the first evidence for sequential splicing of a twintron in a bacterial protein-coding gene *in vivo*. We define the gene product of the mature mRNA as a novel protein domain that appears itself connected to RNA metabolism.

## Results

### Sequential splicing of a twintron in an RNase HI-associated gene

The two introns comprising the twintron are described in the Group II intron database^15^ as T.e.I7 and T.e.I8. The two introns are linked to the gene *Tery_4732*, but an easily recognizable 3′ exon was lacking. We compared the host gene product (*Tery_4732*) against the NCBI non-redundant protein database and constructed multiple alignments for the identified homologues. The host gene encodes a protein that is misannotated as RNase HI in some genomes, and as hypothetical protein in others (see analysis further down). Strikingly, nine otherwise highly conserved amino acids, ILWQLERVD, were missing at the C terminus. A sequence stretch 5,108 nt downstream of *Tery_4732* could be translated to LLWQLERVD. We hypothesized that this sequence stretch could be part of the 3′ exon, which would furthermore be consistent with the computationally predicted splice sites in the database for bacterial group II introns (http://webapps2.ucalgary.ca/~groupii, FEB 06, 2015)^15^.

**Figure 1:**
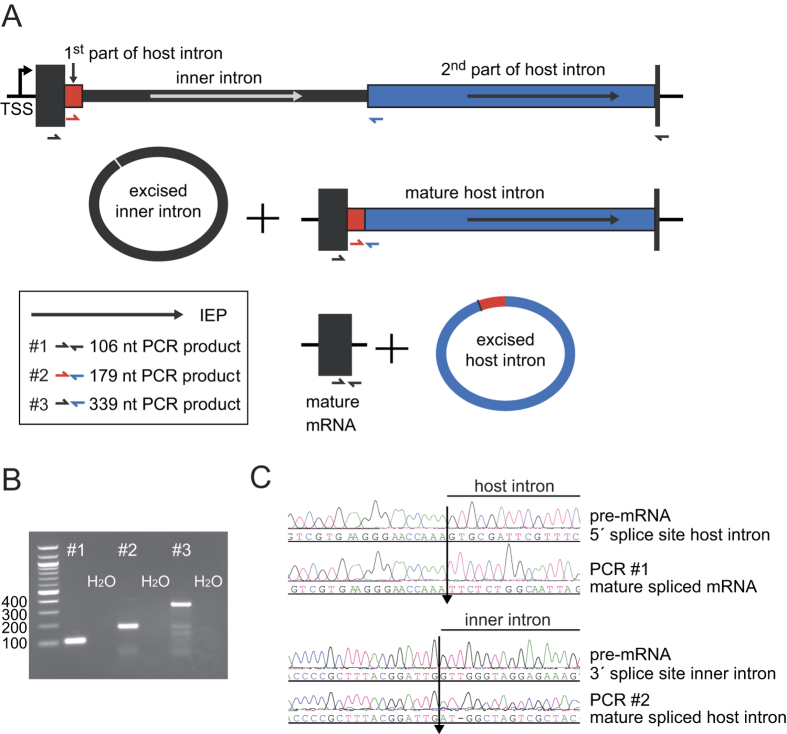
A twintron is present in the genome of *T. erythraeum* IMS101 and is actively spliced. (**A**) Schematic representation of the twintron and its proposed splicing steps. In the scheme from left to right: arrow =TSS of the pre-mRNA, dark box = interrupted ORF *Tery_4732*, red box = 1^st^ part of host intron, thin black box: inner intron interrupting domain 1 of the host intron, blue box = 2^nd^ part of host intron, dark short dark box = 3′ exon of *Tery_4732* mRNA. Arrows indicate the IEP ORFs *Tery_4730* and *Tery_4731* in the two introns (the IEPs share 30%/47% identical/similar residues with each other). The half-arrows aligned to the scheme indicate the primers used. The inset legend shows color-coded primer combinations used for the different amplicons. (**B**) #1 RT-PCR with primers in the 5′ (*Tery_4732*) and the 3′ mRNA-exon (black) yielded a product with the mature mRNA sequence (106 nt). #2 primers in the host intron (red and blue) yielded a product with the mature host intron sequence (179 nt, inner intron spliced out). #3 primers in *Tery_4732* and the 2^nd^ part of the host intron (black forward and blue reverse) verified the existence of an intermediate precursor without the inner intron (339 nt). H_2_O denotes control PCR without template cDNA. (**C**) Sequencing traces of amplicons #1 and #2, each compared to the respective unspliced sequence from the pre-mRNA. The arrows mark the identified splice sites. Note that the upper two sequences are in forward orientation, the lower two in reverse.

The genetic arrangement of the corrected, twintron-interrupted gene *Tery_4732* is depicted in Fig. 1A. In the following we will refer to the intron that is located within another intron as the inner intron. The interrupted intron will be called host intron, the twintron-disrupted gene the host gene. To confirm that splicing of both introns was functional *in vivo*, cDNA was prepared from total RNA using three specific reverse primers, one each in the putative 3′ exon, the 2^nd^ part of the putative host intron and the possible inner intron. This cDNA was amplified with six different primer combinations (see Methods and Fig. 1B) and sequenced. The sequencing traces of amplicons #1 and #2 compared to the respective non-spliced sequences provided evidence for the presence of the correctly spliced mature mRNA as well as the mature, continuous host intron after inner intron splicing (Fig. 1C). This analysis localized the 3′ exon-intron boundary between the 1^st^ (A) and 2^nd^ (T) nucleotide of the codon for the first missing amino acid, yielding an isoleucine codon (ATT) instead of leucine after splicing. We conclude that the sequence encoding (I)LWQLERVD (positions 7258469 to 7258498 on the reverse strand when including the stop codon) is indeed part of the 3′ exon, and after transcription and splicing it forms the 3′ end of the mature mRNA.

Because the transcriptional start site of *Tery_4732* was mapped to genomic position 7263905r^3^, the primary transcript is at least 5,436 nt long. In the Genbank file CP000393.1, the six last amino acids SAIRFC of *Tery_4732* are annotated incorrectly because the corresponding nt positions already belong to the twintron.

The amplicons presented in Fig. 1B verify the most important splicing steps as follows. The presence of the continuous host intron RNA (Fig. 1, primer pair #2) proved that the inner intron can splice independently. To test whether the inner intron spliced before or after splicing of the host intron, primer pair #3 was designed to detect the intermediate precursor where the inner intron has spliced, but the host intron has not (Fig. 1A, middle scheme). The corresponding 339 nt amplicon (Fig. 1B) can only be obtained in absence of the inner intron. This indicated that prior splicing of the inner intron is most probably necessary for correct splicing of the host intron. The mature mRNA (primer pair #1) can originate only from splicing of both the inner and host introns and thus is evidence for functional splicing of both. The genomic positions of the 5′ and 3′ splice sites for the host intron were determined to be after nucleotides 7263622r and 7258498r, respectively, and for the inner intron after 7263468r and 7261005r, respectively. We conclude that sequential splicing of the twintron was functional and that the host gene product after intron splicing is 89 amino acids long (from here referred to as: *Tery_4732*+).

### The twintron’s host gene encodes a highly conserved cyanobacterial protein

**Figure 2:**
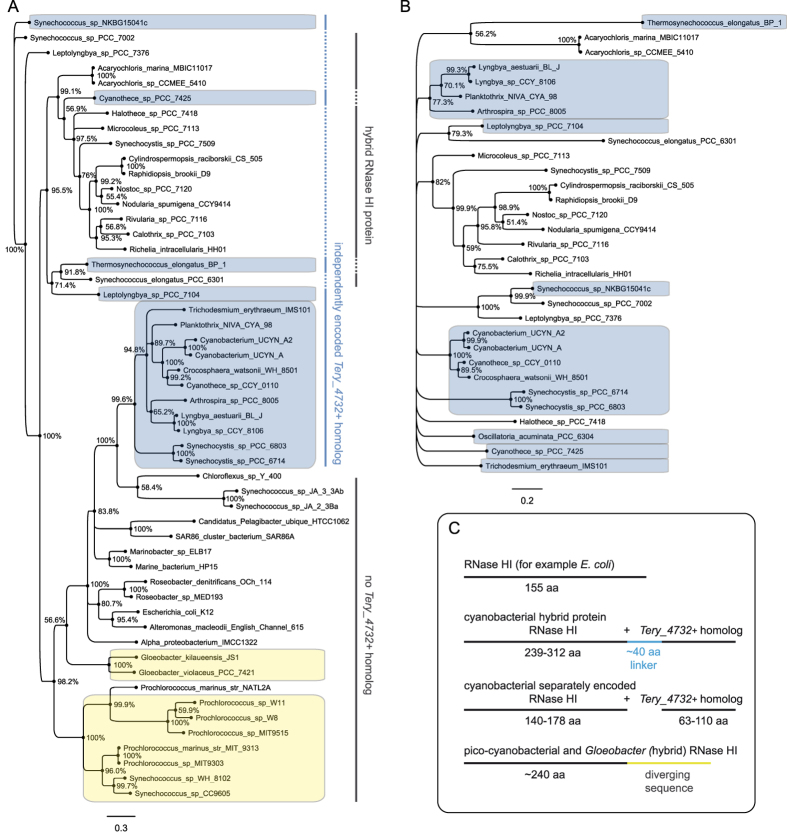
Phylogeny of homologs of the RNase HI gene and the twintron host gene. Separate Bayesian trees showing the phylogenetic relationships of solitary and hybrid RNase HI gene homologs (**A**) and *Tery_4732*+ homologs (**B**). Percentages at each node are posterior probabilities for the existence of that node, as inferred by Mr. Bayes^42^. Those species containing independently encoded RNase HI and *Tery_4732*+ homologs are in blue boxes. The yellow boxes mark species with an RNase HI carrying a 3′extension that is not homologous to *Tery_4732*+. (**C**) Schematic comparison of RNase HI genes and RNase HI hybrid genes in cyanobacteria, picocyanobacteria and other bacteria (represented by *E. coli)*. Cyanobacterial hybrid RNase HI proteins contain a non-conserved linker sequence of approximately 40 amino acids (blue) between the two domains representing RNase HI and the *Tery_4732*+ homolog, respectively.

We analyzed the host gene *Tery_4732*+ product (MNETTNELVENMPREQRVGQLRNLIETLHIADEVANKGYLITSSELADLMDINASAVTSRGDHWVWRNWVVSRVRREGNQILWQLERVD) by multiple reciprocal BLASTP searches and sequence alignments (Suppl. Alignment 1, 2, 3). This protein is conserved in cyanobacteria (>70% identical residues); however, in many species it is not encoded by an independent gene but is fused to the RNase HI gene, forming the C-terminal domain of an RNase HI hybrid protein (Fig. 2C). In these cases, the enzymatically active RNase HI domain is located in the N-terminal part of the hybrid protein. This arrangement is restricted to the phylum cyanobacteria as is the presence of proteins homologous to *Tery_4732*+. In contrast, RNase HI is present ubiquitously, consistent with its conserved function in removing the RNA primer during DNA replication^18^. In *T. erythraeum, Tery_1584* encodes RNase HI, located separately and >4.8 Mbp away from *Tery_4732*+ on the genome.

Interestingly, all genomes containing an independently encoded *Tery_4732*+ homolog have a separate RNase HI gene similar in length to non-cyanobacterial RNase HI (these non-hybrid RNases are called “standard RNase HI” from here) and lack the hybrid gene, just like in *T. erythraeum*. When these two separate proteins were aligned to full-length RNase HI hybrid proteins, a linker sequence of approximately 40 amino acids became evident in the hybrid proteins (Fig. 2C, Suppl. Alignment 1). To analyze whether standard RNase HI and the *Tery_4732*+ homologs share the same phylogeny, two Bayesian phylogenetic trees were built (Fig. 2A,B) from all separate genes plus selected hybrid genes (only the respective part of the hybrids was aligned). We did not find the standard RNase HI sequences to be generally phylogenetically distinct from those in the hybrid forms. However, there is a phylogenetically distinct group of cyanobacterial standard RNase HI that cluster together with non-cyanobacterial standard RNase HI (Fig. 2A, large blue box). However, four sequences of cyanobacterial standard RNase HI were placed outside of this cluster (smaller blue boxes), interspersed with the hybrid forms. Thus, the solitary RNase HI genes do not comprise a single phylogenetic group within cyanobacteria. On the other hand this might be true for the hybrid form, because no RNase HI sequences from hybrid genes were grouped together with the non-cyanobacterial and the bulk of cyanobacterial standard RNase HI (Fig. 2A, large lower cluster). The phylogeny of *Tery_4732*+ homologs (Fig. 2B) is much flatter than that of RNase HI and reflects their higher degree of conservation. Clustering is not very clear, thus no statement can be made about whether or not these genes follow the same phylogenetic pattern as RNase HI.

There are some notable exceptions. *Microcystis aeruginosa* NIES-843, *Cyanothece* spp. PCC 7822 and PCC 7424, and *Oscillatoria acuminata* contain a separate *Tery_4732*+ homolog, but their RNase HI genes appear phylogenetically unrelated to all other cyanobacterial RNase HI. Further, the genomes of the strains *Synechococcus* Yellowstone A-Prime and B-Prime (JA-3-3Ab, JA-2-3B’a) and *Prochlorococcus marinus* NATL2A contain only standard RNase HI and no homolog of *Tery_4732*+. Phylogenetic analysis revealed that the RNase HI genes from these genomes are not closely related, suggesting that the *Tery_4732*+ homolog can be non-essential and lost from the genome independently from RNase HI (Fig. 2A). Interestingly, genomes of other picocyanobacteria as well as of *Gloeobacter* species contain RNase HI genes with a phylogenetically distinct 3′ extension (Fig. 2C, Suppl. Alignment 1) and do not contain a *Tery_4732*+ homolog.

The function of the *Tery_4732*+ homologs, both as the C-terminal domain of cyanobacterial RNase HI hybrids and as a separate entity, and thus the function of the twintron′s host gene product, remain unknown for the present time. The fact that *Tery_4732*+ homologs exist in almost all other cyanobacteria (except picocyanobacteria) and in about half of all cases form a hybrid with RNase HI suggest their intimate link. Therefore it is likely that *Tery_4732*+ homologs are connected, directly or indirectly, to the RNA biology of cyanobacteria.

### The *Tery_4732* twintron is conserved in metagenomics datasets

To address the connection between the twintron and its *Tery_4732*+ host gene, we scanned publicly available metagenomics datasets for similar loci. Indeed, the full genetic arrangement (5′ UTR - mRNA exon - twintron - 3′ mRNA exon - 3′ UTR) was found in two geospatially distinct metagenomic datasets from *Trichodesmium*-dominated marine communities. These include loci on a single long contig from Oahu, Hawaii (IMG Submission ID 8735, TrichMGDRAFT_c100672) and on four combined contigs from Bermuda (IMG Submission ID 2682, TCCM_contig02077+ contig01776+ contig 01525+ contig 02736)^4^. A full alignment with the exon-twintron-exon sequence from *T. erythraeum* (Suppl. Alignment 4) revealed a nucleotide identity of 95.5% between the two metagenomic sequences, while the *T. erythraeum* sequence was only 78.1% and 81.6% identical with the Hawaii and Bermuda sequences, respectively. Interestingly, the IEP ORF of the inner intron in *T. erythraeum* is shorter than the corresponding ORF in the metagenomes (489 instead of 596 amino acids) due to two SNPs producing a stop codon. The following sequence until the stop codon of the full-length IEP exhibits multiple SNPs as well as deletions in *T. erythraeum* compared to the metagenomics data, indicating the degeneration of this former coding sequence with concurrent conservation of the following intron sequence. The situation of the host intron IEP ORF is similar (449 aa instead of 472 aa), with a premature stop codon generated by an insertion in *T. erythraeum*. However, no conserved domain is lost in *T. erythraeum* through these shortened ORFs. We conclude that the localization of the twintron to the *Tery_4732*+ host gene is conserved within the genus *Trichodesmium* but not found outside of it.

### Characterization of the mature host intron, the inner intron and the splice reactions

The complex genetic arrangement and its conservation within the genus *Trichodesmium* raises questions about the order and biochemical details of the splice reactions. To complement the computationally predicted secondary structures of Tr.e.I7 and Tr.e.I8^15^, we created structure models using the Mfold algorithm and annotated them taking the known facts about group II intron consensus structures^19^,^20^,^21^ into account. We identified most putative tertiary interaction sites and added the insertion site of the inner intron to the model of Tr.e.I7 (Supplementary Fig. S1).

Both introns show the typical structure for a group II intron, consisting of six domains and a bulging adenosine residue in domain VI. The host intron (Supplementary Fig. S1A) was classified to belong to subgroup B2 based on the 5′-terminal consensus sequence GUGCGAUUC, a 62 nt insertion in the basal stem of domain 1, IIB-typical λ and ε′ elements, the exon binding site (EBS) 2 being stem-less, the presence of an EBS3, the absence of a linker between domains III and IV, and some group IIB2 specific conserved nucleotides. The structure displays an atypically large domain II of 536 nt, as well as a large domain IV of 215 nt plus the IEP ORF. The δ- δ′ regions are not compatible (A-A), thus atypical 3′ splice site selection might occur.

The inner intron (Supplementary Fig. S1B) fits most criteria of subgroup B1 and also has a large domain IV of 317 nt plus the IEP ORF. Notably, the catalytic domain V appears atypical, as does the corresponding λ nucleotide in domain I. The inner intron is situated within domain 1 of the group II host intron, adjacent to the sequence involved in the θ-θ′ tertiary interaction with the basal stem of domain II.

Upon splicing, the vast majority of group II introns yield the mature host RNA and the free intron as a lariat, connected via a 2′–5′ phosphodiester branch at the bulging A^20^. However, some group II introns can splice hydrolytically, producing a linear free intron^22^. In rare cases, true circles have also been observed^23^,^24^, probably also connected via a 2′–5′ phosphodiester bond. The latter splicing reaction involves a free 5′ exon resulting from the spliced exon reopening (SER) reaction^25^ attacking the 3′ splice site. This would produce correctly spliced exons and a 5′ exon-intron intermediate. The free 3′ end of the intron then attacks the 5′ splice site, resulting in a circle instead of a lariat. To determine the splice mechanism of each intron within the twintron, we performed cDNA synthesis on putative intron lariats or circles^26^ using Superscript, a M-MLV-type reverse transcriptase (RNase H-) that is able to read through 2′–5′ diester bonds^27^. Using a reverse transcription primer pointing outwards at the 5′ end of each intron together with a primer pointing outwards at the 3′ end of each intron for subsequent RT-PCR, amplicons were only produced if some sort of circular form existed and reverse transcriptase could read through the ligation point. To avoid experimental artifacts, RNA was pre-treated with ribonuclease R (RNase R) from *E. coli.* RNase R is a 3′ → 5′ exoribonuclease that digests linear RNAs but leaves lariat or circular RNA structures intact^28^,^29^.

**Figure 3:**
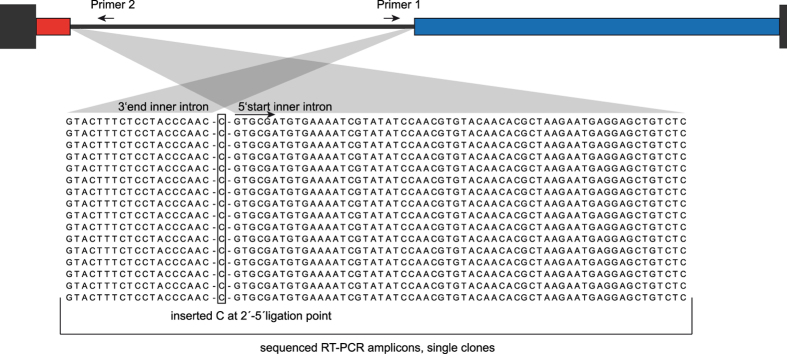
Alignment of RT-PCR products for the excised inner intron. Non-linear excised forms of the inner intron were detected via an outward pointing PCR on cDNA generated with a single outward pointing primer (primer 2). Gaps in the alignment were inserted for clarity. The top panel shows a scheme of the whole twintron as described in Figure 1. All sequencing products showed the inner intron excised as a true circle, not as a lariat, despite the presence of a canonical bulging A (alignment position 13). An additional inserted C (position 21) between the two ends of the intron hints at a 2′–5′ ligation point.

For the inner intron, this method revealed the presence of true circles with single-nucleotide C insertions at the ligation point (Fig. 3). This insertion indicates a 2′–5′ phosphodiester bond because reverse transcriptases tend to stall at these unusual sites and insert one or a few additional nucleotides^26^,^30^. Despite the existence of a canonical bulging A, we could not detect intron lariats, pointing to the circular splicing mechanism, which requires the 5′ product of the SER reaction. In relation to inner intron splicing, this 5′ product would comprise the 5′ mRNA exon and the 1^st^ part of the host intron. If this was the case, we should be able to identify the described intermediate products in a northern blot.

**Figure 4:**
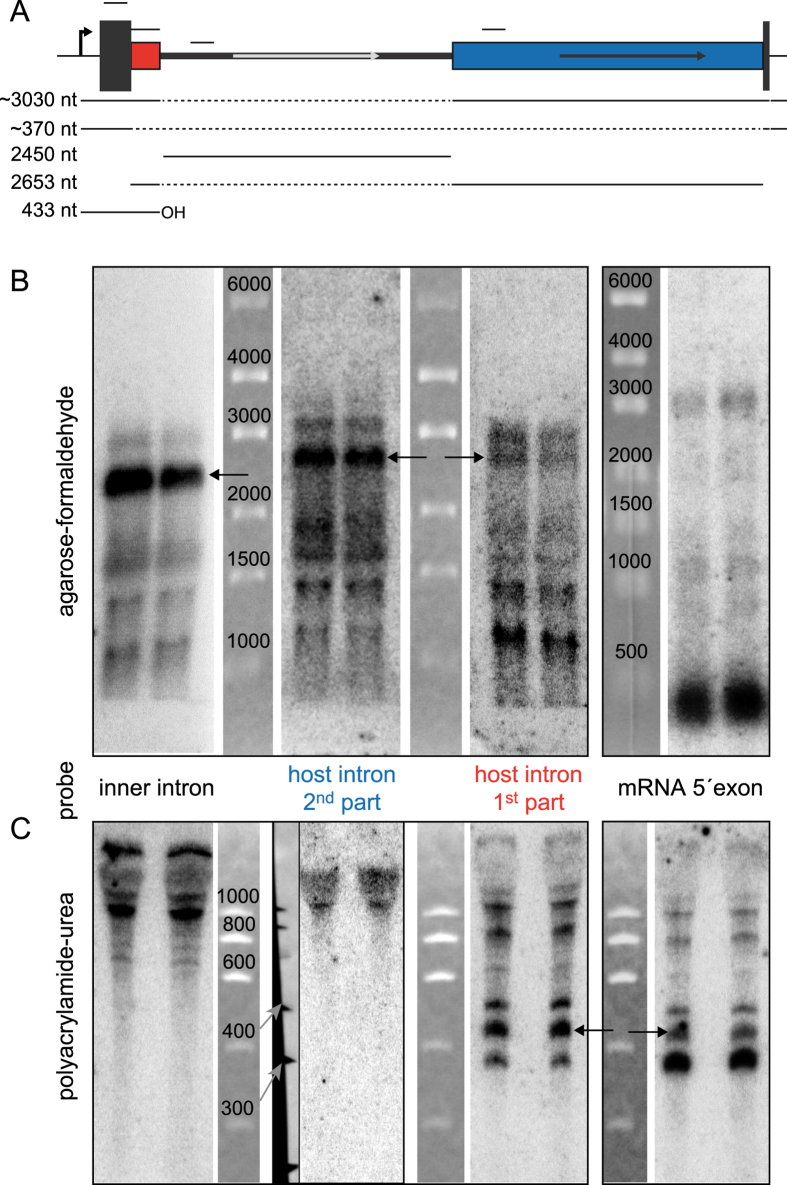
Northern blot analysis of splicing products. (**A**) Twintron scheme with the positions of the four probes used and sizes of likely or expected splicing products; not to scale. The approximated sizes include the estimated 3′ UTR length. (**B**) High molecular weight blots (1000–6000 nt) for all probes. The three intron probes were used on membranes generated from the same gel and are shown in the original order including size markers. All three probes show similar patterns, the respective linear introns are indicated by arrows. The two probes for the host intron show an identical pattern, but with different relative signal intensities. The apparent band pattern around 1500 nt is an artifact generated by the abundant rRNA bands blocking hybridization. The probe for the mRNA 5′-exon (different gel) detected mainly the mature mRNA (main signal) together with some small intermediates, here the most slowly migrating band is likely the intermediate precursor of ~3,030 nt, which was also detected with both host intron probes. (**C**) Low molecular weight, high-resolution blots (300–1000 nt) with the same probes. The first, third, and fourth panels show blots originating from the same 5% PAA gel, whereas the second panel was from a different 6% PAA gel, hence the additional marker shown next to it. The probe for the 1^st^ part of the host intron detected a prominent RNA at ~430 nt, which was also detectable with the 5′-exon probe (black arrows) and represents the free 5′exon generated by SER for inner intron splicing. The main signal detected with the 5′-exon probe was again the mature mRNA. The size of ~370 nt lead to the estimation of a ~60 nt 3′UTR.

For the host intron, we did not succeed in amplifying a free circular or lariat intron. To gain information about spliced products for the host intron and also to verify the splice products for the inner intron, we performed northern blots using four different probes (Fig. 4A) to obtain information about the relative abundance of different splicing intermediates and products. One probe each covered the 5′ exon, the host intron upstream and downstream of the inner intron, and the inner intron. All three intron probes were positioned upstream of the respective ORFs. Blots for high and low molecular weight (MW) RNA were performed separately. In the high MW blots, all intron probes showed similar band patterns with the respective linear intron as the most prominent band and faster migrating degradation products (Fig. 4B). The most slowly migrating band in each of these blots might be the above identified circular intron for the inner intron probe and the intermediate precursor detected with both host intron probes and the 5′ exon probe. Thus, a linear form of both introns was present *in vivo* in addition to the circular inner intron, whereas the full-length pre-mRNA with a hypothetical length of at least 5,436 nt (without putative 3′UTR) was not seen, indicating rapid splicing. In the low MW blots we identified an RNA that is most likely the 5′ product of the SER reaction (Fig. 4C, black arrows). This 433 nt long 5′ product, consisting of the 5′ mRNA exon and the 1^st^ part of the host intron with terminal -OH, is needed to initiate the above-described true circle splicing of the inner intron^23^, and it was detected with both the 5′ exon and the host intron 1^st^ part probes. As expected, no smaller RNAs were detected with probes in the inner intron or 2^nd^ part of the host intron.

The northern blot results are consistent with the presence of true circles for the excised inner intron and revealed the presence of linear free inner and host introns *in vivo*. With the 5′ exon probe, we readily detected the mature mRNA (~370 nt) including its 5′ UTR of 43 nt as determined by the genome-wide mapping of transcriptional start sites^3^ and a putative 3′ UTR of ~60 nt, and an intermediate precursor without the inner intron (3,030 nt). Thus, these results confirm the functional and sequential splicing of the full twintron.

## Discussion

Twintrons were first found in *Euglena gracilis* chloroplast genes^31^, but they also occur with low frequency in mitochondrial^32^ and eukaryotic nuclear genes^33^, and in prokaryotic genomes, where they are thought to be mainly restricted to intergenic regions^34^,^35^. It is assumed that twintrons evolve through recombination of a mobile intron into the target DNA site, which in this case would be another intron. It is further assumed that there is selective pressure for insertion of the inner intron into a site essential for splicing of the host intron because this ensures maintenance of the inner intron^31^. The complex splicing necessary for correct twintron removal from the pre-mRNA might, if error-prone, be a reason for negative selection of twintron arrangements interrupting protein-coding genes in bacteria. As of today, the twintron we analyzed in this work is the only twintron arrangement of this kind in bacteria. Despite the fact that components of other bacterial twintrons have been tested for splicing activity and mobility *ex vivo*^14^, sequential twintron splicing has nonetheless not been reported for bacteria thus far.

The genome of *T. erythraeum* possesses an extensive number of pseudogenes^2^. Therefore, experimental analyses are essential to determine whether an intron-interrupted gene is expressed and functional or merely a remnant of recent genome expansion. We provide evidence that the mixed group II-twintron Tr.e.I7-Tr.e.I8 and its host gene are indeed transcribed and correctly and sequentially spliced in a culture of *T. erythraeum*. Despite the strong degree of conservation of its target gene *Tery_4732*+, constituting a conserved putative intron propagation site in the cyanobacterial phylum (compare host intron IBS in Suppl. Fig. 1A and Suppl. Alignment 3), neither this twintron nor a single intron occurs in this gene in any other genome outside of the genus *Trichodesmium*, indicating that a natural barrier exists for the proliferation of the host intron.

Compared to their occurrence in organelles, group II introns are relatively rare in bacteria in general. They can be detected in approximately 25% of eubacterial genomes and mostly in low numbers^19^. This might be the main reason for the extreme rarity of twintrons in bacteria as well. However, some bacteria possess an elevated number of group II introns. In the cyanobacterial phylum, 28 group II introns were described in the thermophilic *Thermosynechococcus elongatus,* all phylogenetically related and located mostly in non-coding parts of the genome^14^,^36^ and at least 150 exist in the filamentous cyanobacterium *Arthrospira* (*Spirulina*) *platensis*^37^. Indeed, in *Thermosynechococcus elongatus* four twintrons were reported, none of them connected to flanking exons that encode an ORF^35^. Intron-rich *T. erythraeum*, the host genome of the twintron analyzed in this work, has recently been shown to have a high number of *in vivo* splicing group II introns^3^, many of which are located in protein-coding genes, despite the high non-coding percentage of this genome. The database for non-redundant bacterial group II introns^15^ lists *T. erythraeum* on rank 1 with seven unique, full-length and putatively functional introns. Multiple occurrences of one unique intron type in the same genome are not listed. This makes *T. erythraeum* the bacterial genome with the highest documented diversity of group II introns. It is an interesting question whether the high non-coding genome fraction of this genome might have facilitated evolution of intron diversity or the other way around.

There is no known homolog to the here investigated twintron in other bacteria. However, we show that the same arrangement as in *T. erythraeum* exists in two metagenomic datasets of geospatially distinct natural *Trichodesmium* populations. Hence, sequential twintron splicing is functional in a bacterial protein-coding gene, without deleterious effects, and the twintron is stably maintained in the genus.

## Materials and Methods

### Culture media and growth conditions

*T. erythraeum* was grown in 500 ml YBCII medium^38^ at 25 °C, 12:12 light/dark cycle at ~80 μmol photons m^−2^s^−1^ white light until cultures were dense but not yet forming puffs or tufts. To test for possible stress effects, 500 ml pre-cultures were sampled and then separately subjected to three different conditions. One culture was placed under high light at 500 μmol photons m^−2^s^−1^ for 15 min and sampled again. To another culture, 10 μM DFB was added and the culture was sampled at 0 h, 7 h, and 24 h. A third culture was washed with phosphate-free medium using gravity flow over a 10 μm polycarbonate filter, then sampled at 0 h, 7 h, and 24 h.

### RNA extraction

100 ml of a culture was filtered over a polyethersulfone filter (PALL Supor, 0.8 μm, 47 mm), then placed immediately in a 2 ml reaction tube containing 1 ml phenolic PGTX extraction buffer^39^ and 250 μl glass beads (0.10–0.25 mm), frozen in liquid nitrogen, and kept at −80 °C until extraction. Prior to extraction, samples were thawed on ice, mechanically opened in a Precellys cell disruptor for 3 × 20 s at 6500 rpm, and then placed in a 65 °C water bath for 15 min, vortexing regularly. Phase extraction of RNA was performed as described previously^40^, and RNA was dissolved in 30 μl of nuclease-free H_2_O.

### Reverse transcription (RT) PCR and lariat RT-PCR

All oligonucleotides are listed in Supplementary Table 1. DNA-free total IMS101 RNA was reverse transcribed with three specific reverse primers (#1, 2, 3) to account for the very long precursor RNA of the twintron-containing RNase HI gene to yield cDNAs covering all intron/intron and intron/exon boundaries. All reverse transcriptions were performed with the QuantiTect Reverse Transcription kit (Qiagen, Hilden, Germany) following the manufacturer’s instructions with 30 min reaction time. The resulting cDNA was used in a 1:10 dilution for RT-PCR with the following primer combinations: 2/7 for the sequence around the 3′ splice site of the inner intron, 2/6 for detection of the mature host intron, 4/5 for detection of the mature mRNA, 3/6 for the sequence around the 5′ splice site of the inner intron, 5/8 for the sequence around the 5′ splice site of the host intron, and 5/2 for detection of the intermediate precursor without the inner intron.

For lariat RT-PCR, primers for reverse transcription were positioned right after the 5′ splice sites of both the inner and host introns. RNA was treated with 1 ul (20 units) RNase R (Epicentre, Madison, USA) prior to cDNA synthesis for 1 h at 37dC to digest all linear RNA molecules. cDNA was then synthesized using the Superscript IV First Strand Synthesis Kit (Life Technologies, Gaithersburg, MD, USA), with reverse primers pointing out of the intron (#3, 8). The same rev-primer and a fw-primer pointing outwards at the other end of the respective intron (#7, 9) were then used for PCR. The amplicons were cloned into *E. coli* TOP10F’ and individual clones sequenced. Various different fw-primers (#10, 12) together with a nested rev-primer (#11) were used in attempts to amplify the host intron lariat (or circle), but none succeeded, suggesting that these exist in very low numbers if at all.

### Northern blot analysis

Blots were prepared from the separation of 4–5 μg of total RNA on 5% or 6% polyacrylamide urea gels or 1.5% agarose gels with 18% formaldehyde depending on the expected transcript sizes (up to 1,000 nt and above 1,000 nt, respectively). NorthernMax® Formaldehyde Load Dye (Life Technologies) was added to samples in 2× or 3× concentration and incubated for 15 min at 65 °C to enhance denaturation of RNA samples. As running buffer for formaldehyde-agarose gels, 1x MOPS buffer (40 mM 3-(N-morpholino) propanesulfonic acid, pH 7.0, 10 mM sodium acetate, 1 mM EDTA) with 1/50 v/v of 37% formaldehyde was used. Blotting and hybridization with [α-32P]UTP-labeled transcript probes was performed as previously described^40^. Transcript probes were produced from PCR-generated templates (oligonucleotides #13–20) using *in vitro* transcription (MaxiScript kit, Ambion). All DNA oligonucleotides used for PCR and hybridization experiments were purchased from Sigma-Aldrich (Hamburg, Germany) or Life Technologies (Darmstadt, Germany) at the ‘desalted’ quality (Supplementary Table S1). The working concentration of all oligonucleotides was 10 μM.

### *In silico* analyses

BLASTP and TBLASTN together with the NCBI nt and nr databases were used to identify RNase HI genes and homologues of *Tery_4732*+ (twintron host gene) and gain information about which genomes encode RNase HI and the *Tery_4732*+ homolog as a hybrid protein and which encode them separately. Additionally, genes annotated as RNase HI, but not found by BLAST, were searched for in NCBI. For phylogenetic tree building of the *Tery_4732*+ and RNase HI (*Tery_1584*) homologs, we used the IMG/MER database to select all genomes containing separately encoded RNase HI and *Tery_4732*+ homologs and complemented this list with additional genomes from the cyanobacterial phylum (carrying hybrids of these two genes) and other marine bacteria. The two protein sequences from *T. erythraeum* were then used for a BLASTP search in this genome list with an e-value cutoff of e-8 and the nucleotide sequences of the resulting genes exported. Using ClustalW, an initial alignment was done for each set of homologs. This was used to trim longer sequences (the hybrid proteins) roughly from each of the two alignments. The resulting sequences were de-gapped and the final alignment calculated using ProbCons with default parameters and 2 pretraining rounds^41^. The resulting alignment was used for a phylogenetic analysis with Mr. Bayes version 3.2.5^42^ for Unix with the following parameter set: lset Nucmodel = 4by4 Nst = 6 Ploidity = Haploid Rates = Gamma, and the default model priors and run settings (with 2,000,000 generations).

Intron RNA sequences were folded into secondary structures with MFold^43^ using the current web-based MFold tool at http://unafold.rna.albany.edu/? q = mfold/RNA-Folding-Form (February 2015) with default parameters (37 °C folding temperature). Domain I of each of the two introns was folded separately from the rest. IEP ORFs were replaced by ten Ns for folding. If the best structure (smallest initial dG) was not yielding the 6 domains (as known for group II introns), minimal constrains were used based on known consensus structures. For the host intron, the initial best fold with a revised dG of −333.25 kcal/mol perfectly fit the consensus. For the inner intron, the following constraints were used: P 1 0 5, F 64 83 3, P 250 0 4, F 498 583 2, prohibiting pairing of the first 5 nt of the intron and of the β′ interaction site, forcing the basal stem of the α tertiary interaction loop in DI and of the internal GC stem in DIII. This decreased the revised dG from −251.40 to −259.88 kcal/mol, indicating that the constrained structure is even better but was not found by the folding algorithm. Structures were imported into VARNA^44^ for improved visualization, and finalized in Adobe Illustrator.

## Additional Information

**How to cite this article**: Pfreundt, U. and Hess, W. R. Sequential splicing of a group II twintron in the marine cyanobacterium *Trichodesmium*. *Sci. Rep.* **5**, 16829; doi: 10.1038/srep16829 (2015).

## Supplementary Material

Supplementary Materials
