# Supplementary material for: Sequential splicing of a group II twintron in the marine cyanobacterium Trichodesmium
Source: Sci Rep. 2015 Nov 18;5:16829. doi: 10.1038/srep16829 (PMC4649490; doi:10.1038/srep16829)

Supplementary Material

Sequential splicing of a group II twintron in the marine cyanobacterium *Trichodesmium*

Ulrike Pfreundt and Wolfgang R. Hess

University of Freiburg, Faculty of Biology, Schänzlestr. 1, D-79104 Freiburg, Germany

*Corresponding author:

Prof. Wolfgang R. Hess, University Freiburg, Faculty of Biology, Inst. Biology III, Schänzlestr. 1, D-79104 Freiburg, Germany

Tel. +49-(0)761-203-2796

FAX: +49-(0)761-203-2745

Email: wolfgang.hess@biologie.uni-freiburg.de

Supplementary Figure 1

RNA secondary structure of the group II host intron and the inner intron comprising the Tery_4732 twintron.

A) Mature host intron. The insertion site of the inner intron, within domain I (DI), and the IEP ORF situated in domain IV, are marked. A large extension of 250 nt within DII is indicated by a dashed loop. Structure prediction led to the classification of this intron as group II B2. Greek letters mark sequence regions that pair to form tertiary structures (for example β and β'). The bulging A in domain VI that usually initiates the first nucleophilic attack on the intron-exon boundary is colored red. EBS = exon binding site, IBS = intron binding site. Base pairs are indicated by gray dots; reds dots indicate paired bases that fit the consensus better if not paired.

B) Inner intron. Annotation is the same as for the host intron. This intron was classified as group II B1. Notably, DV does not fit the consensus structure; the 5-nt loop is atypical, so are λ and λ´ including the adjacent bulge.

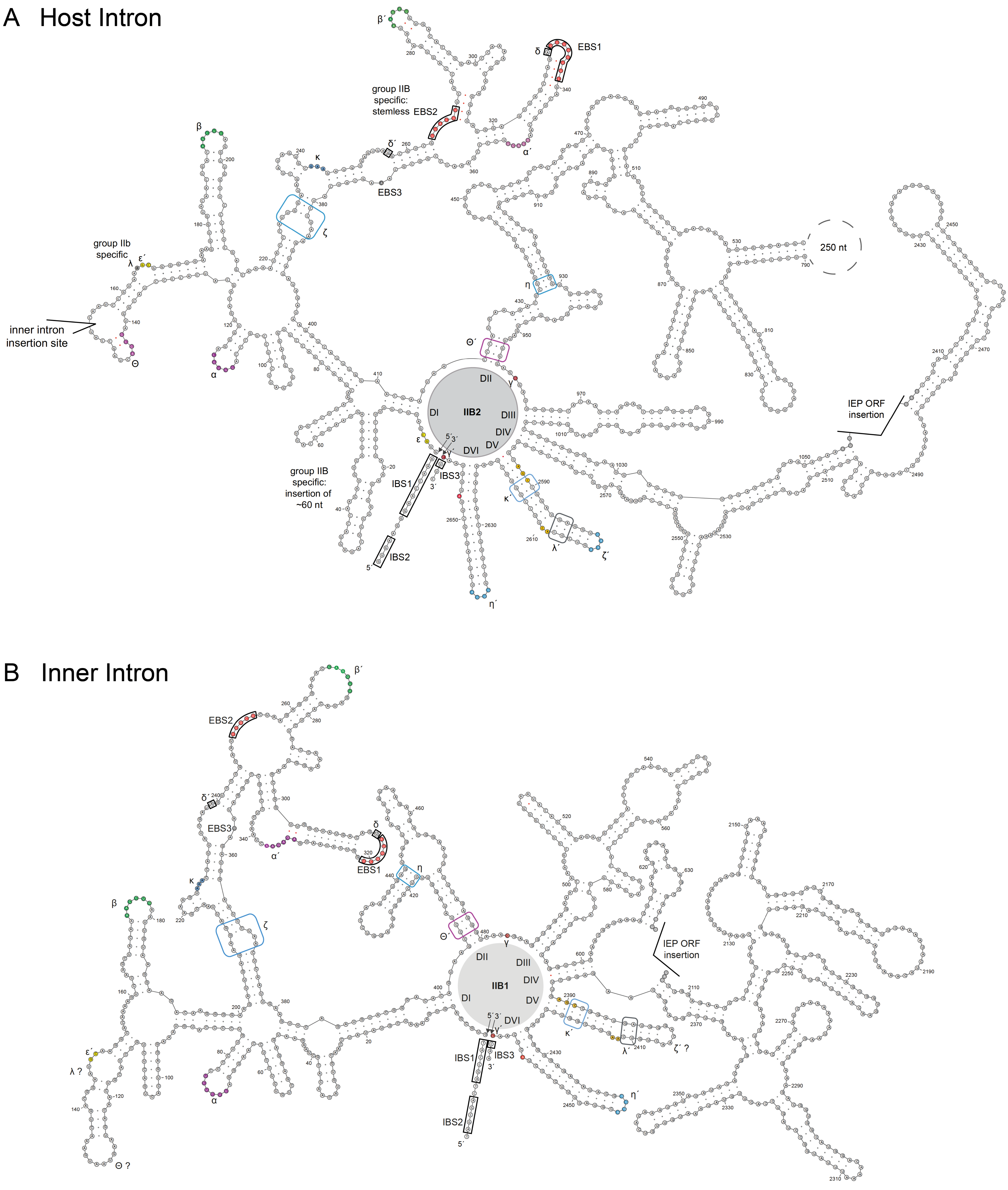

A Host Intron
β´
EBS1
δ
group IIB
specific:
stemless EBS2
δ´
κ
EBS3
β
ζ
group IIb
specific
λ
ε´
inner intron
insertion site
Θ
α
α´
250 nt
η
Θ´
DII
γ
IIB2
DI
DIII
DIV
DV
DVI
ε
5´
3´
IBS1
IBS3
group IIB
specific:
insertion of
~60 nt
IBS2
κ
λ´
ζ´
η´
IEP ORF
insertion
B Inner Intron
β´
EBS2
δ´
EBS3
α´
δ
EBS1
η
κ
β
ζ
Θ´
γ
IIB1
DII
DIII
DIV
DV
DI
DVI
IEP ORF
insertion
ε´
λ ?
Θ ?
α
IBS1
IBS3
IBS2
5´
3´
κ´
λ´
ζ´ ?
η´

Supplementary Alignment 1

Alignment of all full-length cyanobacterial hybrid RNase HI proteins together with all separately encoded RNase HI and *Tery_4732*+ homologs that were concatenated for this alignment. To distinguish the latter, the term “concat” was added to the species name. Note that all proteins start on page 1 and the alignment is continued on the following pages. Species are sorted alphabetically. The amino acid similarity is color-coded, the darker the more similar.

The first part of the protein is reasonably well conserved (page 1-3), followed by a putative linker region of varying length and sequence. The C-terminal part (page 6), corresponding to *Tery_4732*+, the host gene of the *T. erythraeum* twintron, is highly conserved in all species except the picocyanobacteria and *Gloeobacter*.

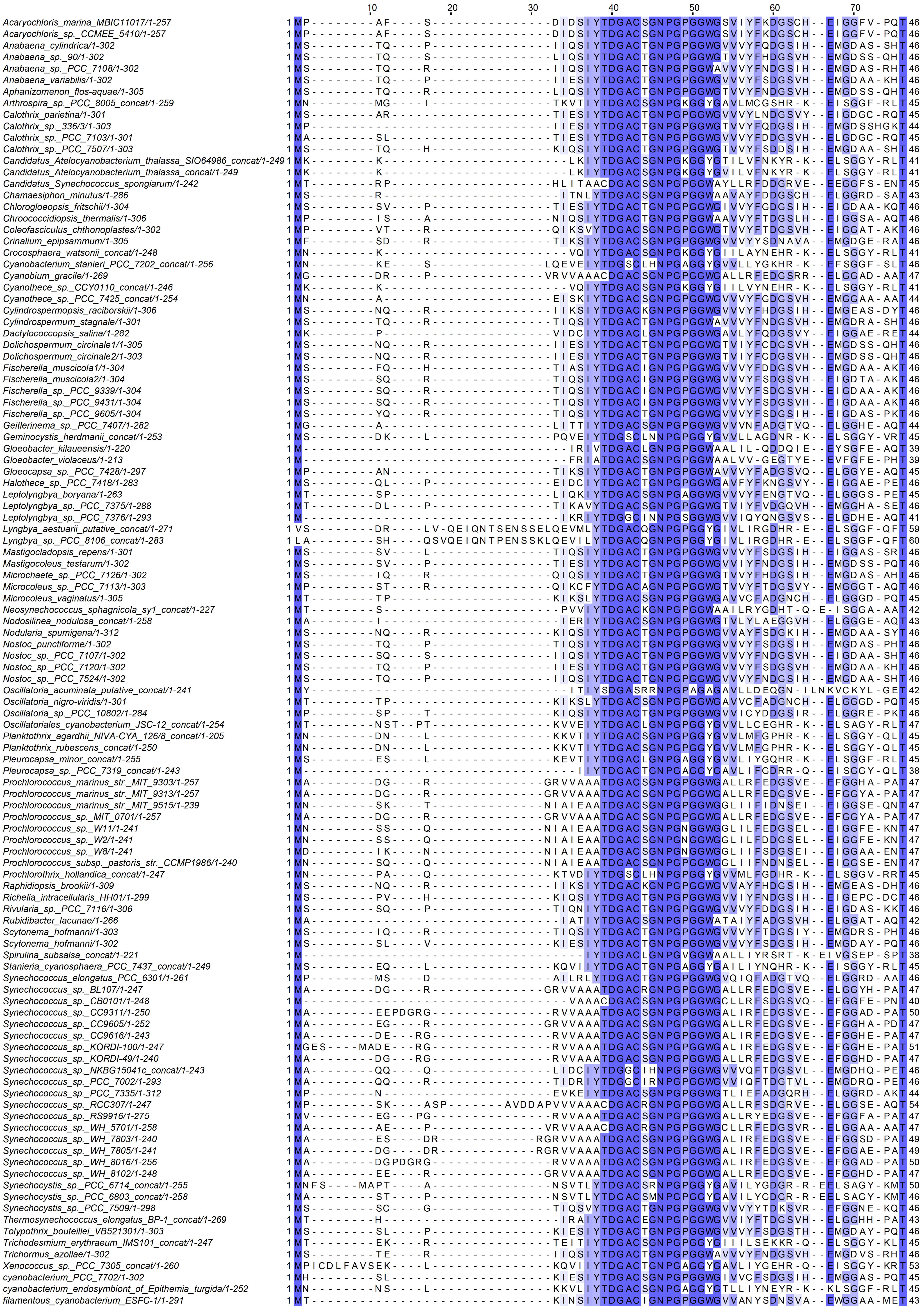

10
20
30
40
50
60
70
Acaryochloris_marina_MBIC11017/1-257
Acaryochloris_sp._CCMEE_5410/1-257
Anabaena_cylindrica/1-302
Anabaena_sp._90/1-302
Anabaena_sp._PCC_7108/1-302
Anabaena_variabilis/1-302
Aphanizomenon_flos-aquae/1-305
Arthrospira_sp._PCC_8005_concat/1-259
Calothrix_parietina/1-301
Calothrix_sp._336/3/1-303
Calothrix_sp._PCC_7103/1-301
Calothrix_sp._PCC_7507/1-303
Candidatus_Atelocyanobacterium_thalassa_SIO64986_concat/1-249
Candidatus_Atelocyanobacterium_thalassa_concat/1-249
Candidatus_Synechococcus_spongiarum/1-242
Chamaesiphon_minutus/1-286
Chlorogloeopsis_fritschii/1-304
Chroococcidiopsis_thermalis/1-306
Coleofasciculus_chthonoplastes/1-302
Crinalium_epipsammum/1-305
Crocosphaera_watsonii_concat/1-248
Cyanobacterium_stanieri_PCC_7202_concat/1-256
Cyanobium_gracile/1-269
Cyanothece_sp._CCY0110_concat/1-246
Cyanothece_sp._PCC_7425_concat/1-254
Cylindrospermopsis_raciborskii/1-306
Cylindrospermum_stagnale/1-301
Dactylococcopsis_salina/1-282
Dolichospermum_circinale1/1-305
Dolichospermum_circinale2/1-303
Fischerella_muscicola1/1-304
Fischerella_muscicola2/1-304
Fischerella_sp._PCC_9339/1-304
Fischerella_sp._PCC_9431/1-304
Fischerella_sp._PCC_9605/1-304
Geitlerinema_sp._PCC_7407/1-282
Geminocystis_herdmanii_concat/1-253
Gloeobacter_kilaueensis/1-220
Gloeobacter_violaceus/1-213
Gloeocapsa_sp._PCC_7428/1-297
Halothece_sp._PCC_7418/1-283
Leptolyngbya_boryana/1-263
Leptolyngbya_sp._PCC_7375/1-288
Leptolyngbya_sp._PCC_7376/1-293
Lyngbya_aestuarii_putative_concat/1-271
Lyngbya_sp._PCC_8106_concat/1-283
Mastigocladopsis_repens/1-301
Mastigocoleus_testarum/1-302
Microchaete_sp._PCC_7126/1-302
Microcoleus_sp._PCC_7113/1-303
Microcoleus_vaginatus/1-305
Neosynechococcus_sphagnicola_sy1_concat/1-227
Nodosilinea_nodulosa_concat/1-258
Nodularia_spumigena/1-312
Nostoc_punctiforme/1-302
Nostoc_sp._PCC_7107/1-302
Nostoc_sp._PCC_7120/1-302
Nostoc_sp._PCC_7524/1-302
Oscillatoria_acuminata_putative_concat/1-241
Oscillatoria_nigro-viridis/1-301
Oscillatoria_sp._PCC_10802/1-284
Oscillatoriales_cyanobacterium_JSC-12_concat/1-254
Planktothrix_agardhii_NIVA-CYA_126/8_concat/1-205
Planktothrix_rubescens_concat/1-250
Pleurocapsa_minor_concat/1-255
Pleurocapsa_sp._PCC_7319_concat/1-243
Prochlorococcus_marinus_str._MIT_9303/1-257
Prochlorococcus_marinus_str._MIT_9313/1-257
Prochlorococcus_marinus_str._MIT_9515/1-239
Prochlorococcus_sp._MIT_0701/1-257
Prochlorococcus_sp._W11/1-241
Prochlorococcus_sp._W2/1-241
Prochlorococcus_sp._W8/1-241
Prochlorococcus_subsp._pastoris_str._CCMP1986/1-240
Prochlorothrix_hollandica_concat/1-247
Raphidiopsis_brookii/1-309
Richelia_intracellularis_HH01/1-299
Rivularia_sp._PCC_7116/1-306
Rubidibacter_lacunae/1-266
Scytonema_hofmanni/1-303
Scytonema_hofmanni/1-302
Spirulina_subsalsa_concat/1-221
Stanieria_cyanosphaera_PCC_7437_concat/1-249
Synechococcus_elongatus_PCC_6301/1-261
Synechococcus_sp._BL107/1-247
Synechococcus_sp._CB0101/1-248
Synechococcus_sp._CC9311/1-250
Synechococcus_sp._CC9605/1-252
Synechococcus_sp._CC9616/1-243
Synechococcus_sp._KORDI-100/1-247
Synechococcus_sp._KORDI-49/1-240
Synechococcus_sp._NKBG15041c_concat/1-243
Synechococcus_sp._PCC_7002/1-293
Synechococcus_sp._PCC_7335/1-312
Synechococcus_sp._RCC307/1-247
Synechococcus_sp._RS9916/1-275
Synechococcus_sp._WH_5701/1-258
Synechococcus_sp._WH_7803/1-240
Synechococcus_sp._WH_7805/1-241
Synechococcus_sp._WH_8016/1-256
Synechococcus_sp._WH_8102/1-248
Synechocystis_sp._PCC_6714_concat/1-255
Synechocystis_sp._PCC_6803_concat/1-258
Synechocystis_sp._PCC_7509/1-298
Thermosynechococcus_elongatus_BP-1_concat/1-269
Tolypothrix_bouteillei_VB521301/1-303
Trichodesmium_erythraeum_IMS101_concat/1-247
Trichormus_azollae/1-302
Xenococcus_sp._PCC_7305_concat/1-260
cyanobacterium_PCC_7702/1-302
cyanobacterium_endosymbiont_of_Epithemia_turgida/1-252
filamentous_cyanobacterium_ESFC-1/1-291

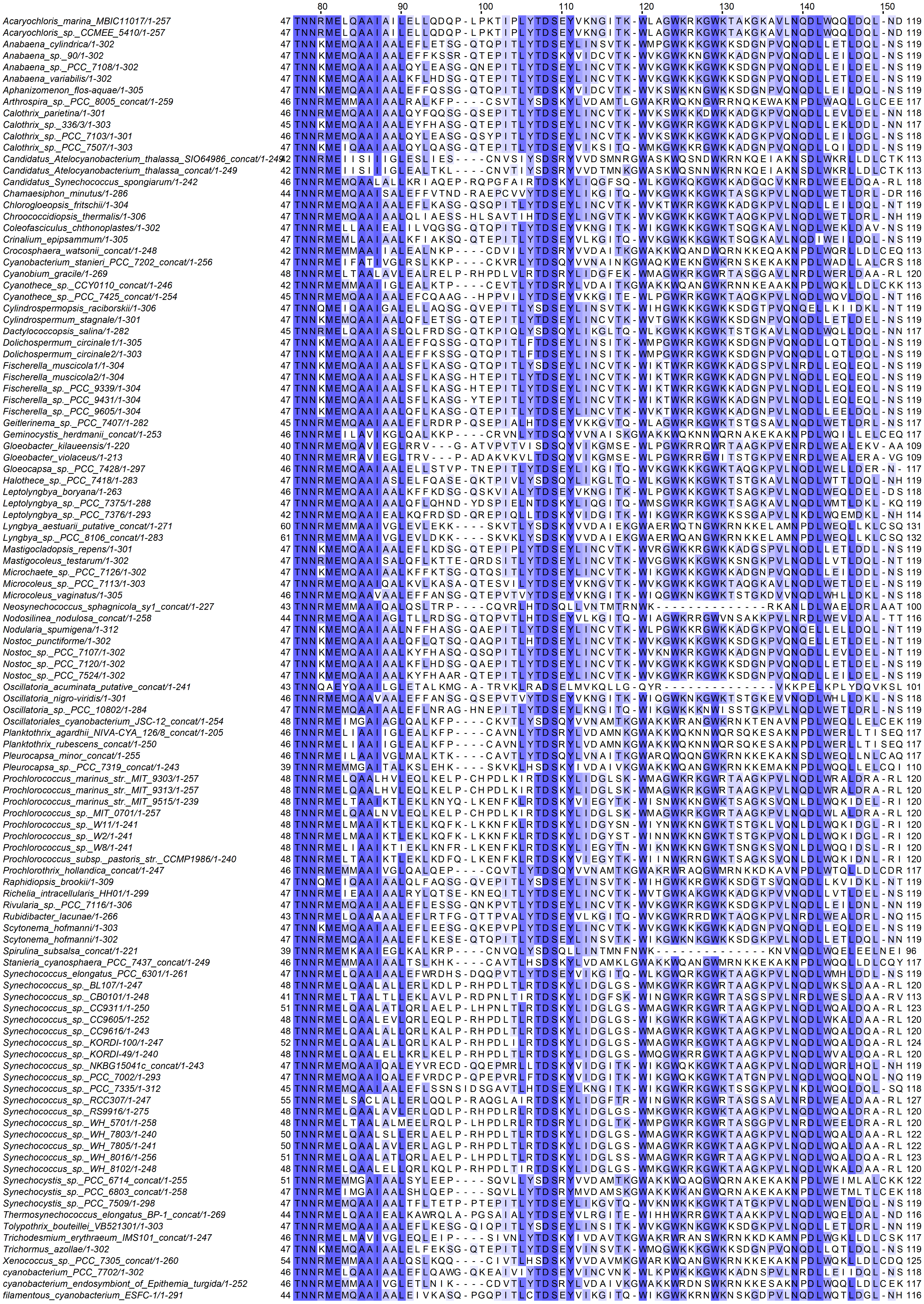
80 90 100 110 120 130 140 150
Acaryochloris_marina_MBIC11017/1-257
Acaryochloris_sp._CCMEE_5410/1-257
Anabaena_cylindrica/1-302
Anabaena_sp._90/1-302
Anabaena_sp._PCC_7108/1-302
Anabaena_variabilis/1-302
Aphanizomenon_flos-aquae/1-305
Arthrospira_sp._PCC_8005_concat/1-259
Calothrix_parietina/1-301
Calothrix_sp._336/3/1-303
Calothrix_sp._PCC_7103/1-301
Calothrix_sp._PCC_7507/1-303
Candidatus_Atelocyanobacterium_thalassa_SIO64986_concat/1-249
Candidatus_Atelocyanobacterium_thalassa_concat/1-249
Candidatus_Synechococcus_spongiarum/1-242
Chamaesiphon_minutus/1-286
Chlorogloeopsis_fritschii/1-304
Chroococcidiopsis_thermalis/1-306
Coleofasciculus_chthonoplastes/1-302
Crinalium_epipsammum/1-305
Crocosphaera_watsonii_concat/1-248
Cyanobacterium_stanieri_PCC_7202_concat/1-256
Cyanobium_gracile/1-269
Cyanothece_sp._CCY0110_concat/1-246
Cyanothece_sp._PCC_7425_concat/1-254
Cylindrospermopsis_raciborskii/1-306
Cylindrospermum_stagnale/1-301
Dactylococcopsis_salina/1-282
Dolichospermum_circinale1/1-305
Dolichospermum_circinale2/1-303
Fischerella_muscicola1/1-304
Fischerella_muscicola2/1-304
Fischerella_sp._PCC_9339/1-304
Fischerella_sp._PCC_9431/1-304
Fischerella_sp._PCC_9605/1-304
Geitlerinema_sp._PCC_7407/1-282
Geminocystis_herdmanii_concat/1-253
Gloeobacter_kilaueensis/1-220
Gloeobacter_violaceus/1-213
Gloeocapsa_sp._PCC_7428/1-297
Halothece_sp._PCC_7418/1-283
Leptolyngbya_boryana/1-263
Leptolyngbya_sp._PCC_7375/1-288
Leptolyngbya_sp._PCC_7376/1-293
Lyngbya_aestuarii_putative_concat/1-271
Lyngbya_sp._PCC_8106_concat/1-283
Mastigocladopsis_repens/1-301
Mastigocoleus_testarum/1-302
Microchaete_sp._PCC_7126/1-302
Microcoleus_sp._PCC_7113/1-303
Microcoleus_vaginatus/1-305
Neosynechococcus_sphagnicola_sy1_concat/1-227
Nodosilinea_nodulosa_concat/1-258
Nodularia_spumigena/1-312
Nostoc_punctiforme/1-302
Nostoc_sp._PCC_7107/1-302
Nostoc_sp._PCC_7120/1-302
Nostoc_sp._PCC_7524/1-302
Oscillatoria_acuminata_putative_concat/1-241
Oscillatoria_nigro-viridis/1-301
Oscillatoria_sp._PCC_10802/1-284
Oscillatoriales_cyanobacterium_JSC-12_concat/1-254
Planktothrix_agardhii_NIVA-CYA_126/8_concat/1-205
Planktothrix_rubescens_concat/1-250
Pleurocapsa_minor_concat/1-255
Pleurocapsa_sp._PCC_7319_concat/1-243
Prochlorococcus_marinus_str._MIT_9303/1-257
Prochlorococcus_marinus_str._MIT_9313/1-257
Prochlorococcus_marinus_str._MIT_9515/1-239
Prochlorococcus_sp._MIT_0701/1-257
Prochlorococcus_sp._W11/1-241
Prochlorococcus_sp._W2/1-241
Prochlorococcus_sp._W8/1-241
Prochlorococcus_subsp._pastoris_str._CCMP1986/1-240
Prochlorothrix_hollandica_concat/1-247
Raphidiopsis_brookii/1-309
Richelia_intracellularis_HH01/1-299
Rivularia_sp._PCC_7116/1-306
Rubidibacter_lacunae/1-266
Scytonema_hofmanni/1-303
Scytonema_hofmanni/1-302
Spirulina_subsalsa_concat/1-221
Stanieria_cyanosphaera_PCC_7437_concat/1-249
Synechococcus_elongatus_PCC_6301/1-261
Synechococcus_sp._BL107/1-247
Synechococcus_sp._CB0101/1-248
Synechococcus_sp._CC9311/1-250
Synechococcus_sp._CC9605/1-252
Synechococcus_sp._CC9616/1-243
Synechococcus_sp._KORDI-100/1-247
Synechococcus_sp._KORDI-49/1-240
Synechococcus_sp._NKBG15041c_concat/1-243
Synechococcus_sp._PCC_7002/1-293
Synechococcus_sp._PCC_7335/1-312
Synechococcus_sp._RCC307/1-247
Synechococcus_sp._RS9916/1-275
Synechococcus_sp._WH_5701/1-258
Synechococcus_sp._WH_7803/1-240
Synechococcus_sp._WH_7805/1-241
Synechococcus_sp._WH_8016/1-256
Synechococcus_sp._WH_8102/1-248
Synechocystis_sp._PCC_6714_concat/1-255
Synechocystis_sp._PCC_6803_concat/1-258
Synechocystis_sp._PCC_7509/1-298
Thermosynechococcus_elongatus_BP-1_concat/1-269
Tolypothrix_bouteillei_VB521301/1-303
Trichodesmium_erythraeum_IMS101_concat/1-247
Trichormus_azollae/1-302
Xenococcus_sp._PCC_7305_concat/1-260
cyanobacterium_PCC_7702/1-302
cyanobacterium_endosymbiont_of_Epithemia_turgida/1-252
filamentous_cyanobacterium_ESFC-1/1-291

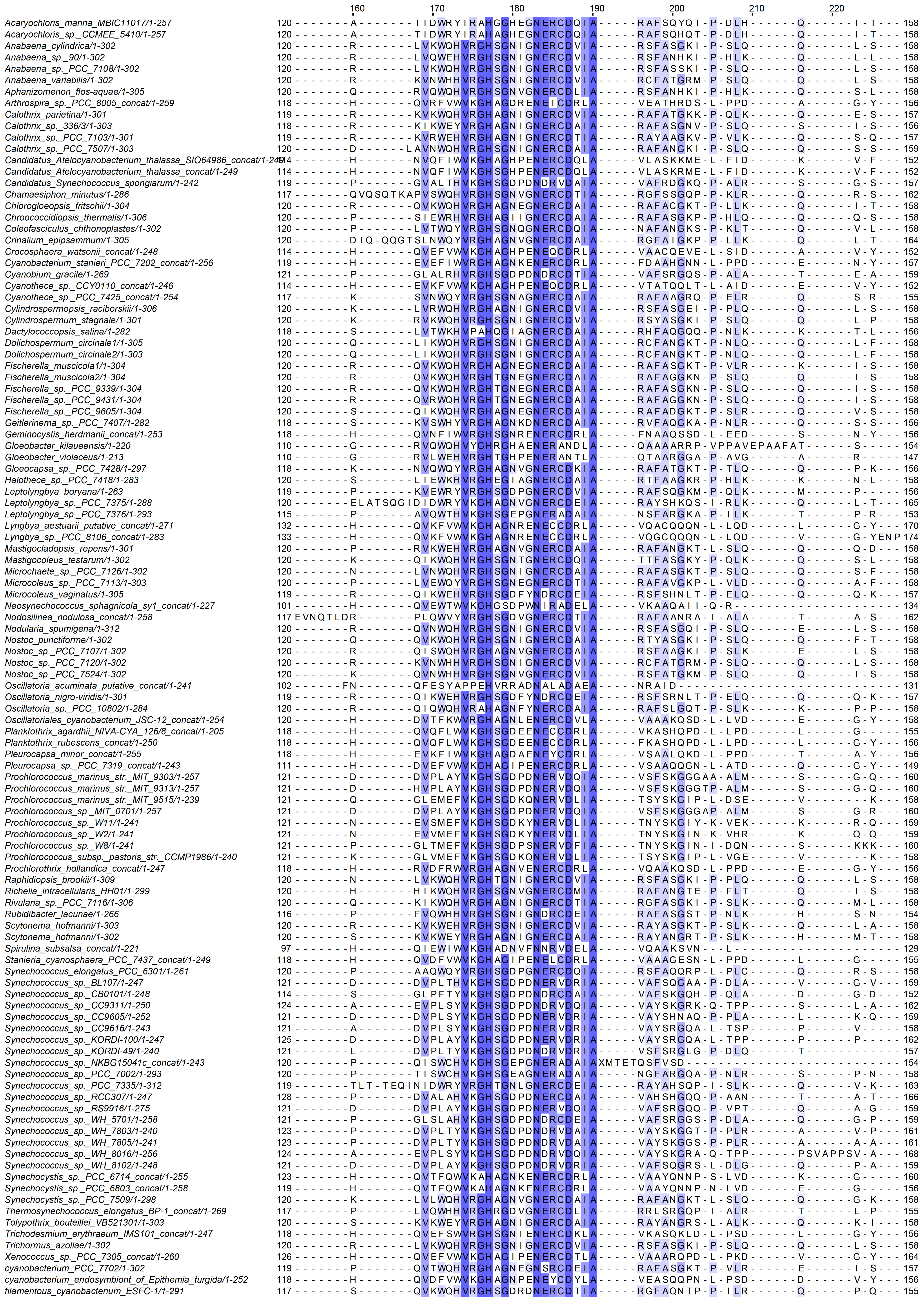
160
170
180
190
200
210
220
Acaryochloris_marina_MBIC11017/1-257 120 158
Acaryochloris_sp._CCMEE_5410/1-257 120 158
Anabaena_cylindrica/1-302 120 158
Anabaena_sp._90/1-302 120 158
Anabaena_sp._PCC_7108/1-302 120 158
Anabaena_variabilis/1-302 120 158
Aphanizomenon_flos-aquae/1-305 120 158
Arthrospira_sp._PCC_8005_concat/1-259 118 156
Calothrix_parietina/1-301 119 157
Calothrix_sp._336/3/1-303 118 156
Calothrix_sp._PCC_7103/1-301 119 157
Calothrix_sp._PCC_7507/1-303 120 159
Candidatus_Atelocyanobacterium_thalassa_SIO64986_concat/1-249 114 152
Candidatus_Atelocyanobacterium_thalassa_concat/1-249 114 152
Candidatus_Synechococcus_spongiarum/1-242 119 157
Chamaesiphon_minutus/1-286 117 162
Chlorogloeopsis_fritschii/1-304 120 158
Chroococcidiopsis_thermalis/1-306 120 158
Coleofasciculus_chthonoplastes/1-302 120 158
Crinalium_epipsammum/1-305 120 164
Crocosphaera_watsonii_concat/1-248 114 152
Cyanobacterium_stanieri_PCC_7202_concat/1-256 119 157
Cyanobium_gracile/1-269 121 159
Cyanothece_sp._CCY0110_concat/1-246 114 152
Cyanothece_sp._PCC_7425_concat/1-254 117 155
Cylindrospermopsis_raciborskii/1-306 120 158
Cylindrospermum_stagnale/1-301 120 158
Dactylococcopsis_salina/1-282 118 156
Dolichospermum_circinale1/1-305 120 158
Dolichospermum_circinale2/1-303 120 158
Fischerella_muscicola1/1-304 120 158
Fischerella_muscicola2/1-304 120 158
Fischerella_sp._PCC_9339/1-304 120 158
Fischerella_sp._PCC_9431/1-304 120 158
Fischerella_sp._PCC_9605/1-304 120 158
Geitlerinema_sp._PCC_7407/1-282 118 156
Geminocystis_herdmanii_concat/1-253 118 156
Gloeobacter_kilaueensis/1-220 110 154
Gloeobacter_violaceus/1-213 110 147
Gloeocapsa_sp._PCC_7428/1-297 118 156
Halothece_sp._PCC_7418/1-283 120 158
Leptolyngbya_boryana/1-263 119 156
Leptolyngbya_sp._PCC_7375/1-288 120 165
Leptolyngbya_sp._PCC_7376/1-293 115 153
Lyngbya_aestuarii_putative_concat/1-271 132 170
Lyngbya_sp._PCC_8106_concat/1-283 133 174
Mastigocladopsis_repens/1-301 120 158
Mastigocoleus_testarum/1-302 120 158
Microchaete_sp._PCC_7126/1-302 120 158
Microcoleus_sp._PCC_7113/1-303 120 158
Microcoleus_vaginatus/1-305 119 157
Neosynechococcus_sphagnicola_sy1_concat/1-227 101 134
Nodosilinea_nodulosa_concat/1-258 117 162
Nodularia_spumigena/1-312 120 158
Nostoc_punctiforme/1-302 120 158
Nostoc_sp._PCC_7107/1-302 120 158
Nostoc_sp._PCC_7120/1-302 120 158
Nostoc_sp._PCC_7524/1-302 120 158
Oscillatoria_acuminata_putative_concat/1-241 102 131
Oscillatoria_nigro-viridis/1-301 119 157
Oscillatoria_sp._PCC_10802/1-284 120 158
Oscillatoriales_cyanobacterium_JSC-12_concat/1-254 120 158
Planktothrix_agardhii_NIVA-CYA_126/8_concat/1-205 118 155
Planktothrix_rubescens_concat/1-250 118 156
Pleurocapsa_minor_concat/1-255 118 156
Pleurocapsa_sp._PCC_7319_concat/1-243 111 149
Prochlorococcus_marinus_str._MIT_9303/1-257 121 160
Prochlorococcus_marinus_str._MIT_9313/1-257 121 160
Prochlorococcus_marinus_str._MIT_9515/1-239 121 158
Prochlorococcus_sp._MIT_0701/1-257 121 160
Prochlorococcus_sp._W11/1-241 121 159
Prochlorococcus_sp._W2/1-241 121 159
Prochlorococcus_sp._W8/1-241 121 160
Prochlorococcus_subsp._pastoris_str._CCMP1986/1-240 121 158
Prochlorothrix_hollandica_concat/1-247 118 156
Raphidiopsis_brookii/1-309 120 158
Richelia_intracellularis_HH01/1-299 120 158
Rivularia_sp._PCC_7116/1-306 120 158
Rubidibacter_lacunae/1-266 116 154
Scytonema_hofmanni/1-303 120 158
Scytonema_hofmanni/1-302 120 158
Spirulina_subsalsa_concat/1-221 97 129
Stanieria_cyanosphaera_PCC_7437_concat/1-249 118 155
Synechococcus_elongatus_PCC_6301/1-261 120 158
Synechococcus_sp._BL107/1-247 121 159
Synechococcus_sp._CB0101/1-248 114 152
Synechococcus_sp._CC9311/1-250 124 162
Synechococcus_sp._CC9605/1-252 121 159
Synechococcus_sp._CC9616/1-243 121 158
Synechococcus_sp._KORDI-100/1-247 125 162
Synechococcus_sp._KORDI-49/1-240 121 157
Synechococcus_sp._NKBG15041c_concat/1-243 120 154
Synechococcus_sp._PCC_7002/1-293 120 158
Synechococcus_sp._PCC_7335/1-312 119 163
Synechococcus_sp._RCC307/1-247 128 166
Synechococcus_sp._RS9916/1-275 121 159
Synechococcus_sp._WH_5701/1-258 121 159
Synechococcus_sp._WH_7803/1-240 123 161
Synechococcus_sp._WH_7805/1-241 123 161
Synechococcus_sp._WH_8016/1-256 124 168
Synechococcus_sp._WH_8102/1-248 121 159
Synechocystis_sp._PCC_6714_concat/1-255 123 160
Synechocystis_sp._PCC_6803_concat/1-258 119 156
Synechocystis_sp._PCC_7509/1-298 120 158
Thermosynechococcus_elongatus_BP-1_concat/1-269 117 155
Tolypothrix_bouteillei_VB521301/1-303 120 158
Trichodesmium_erythraeum_IMS101_concat/1-247 118 156
Trichormus_azollae/1-302 120 158
Xenococcus_sp._PCC_7305_concat/1-260 126 164
cyanobacterium_PCC_7702/1-302 119 157
cyanobacterium_endosymbiont_of_Epithemia_turgida/1-252 118 156
filamentous_cyanobacterium_ESFC-1/1-291 117 155

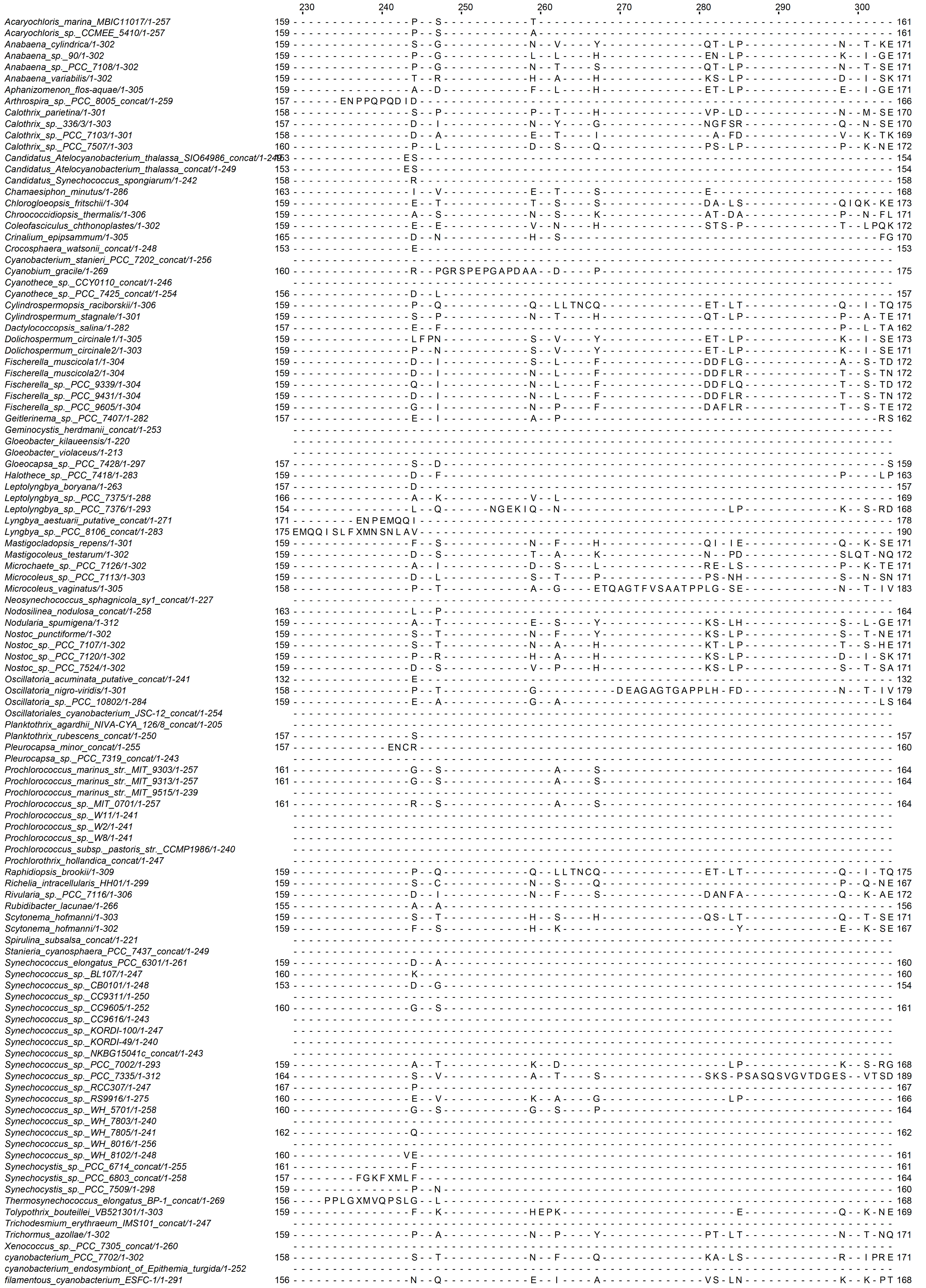

230
240
250
260
270
280
290
300
Acaryochloris_marina_MBIC11017/1-257
Acaryochloris_sp._CCMEE_5410/1-257
Anabaena_cylindrica/1-302
Anabaena_sp._90/1-302
Anabaena_sp._PCC_7108/1-302
Anabaena_variabilis/1-302
Aphanizomenon_flos-aquae/1-305
Arthrospira_sp._PCC_8005_concat/1-259
Calothrix_parietina/1-301
Calothrix_sp._336/3/1-303
Calothrix_sp._PCC_7103/1-301
Calothrix_sp._PCC_7507/1-303
Candidatus_Atelocyanobacterium_thalassa_SIO64986_concat/1-249
Candidatus_Atelocyanobacterium_thalassa_concat/1-249
Candidatus_Synechococcus_spongiarum/1-242
Chamaesiphon_minutus/1-286
Chlorogloeopsis_fritschii/1-304
Chroococcidiopsis_thermalis/1-306
Coleofasciculus_chthonoplastes/1-302
Crinalium_epipsammum/1-305
Crocosphaera_watsonii_concat/1-248
Cyanobacterium_stanieri_PCC_7202_concat/1-256
Cyanobium_gracile/1-269
Cyanothece_sp._CCY0110_concat/1-246
Cyanothece_sp._PCC_7425_concat/1-254
Cylindrospermopsis_raciborskii/1-306
Cylindrospermum_stagnale/1-301
Dactylococcopsis_salina/1-282
Dolichospermum_circinale1/1-305
Dolichospermum_circinale2/1-303
Fischerella_muscicola1/1-304
Fischerella_muscicola2/1-304
Fischerella_sp._PCC_9339/1-304
Fischerella_sp._PCC_9431/1-304
Fischerella_sp._PCC_9605/1-304
Geitlerinema_sp._PCC_7407/1-282
Geminocystis_herdmanii_concat/1-253
Gloeobacter_kilaueensis/1-220
Gloeobacter_violaceus/1-213
Gloeocapsa_sp._PCC_7428/1-297
Halothece_sp._PCC_7418/1-283
Leptolyngbya_boryana/1-263
Leptolyngbya_sp._PCC_7375/1-288
Leptolyngbya_sp._PCC_7376/1-293
Lyngbya_aestuarii_putative_concat/1-271
Lyngbya_sp._PCC_8106_concat/1-283
Mastigocladopsis_repens/1-301
Mastigocoleus_testarum/1-302
Microchaete_sp._PCC_7126/1-302
Microcoleus_sp._PCC_7113/1-303
Microcoleus_vaginatus/1-305
Neosynechococcus_sphagnicola_sy1_concat/1-227
Nodosilinea_nodulosa_concat/1-258
Nodularia_spumigena/1-312
Nostoc_punctiforme/1-302
Nostoc_sp._PCC_7107/1-302
Nostoc_sp._PCC_7120/1-302
Nostoc_sp._PCC_7524/1-302
Oscillatoria_acuminata_putative_concat/1-241
Oscillatoria_nigro-viridis/1-301
Oscillatoria_sp._PCC_10802/1-284
Oscillatoriales_cyanobacterium_JSC-12_concat/1-254
Planktothrix_agardhii_NIVA-CYA_126/8_concat/1-205
Planktothrix_rubescens_concat/1-250
Pleurocapsa_minor_concat/1-255
Pleurocapsa_sp._PCC_7319_concat/1-243
Prochlorococcus_marinus_str._MIT_9303/1-257
Prochlorococcus_marinus_str._MIT_9313/1-257
Prochlorococcus_marinus_str._MIT_9515/1-239
Prochlorococcus_sp._MIT_0701/1-257
Prochlorococcus_sp._W11/1-241
Prochlorococcus_sp._W2/1-241
Prochlorococcus_sp._W8/1-241
Prochlorococcus_subsp._pastoris_str._CCMP1986/1-240
Prochlorothrix_hollandica_concat/1-247
Raphidiopsis_brookii/1-309
Richelia_intracellularis_HH01/1-299
Rivularia_sp._PCC_7116/1-306
Rubidibacter_lacunae/1-266
Scytonema_hofmanni/1-303
Scytonema_hofmanni/1-302
Spirulina_subsalsa_concat/1-221
Stanieria_cyanosphaera_PCC_7437_concat/1-249
Synechococcus_elongatus_PCC_6301/1-261
Synechococcus_sp._BL107/1-247
Synechococcus_sp._CB0101/1-248
Synechococcus_sp._CC9311/1-250
Synechococcus_sp._CC9605/1-252
Synechococcus_sp._CC9616/1-243
Synechococcus_sp._KORDI-100/1-247
Synechococcus_sp._KORDI-49/1-240
Synechococcus_sp._NKBG15041c_concat/1-243
Synechococcus_sp._PCC_7002/1-293
Synechococcus_sp._PCC_7335/1-312
Synechococcus_sp._RCC307/1-247
Synechococcus_sp._RS9916/1-275
Synechococcus_sp._WH_5701/1-258
Synechococcus_sp._WH_7803/1-240
Synechococcus_sp._WH_7805/1-241
Synechococcus_sp._WH_8016/1-256
Synechococcus_sp._WH_8102/1-248
Synechocystis_sp._PCC_6714_concat/1-255
Synechocystis_sp._PCC_6803_concat/1-258
Synechocystis_sp._PCC_7509/1-298
Thermosynechococcus_elongatus_BP-1_concat/1-269
Tolypothrix_bouteillei_VB521301/1-303
Trichodesmium_erythraeum_IMS101_concat/1-247
Trichormus_azollae/1-302
Xenococcus_sp._PCC_7305_concat/1-260
cyanobacterium_PCC_7702/1-302
cyanobacterium_endosymbiont_of_Epithemia_turgida/1-252
filamentous_cyanobacterium_ESFC-1/1-291

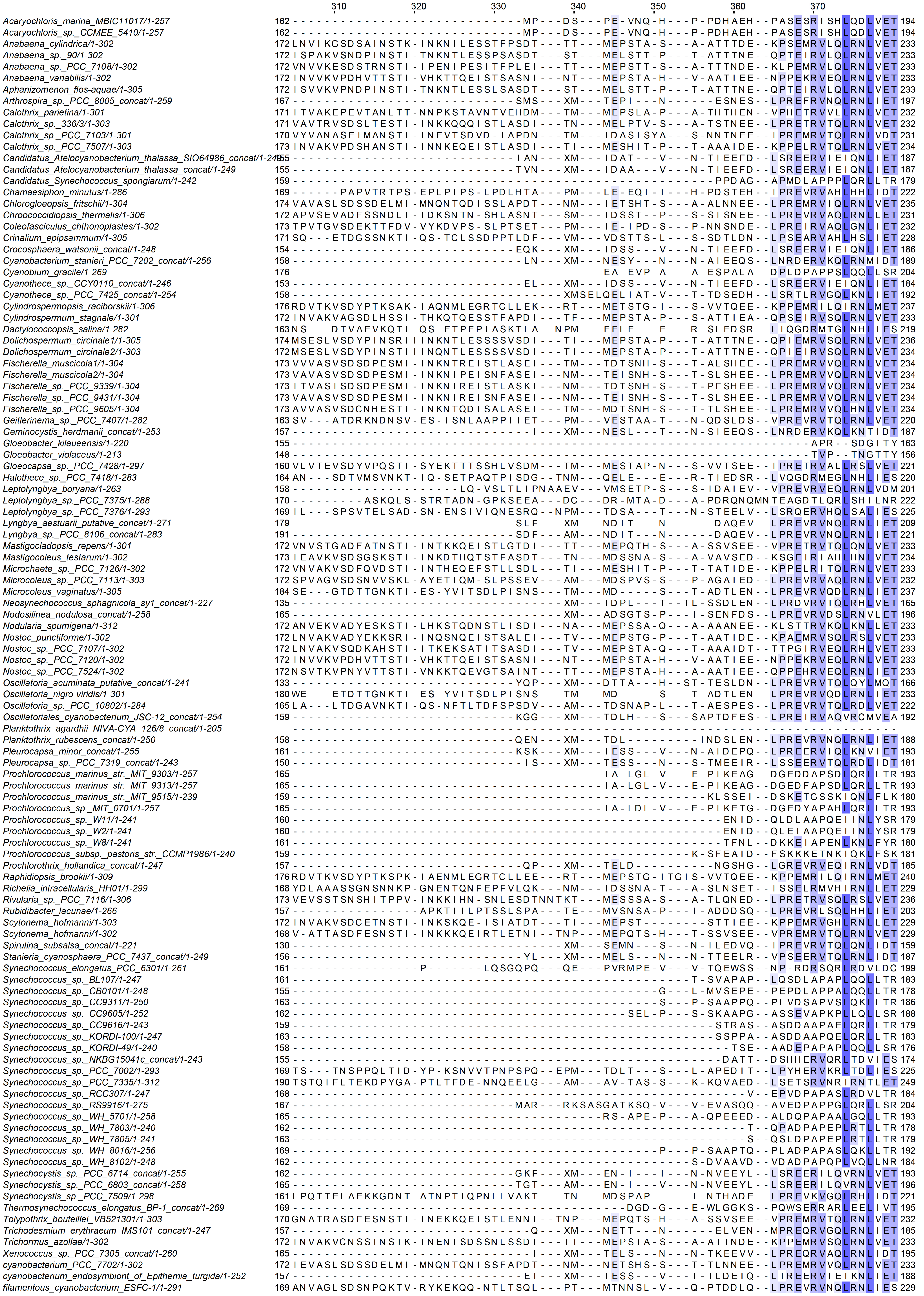
310
320
330
340
350
360
370
Acaryochloris_marina_MBIC11017/1-257
Acaryochloris_sp._CCMEE_5410/1-257
Anabaena_cylindrica/1-302
Anabaena_sp._90/1-302
Anabaena_sp._PCC_7108/1-302
Anabaena_variabilis/1-302
Aphanizomenon_flos-aquae/1-305
Arthrospira_sp._PCC_8005_concat/1-259
Calothrix_parietina/1-301
Calothrix_sp._336/3/1-303
Calothrix_sp._PCC_7103/1-301
Calothrix_sp._PCC_7507/1-303
Candidatus_Atelocyanobacterium_thalassa_SIO64986_concat/1-249
Candidatus_Atelocyanobacterium_thalassa_concat/1-249
Candidatus_Synechococcus_spongiarum/1-242
Chamaesiphon_minutus/1-286
Chlorogloeopsis_fritschii/1-304
Chroococcidiopsis_thermalis/1-306
Coleofasciculus_chthonoplastes/1-302
Crinalium_epipsammum/1-305
Crocosphaera_watsonii_concat/1-248
Cyanobacterium_stanieri_PCC_7202_concat/1-256
Cyanobium_gracile/1-269
Cyanothece_sp._CCY0110_concat/1-246
Cyanothece_sp._PCC_7425_concat/1-254
Cylindrospermopsis_raciborskii/1-306
Cylindrospermum_stagnale/1-301
Dactylococcopsis_salina/1-282
Dolichospermum_circinale1/1-305
Dolichospermum_circinale2/1-303
Fischerella_muscicola1/1-304
Fischerella_muscicola2/1-304
Fischerella_sp._PCC_9339/1-304
Fischerella_sp._PCC_9431/1-304
Fischerella_sp._PCC_9605/1-304
Geitlerinema_sp._PCC_7407/1-282
Geminocystis_herdmanii_concat/1-253
Gloeobacter_kilaueensis/1-220
Gloeobacter_violaceus/1-213
Gloeocapsa_sp._PCC_7428/1-297
Halothece_sp._PCC_7418/1-283
Leptolyngbya_boryana/1-263
Leptolyngbya_sp._PCC_7375/1-288
Leptolyngbya_sp._PCC_7376/1-293
Lyngbya_aestuarii_putative_concat/1-271
Lyngbya_sp._PCC_8106_concat/1-283
Mastigocladopsis_repens/1-301
Mastigocoleus_testarum/1-302
Microchaete_sp._PCC_7126/1-302
Microcoleus_sp._PCC_7113/1-303
Microcoleus_vaginatus/1-305
Neosynechococcus_sphagnicola_sy1_concat/1-227
Nodosilinea_nodulosa_concat/1-258
Nodularia_spumigena/1-312
Nostoc_punctiforme/1-302
Nostoc_sp._PCC_7107/1-302
Nostoc_sp._PCC_7120/1-302
Nostoc_sp._PCC_7524/1-302
Oscillatoria_acuminata_putative_concat/1-241
Oscillatoria_nigro-viridis/1-301
Oscillatoria_sp._PCC_10802/1-284
Oscillatoriales_cyanobacterium_JSC-12_concat/1-254
Planktothrix_agardhii_NIVA-CYA_126/8_concat/1-205
Planktothrix_rubescens_concat/1-250
Pleurocapsa_minor_concat/1-255
Pleurocapsa_sp._PCC_7319_concat/1-243
Prochlorococcus_marinus_str._MIT_9303/1-257
Prochlorococcus_marinus_str._MIT_9313/1-257
Prochlorococcus_marinus_str._MIT_9515/1-239
Prochlorococcus_sp._MIT_0701/1-257
Prochlorococcus_sp._W11/1-241
Prochlorococcus_sp._W2/1-241
Prochlorococcus_sp._W8/1-241
Prochlorococcus_subsp._pastoris_str._CCMP1986/1-240
Prochlorothrix_hollandica_concat/1-247
Raphidiopsis_brookii/1-309
Richelia_intracellularis_HH01/1-299
Rivularia_sp._PCC_7116/1-306
Rubidibacter_lacunae/1-266
Scytonema_hofmanni/1-303
Scytonema_hofmanni/1-302
Spirulina_subsalsa_concat/1-221
Stanieria_cyanosphaera_PCC_7437_concat/1-249
Synechococcus_elongatus_PCC_6301/1-261
Synechococcus_sp._BL107/1-247
Synechococcus_sp._CB0101/1-248
Synechococcus_sp._CC9311/1-250
Synechococcus_sp._CC9605/1-252
Synechococcus_sp._CC9616/1-243
Synechococcus_sp._KORDI-100/1-247
Synechococcus_sp._KORDI-49/1-240
Synechococcus_sp._NKBG15041c_concat/1-243
Synechococcus_sp._PCC_7002/1-293
Synechococcus_sp._PCC_7335/1-312
Synechococcus_sp._RCC307/1-247
Synechococcus_sp._RS9916/1-275
Synechococcus_sp._WH_5701/1-258
Synechococcus_sp._WH_7803/1-240
Synechococcus_sp._WH_7805/1-241
Synechococcus_sp._WH_8016/1-256
Synechococcus_sp._WH_8102/1-248
Synechocystis_sp._PCC_6714_concat/1-255
Synechocystis_sp._PCC_6803_concat/1-258
Synechocystis_sp._PCC_7509/1-298
Thermosynechococcus_elongatus_BP-1_concat/1-269
Tolypothrix_bouteillei_VB521301/1-303
Trichodesmium_erythraeum_IMS101_concat/1-247
Trichormus_azollae/1-302
Xenococcus_sp._PCC_7305_concat/1-260
cyanobacterium_PCC_7702/1-302
cyanobacterium_endosymbiont_of_Epithemia_turgida/1-252
filamentous_cyanobacterium_ESFC-1/1-291

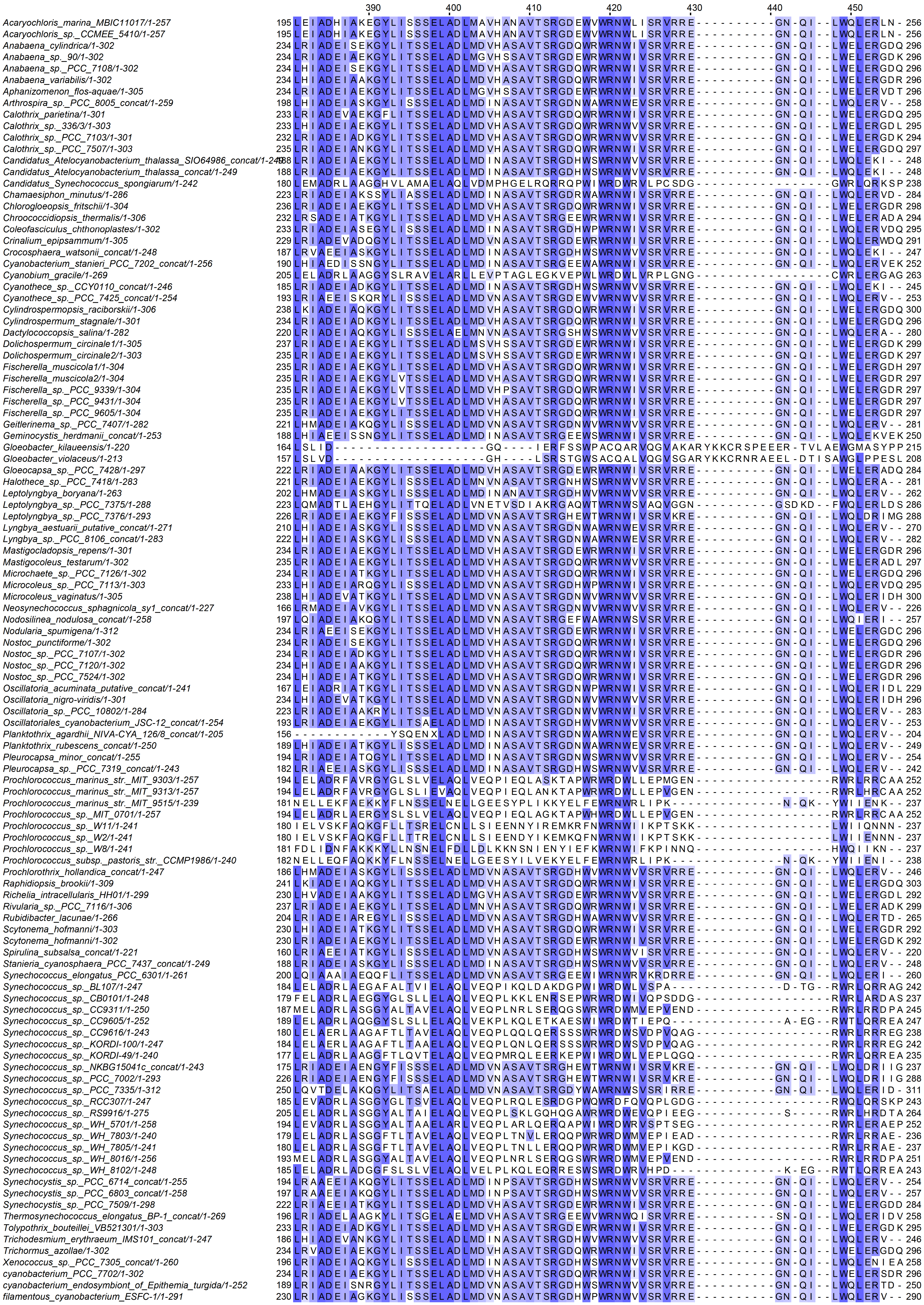
390
400
410
420
430
440
450
Acaryochloris_marina_MBIC11017/1-257
Acaryochloris_sp._CCMEE_5410/1-257
Anabaena_cylindrica/1-302
Anabaena_sp._90/1-302
Anabaena_sp._PCC_7108/1-302
Anabaena_variabilis/1-302
Aphanizomenon_flos-aquae/1-305
Arthrospira_sp._PCC_8005_concat/1-259
Calothrix_parietina/1-301
Calothrix_sp._336/3/1-303
Calothrix_sp._PCC_7103/1-301
Calothrix_sp._PCC_7507/1-303
Candidatus_Atelocyanobacterium_thalassa_SIO64986_concat/1-249
Candidatus_Atelocyanobacterium_thalassa_concat/1-249
Candidatus_Synechococcus_spongiarum/1-242
Chamaesiphon_minutus/1-286
Chlorogloeopsis_fritschii/1-304
Chroococcidiopsis_thermalis/1-306
Coleofasciculus_chthonoplastes/1-302
Crinalium_epipsammum/1-305
Crocosphaera_watsonii_concat/1-248
Cyanobacterium_stanieri_PCC_7202_concat/1-256
Cyanobium_gracile/1-269
Cyanothece_sp._CCY0110_concat/1-246
Cyanothece_sp._PCC_7425_concat/1-254
Cylindrospermopsis_raciborskii/1-306
Cylindrospermum_stagnale/1-301
Dactylococcopsis_salina/1-282
Dolichospermum_circinale1/1-305
Dolichospermum_circinale2/1-303
Fischerella_muscicola1/1-304
Fischerella_muscicola2/1-304
Fischerella_sp._PCC_9339/1-304
Fischerella_sp._PCC_9431/1-304
Fischerella_sp._PCC_9605/1-304
Geitlerinema_sp._PCC_7407/1-282
Geminocystis_herdmanii_concat/1-253
Gloeobacter_kilaueensis/1-220
Gloeobacter_violaceus/1-213
Gloeocapsa_sp._PCC_7428/1-297
Halothece_sp._PCC_7418/1-283
Leptolyngbya_boryana/1-263
Leptolyngbya_sp._PCC_7375/1-288
Leptolyngbya_sp._PCC_7376/1-293
Lyngbya_aestuarii_putative_concat/1-271
Lyngbya_sp._PCC_8106_concat/1-283
Mastigocladopsis_repens/1-301
Mastigocoleus_testarum/1-302
Microchaete_sp._PCC_7126/1-302
Microcoleus_sp._PCC_7113/1-303
Microcoleus_vaginatus/1-305
Neosynechococcus_sphagnicola_sy1_concat/1-227
Nodosilinea_nodulosa_concat/1-258
Nodularia_spumigena/1-312
Nostoc_punctiforme/1-302
Nostoc_sp._PCC_7107/1-302
Nostoc_sp._PCC_7120/1-302
Nostoc_sp._PCC_7524/1-302
Oscillatoria_acuminata_putative_concat/1-241
Oscillatoria_nigro-viridis/1-301
Oscillatoria_sp._PCC_10802/1-284
Oscillatoriales_cyanobacterium_JSC-12_concat/1-254
Planktothrix_agardhii_NIVA-CYA_126/8_concat/1-205
Planktothrix_rubescens_concat/1-250
Pleurocapsa_minor_concat/1-255
Pleurocapsa_sp._PCC_7319_concat/1-243
Prochlorococcus_marinus_str._MIT_9303/1-257
Prochlorococcus_marinus_str._MIT_9313/1-257
Prochlorococcus_marinus_str._MIT_9515/1-239
Prochlorococcus_sp._MIT_0701/1-257
Prochlorococcus_sp._W11/1-241
Prochlorococcus_sp._W2/1-241
Prochlorococcus_sp._W8/1-241
Prochlorococcus_subsp._pastoris_str._CCMP1986/1-240
Prochlorothrix_hollandica_concat/1-247
Raphidiopsis_brookii/1-309
Richelia_intracellularis_HH01/1-299
Rivularia_sp._PCC_7116/1-306
Rubidibacter_lacunae/1-266
Scytonema_hofmanni/1-303
Scytonema_hofmanni/1-302
Spirulina_subsalsa_concat/1-221
Stanieria_cyanosphaera_PCC_7437_concat/1-249
Synechococcus_elongatus_PCC_6301/1-261
Synechococcus_sp._BL107/1-247
Synechococcus_sp._CB0101/1-248
Synechococcus_sp._CC9311/1-250
Synechococcus_sp._CC9605/1-252
Synechococcus_sp._CC9616/1-243
Synechococcus_sp._KORDI-100/1-247
Synechococcus_sp._KORDI-49/1-240
Synechococcus_sp._NKBG15041c_concat/1-243
Synechococcus_sp._PCC_7002/1-293
Synechococcus_sp._PCC_7335/1-312
Synechococcus_sp._RCC307/1-247
Synechococcus_sp._RS9916/1-275
Synechococcus_sp._WH_5701/1-258
Synechococcus_sp._WH_7803/1-240
Synechococcus_sp._WH_7805/1-241
Synechococcus_sp._WH_8016/1-256
Synechococcus_sp._WH_8102/1-248
Synechocystis_sp._PCC_6714_concat/1-255
Synechocystis_sp._PCC_6803_concat/1-258
Synechocystis_sp._PCC_7509/1-298
Thermosynechococcus_elongatus_BP-1_concat/1-269
Tolypothrix_bouteillei_VB521301/1-303
Trichodesmium_erythraeum_IMS101_concat/1-247
Trichormus_azollae/1-302
Xenococcus_sp._PCC_7305_concat/1-260
cyanobacterium_PCC_7702/1-302
cyanobacterium_endosymbiont_of_Epithemia_turgida/1-252
filamentous_cyanobacterium_ESFC-1/1-291

	Start	460 470 480	End
Acaryochloris_marina_MBIC11017/1-257		----------------------------------S	257
Acaryochloris_sp._CCMEE_5410/1-257		----------------------------------S	257
Anabaena_cylindrica/1-302	297	---------L--E-----S-------------EAD	302
Anabaena_sp._90/1-302	297	---------I--A-----T-------------EID	302
Anabaena_sp._PCC_7108/1-302	297	---------V--E-----T-------------DTD	302
Anabaena_variabilis/1-302	297	---------L--G-----G-------------EDE	302
Aphanizomenon_flos-aquae/1-305	297	---------L--RS--KDT-------------QIL	305
Arthrospira_sp._PCC_8005_concat/1-259		----------------------------------D	259
Calothrix_parietina/1-301	296	---------V--E-----S-------------EEE	301
Calothrix_sp._336/3/1-303	296	---------V--NH---EG-------------EES	303
Calothrix_sp._PCC_7103/1-301	295	---------I--Q-----EE------------SNE	301
Calothrix_sp._PCC_7507/1-303	298	---------V--G-----G-------------ESE	303
Candidatus_Atelocyanobacterium_thalassa_SIO64986_concat/1-249		----------------------------------D	249
Candidatus_Atelocyanobacterium_thalassa_concat/1-249		----------------------------------D	249
Candidatus_Synechococcus_spongiarum/1-242	239	------------------D-------------PHH	242
Chamaesiphon_minutus/1-286	285	---------------------------------GE	286
Chlorogloeopsis_fritschii/1-304	299	---------I--N-----E-------------GDE	304
Chroococcidiopsis_thermalis/1-306	295	---------I--N-----PD--DNSDH-----ATE	306
Coleofasciculus_chthonoplastes/1-302	296	---------V--KS----G-------------DEE	302
Crinalium_epipsammum/1-305	292	---------T--N-----SE-----SEHNSDLATE	305
Crocosphaera_watsonii_concat/1-248		----------------------------------D	248
Cyanobacterium_stanieri_PCC_7202_concat/1-256	253	---------I----------------------MDD	256
Cyanobium_gracile/1-269	264	---------G--L-----D-------------RPQ	269
Cyanothece_sp._CCY0110_concat/1-246		----------------------------------D	246
Cyanothece_sp._PCC_7425_concat/1-254		----------------------------------D	254
Cylindrospermopsis_raciborskii/1-306	301	---------V--E-----K-------------DDN	306
Cylindrospermum_stagnale/1-301	297	---------V--E-------------------SEE	301
Dactylococcopsis_salina/1-282	281	---------------------------------EG	282
Dolichospermum_circinale1/1-305	300	---------I--EI------------------EVE	305
Dolichospermum_circinale2/1-303	298	---------I--ET------------------EIE	303
Fischerella_muscicola1/1-304	298	---------L--ND----E-------------NDS	304
Fischerella_muscicola2/1-304	298	---------L--ND----E-------------NDD	304
Fischerella_sp._PCC_9339/1-304	298	---------L--ND----E-------------NDD	304
Fischerella_sp._PCC_9431/1-304	298	---------L--ND----E-------------NDD	304
Fischerella_sp._PCC_9605/1-304	298	---------I--KD----E-------------NDE	304
Geitlerinema_sp._PCC_7407/1-282		----------------------------------D	282
Geminocystis_herdmanii_concat/1-253	251	---------V-----------------------AE	253
Gloeobacter_kilaueensis/1-220	216	---------E--------A-------------LKS	220
Gloeobacter_violaceus/1-213	209	---------V--------Q-------------LEG	213
Gloeocapsa_sp._PCC_7428/1-297	285	---------V--N-----SD-DHTGDP-----ATE	297
Halothece_sp._PCC_7418/1-283	282	---------------------------------EG	283
Leptolyngbya_boryana/1-263		----------------------------------D	263
Leptolyngbya_sp._PCC_7375/1-288	287	---------------------------------LY	288
Leptolyngbya_sp._PCC_7376/1-293	289	---------D--A-------------------EAS	293
Lyngbya_aestuarii_putative_concat/1-271		----------------------------------D	271
Lyngbya_sp._PCC_8106_concat/1-283		----------------------------------D	283
Mastigocladopsis_repens/1-301	297	---------V--E-------------------NEE	301
Mastigocoleus_testarum/1-302	298	---------V--N-------------------DED	302
Microchaete_sp._PCC_7126/1-302	297	---------I--G-----G-------------EAE	302
Microcoleus_sp._PCC_7113/1-303	296	---------V--N-----TA------D-----DEG	303
Microcoleus_vaginatus/1-305	301	---------V--S-------------------TTD	305
Neosynechococcus_sphagnicola_sy1_concat/1-227		----------------------------------D	227
Nodosilinea_nodulosa_concat/1-258		----------------------------------D	258
Nodularia_spumigena/1-312	297	GSRSAGGDRLETE-------------------DEI	312
Nostoc_punctiforme/1-302	297	---------V--G-----G-------------EEE	302
Nostoc_sp._PCC_7107/1-302	297	---------V--G-----G-------------EEE	302
Nostoc_sp._PCC_7120/1-302	297	---------L--G-----G-------------EDE	302
Nostoc_sp._PCC_7524/1-302	297	---------L--G-----G-------------EED	302
Oscillatoria_acuminata_putative_concat/1-241	230	---------F--N-----GS--GEDDG-----AGE	241
Oscillatoria_nigro-viridis/1-301	297	---------V--S-------------------TTD	301
Oscillatoria_sp._PCC_10802/1-284		----------------------------------D	284
Oscillatoriales_cyanobacterium_JSC-12_concat/1-254		----------------------------------D	254
Planktothrix_agardhii_NIVA-CYA_126/8_concat/1-205		----------------------------------N	205
Planktothrix_rubescens_concat/1-250		----------------------------------N	250
Pleurocapsa_minor_concat/1-255		----------------------------------D	255
Pleurocapsa_sp._PCC_7319_concat/1-243		----------------------------------D	243
Prochlorococcus_marinus_str._MIT_9303/1-257	253	---------G--------S-------------DQI	257
Prochlorococcus_marinus_str._MIT_9313/1-257	253	---------G--------S-------------DQI	257
Prochlorococcus_marinus_str._MIT_9515/1-239	238	---------------------------------HA	239
Prochlorococcus_sp._MIT_0701/1-257	253	---------G--------S-------------DQI	257
Prochlorococcus_sp._W11/1-241	238	------------------Q-------------NPQ	241
Prochlorococcus_sp._W2/1-241	238	------------------Q-------------NPQ	241
Prochlorococcus_sp._W8/1-241	238	------------------K-------------DIE	241
Prochlorococcus_subsp._pastoris_str._CCMP1986/1-240	239	---------------------------------NT	240
Prochlorothrix_hollandica_concat/1-247		----------------------------------D	247
Raphidiopsis_brookii/1-309	304	---------V--E-----K-------------DDN	309
Richelia_intracellularis_HH01/1-299	293	---------F--D-----EE------------DEN	299
Rivularia_sp._PCC_7116/1-306	300	---------L--DD----D-------------EGE	306
Rubidibacter_lacunae/1-266		----------------------------------C	266
Scytonema_hofmanni/1-303	293	---------V--G-----SE------D--DAAIGG	303
Scytonema_hofmanni/1-302	293	---------V--ES----E-------E---EGSRE	302
Spirulina_subsalsa_concat/1-221		----------------------------------D	221
Stanieria_cyanosphaera_PCC_7437_concat/1-249		----------------------------------D	249
Synechococcus_elongatus_PCC_6301/1-261		----------------------------------H	261
Synechococcus_sp._BL107/1-247	243	---------G--S-------------------GES	247
Synechococcus_sp._CB0101/1-248	238	---------G--SGVTEQQ-------------RDG	248
Synechococcus_sp._CC9311/1-250	246	---------G--------S-------------TET	250
Synechococcus_sp._CC9605/1-252	248	---------G--S-------------------EQS	252
Synechococcus_sp._CC9616/1-243	239	---------G--------S-------------EQS	243
Synechococcus_sp._KORDI-100/1-247	243	---------G--------S-------------EQS	247
Synechococcus_sp._KORDI-49/1-240	236	---------G--------S-------------GEP	240
Synechococcus_sp._NKBG15041c_concat/1-243	238	---------G--DE------------------TTA	243
Synechococcus_sp._PCC_7002/1-293	289	---------D--E-------------------TTT	293
Synechococcus_sp._PCC_7335/1-312		----------------------------------E	312
Synechococcus_sp._RCC307/1-247	244	------------------E-------------ATP	247
Synechococcus_sp._RS9916/1-275	265	---------G--SKQNDEA-------------GHG	275
Synechococcus_sp._WH_5701/1-258	253	---------V--L-----Q-------------EFP	258
Synechococcus_sp._WH_7803/1-240	237	------------------A-------------GSR	240
Synechococcus_sp._WH_7805/1-241	238	------------------A-------------GSR	241
Synechococcus_sp._WH_8016/1-256	252	---------G--------L-------------KQT	256
Synechococcus_sp._WH_8102/1-248	244	---------G--S-------------------EQS	248
Synechocystis_sp._PCC_6714_concat/1-255		----------------------------------D	255
Synechocystis_sp._PCC_6803_concat/1-258		----------------------------------D	258
Synechocystis_sp._PCC_7509/1-298	285	---------V--S-----SATSYNSDP-----ATE	298
Thermosynechococcus_elongatus_BP-1_concat/1-269	259	---------A--KE----Q----SSEL-----PPT	269
Tolypothrix_bouteillei_VB521301/1-303	296	---------V--AS----E-------E-----EGD	303
Trichodesmium_erythraeum_IMS101_concat/1-247		----------------------------------D	247
Trichormus_azollae/1-302	297	---------I--G-----T-------------EAE	302
Xenococcus_sp._PCC_7305_concat/1-260	259	---------------------------------VE	260
cyanobacterium_PCC_7702/1-302	297	---------I--K-----D-------------EDE	302
cyanobacterium_endosymbiont_of_Epithemia_turgida/1-252	251	---------------------------------LE	252
filamentous_cyanobacterium_ESFC-1/1-291		----------------------------------D	291

Supplementary Alignment 2

Aligned homologs of *Trichodesmium erythraeum* RNase HI.
These alignments were were done with ProbCons (see Methods) and used to build the Bayesian tree shown in Figure 2A.

```
>Synechococcus_sp_NKBG15041c
AT-GGC-ACA-AC-A-ACAATTGATTG--ACTGTAT-TTACACTGATGGTGGCTGCAT-C
CATAACCCTGGCCCCGGCGGGTGGGGTGTGGTTGTCCA--ATTTACC-GATGGTT-----
------CT--GTGCTTGAAATGGGCGATCACCAACCAGAGACCACCAACAATCGCATGGA
AATGC-AAGCTGCGATCCAAGCCCTTGAGTATGTTCGTGA----ATGCGATCAACAG---
------GAGC-CCATGCGTCTCTTAACGGACAGTAAATATGTGATCGATGGGATCACGAA
---G---TGGATCAAAGGCTGGCAAAAAAAAGGCTGGAAAACCGCCGCCGGCAAACCAGT
CCTCAACCAAGACCTCTGGCAACGACTCCATCAGCT-CA-----A-T-CATCC-------
---------------CCAAATCTCCTGGTGCCATGTCAAGGGCCACAGCGGTGAAC-CAG
GCAACGA--GCGGGCCGATGCGATCG---CCAA-TGGTTT---TGC-CCGGGG-------
-----------CC---AA---AAG----CC---C-C--ACCTC---------------C-
----GGAAC------A--------------------------------------------
----------ACCAA--AGC---------------------------------GCCTCCA
TGCCGAAAAAA-TC-TACC--AAAAT---C-A-A--GAGGTAA
>Synechococcus_sp_PCC_7002
AT-GGC-ACA-AC-A-ACGCACAATTG--ACCGCAT-TTATACCGACGGTGGCTGTAT-T
CGCAATCCCGGCCCCGGTGGTTGGGGAGTGGTGATCCA--GTTCACC-GATGGCA-----
------CC--GTTTTGGAAATGGGCGATCGCCAACCCGAAACCACCAATAACCGCATGGA
AATGC-AAGCCGCAATCCAGGCCCTCGAATTTGTCCGCGA----CTGTCCCCAGCCA---
------GAAC-CAGTGCGCCTTTTTACCGATAGTAAATATGTCATCGACGGCATCACTAA
---A---TGGATTAAAGGTTGGCAAAAAAAGGGCTGGAAAACCGCCACTGGCAACCCCGT
TTTAAATCAAGACCTCTGGCAGCAGCTCAATCAACT-GA-----A-T-CAACC-------
---------------GACCATCTCTTGGTGTCACGTCAAAGGCCATAGCGGTGAGG-CGG
GGAATGA--ACGGGCCGATGCGATCG---CCAA-TGGCTT---TGC-CCGGGG-------
-----------TC---AA---GCT----CC---T-A--ACCTA---------------C-
-------GC--AG--C--------------------------------------------
----------CCCAA--TGC-----------------------------------A----
-ACC--AAAGAT-C-TACC--AAAAT---C-T-A--GAGGTAC
>Acaryochloris_marina_MBIC11017
AT-GCC-GGC-TT-T-TTCTGATATTG--ACAGTAT-CTATACTGATGGCGCTTGTTC-A
GGGAATCCTGGTCCAGGCGGATGGGGCAGTGTCATCTA--CTTCAAA-GATGGAT-----
------CC--TGCCATGAAATTGGCGGGTTTGTGCCTCAAACGACGAATAACCGGATGGA
ATTGC-AGGCTGCGATCGCAATCTTGGAATTGTTGCAAGA----CCAACCGTTACCT---
------AAGA-CCATTCCCCTCTATACTGACAGCGAATACGTCAAAAATGGCATTACTAA
---G---TGGTTGGCAGGATGGAAACGTAAAGGCTGGAAAACAGCTAAAGGTAAAGCGGT
TCTGAATCAAGATTTGTGGCAACAGCTCGATCAACT-TA-----A-T-GATGC-------
---------------GACCATAGACTGGCGATATATCCGCGCTCATGGAGGCCATG-AAG
GCAATGA--GCGATGTGATCAGATCG---CTCG-TGCTTT---TTC-CCAGTA-------
-----------TC---AA---ACC----CC---T-G--ACCTT---------------C-
------------------------------------------------------------
----------ACCAAATTAC-----------------------------------C----
-CCC--AG-TACCA-TGCC------------------AGACTC
>Acaryochloris_sp_CCMEE_5410
AT-GCC-GGC-TT-T-TTCTGATATTG--ACAGTAT-CTATACTGATGGCGCTTGCTC-G
GGGAATCCCGGTCCAGGCGGATGGGGCAGTGTCATCTA--CTTCAAG-GATGGAT-----
------CC--TGCCATGAAATTGGCGGGTTTGTGCCTCAAACGACGAATAACCGGATGGA
```

ATTGC-AGGCTGCGATCGCAATCTTGGAATTGTTGCAAGA----CCAACCGTTACCC---
------AAGA-CCATTCCCCTCTATACTGACAGCGAGTACGTCAAAAATGGCATTACTAA
---G---TGGTTGGCAGGATGGAAACGCAAAGGCTGGAAAACAGCTAAAGGTAAAGCGGT
TCTGAATCAAGATTTGTGGCAACAGCTCGATCAACT-TA-----A-T-GATGC-------
---------------GACCATAGACTGGCGATATATCCGCGCTCATGCAGGCCATG-AAG
GCAATGA--GCGATGTGATCAGATCG---CCCG-TGCTTT---TTC-CCAGCA-------
-----------TC---AA---ACC----CC---T-G--ACCTT---------------C-

----------ACCAAATTAC-----------------------------------C----
-CCC--AG-TGCCA-TGCC------------------AGACTC
>Thermosynechococcus_elongatus_BP-1
AT-GAC-CCA-TA-----------TTC--GTGCCAT-CTATACCGATGGTGCCTGTGA-A
GGCAATCCCGGACCGGGGGGGTGGGGCGTGGTTATTTA--CTTTACC-GATGGCT-----
------CT--GTGCACGAGCTCGGGGGACACCACCCGGCCACGACGAATAATCGCATGGA
ATTAC-AGGCTGCGATTGAAGCTCTCAAGGCATGGCGCCA----AC---TGGCCCCT---
----GGGAGCGCGATCGCCCTTTACACCGACAGCGAGTATGTCCTCAGGGGAATTACTGA
---G---TGGATTCACCACTGGAAACGGCGGGGCTGGAAAACAGCCGCCAAAAAGCCGGT
TCTCAACCAAGACCTCTGGCAGGAGTTAGATGCCCT-CA-----A-T-GATCC-------
---------------CTTAGTCCAATGGCACCATGTCCGCGGCCATAGGGGAGATG-TCG
GCAATGA--ACGCTGCGATCTTATTG---CCCG-CCG-TT---TGAGTCGTGG-------
-----------GC---A----GCC---------------------------------AA-
---TT-GCC--CT--A--
----------AGAACGCTGC-----------------------------------C----
-ACC------ACC---GC---------------T--GGGGTAA
>Synechococcus_elongatus_PCC_6301
AT-GTC-TGA-TG-C-------CATCC--TCCGCCT-TTACACCGATGGCGCTTGTAC-G
GGCAATCCAGGGCCGGGCGGTTGGGGGGTGCAAATTCA--ATTTGCC-GATGGCA-----
------CG--GTGCAAGAGCTGGGTGGGCGTGACGCTGCCACAACTAATAACCGGATGGA
GTTGC-AGGCTGCGATCGCGGCCTTAGAGTTCTGGCGCGA----TCACTCTGACC-A---
----G-CAAC-CCGTCACCCTCTACACCGACAGCGAATACGTGATCAAAGGGATCACCCA
---A---TGGCTGAAGGGGTGGCAGCGCAAGGGTTGGAAAACGGCGGCGGGTAAGCCGGT
GCTCAACCAGGACTTATGGATGCACTTGGATGACCT-CA-----A-T-TCACC-------
---------------CGCAGCGCAATGGCAATATGTGCGCGGCCATAGCGGCGATC-CGG
GCAATGA--GCGTTGCGATCAAATTG---CCCG-TAGTTT---TGC-TCAGCA-------
-----------AC---G----TCCGTTACC---------ACTCTGCCAACGGTCTGATG-
---CA-CCA--CT--A--
----------CAATCGGGGC--
--------AACCGCA-GCA--GGAGC---CCG-T--TCGGATG
>Trichodesmium_erythraeum_IMS101
AT-GAC-AGA-AA-A-AAGAACCGAAA--TTACTAT-CTATACTGATGGTGCTTGTAG-T
GGAAATCCCGGCCCTGGTGGTTATGGTATAATTATTTT--ATCTGAG-AAA--AA-----
------AC--G-CCAAGAATTATCTGGAGGTTATAAATTAACCACTAATAATCGTATGGA
ATTAA-TGGCAGTAATAGTAGGATTAGAACAACTAGAAAT----T--------CCAT---
-------CAA-TTGTCAATTTATATACTGACTCTAAATATATAGTTGATGCTGTTACAAA
---AGGTTGGGCAAAACGTTGGCGAGCTAACAGTTGGAAACGGAATAAAAAAGATAAAGC
AATGAACCCCGATCTATGGGGAAAATTATTAGATTT-ATGC-A-G-T-AAACA-------
---------------TCAAGTTGAATTTTCTTGGGTACGGGGTCATTCTGGAAATA-TAG
AAAATGA--ACGCTGTGATAAATTAG---CGGT-CAAAGC---TTC-TCA----------
-----------------A---AAA----CT---T-G--ATTTA---------------C-
--------C--CT--C--
----------AG----ACC----------------------------T------------
-----------------T------GG---T-T-A--TCAATAA
>Planktothrix_NIVA-CYA_98
AT-GAA-CGA-CA-A-TTTAAAAAAAG--TTACAAT-TTATACCGATGGAGCTTGTAG-T
GGGAATCCAGGGCCAGGAGGATATGGCGTGGTGTTGAT--GTTTGGC-CCT--CA-----

```
------CC--G-CAAGGAATTATCAGGGGGATATCAACTTACTACTAATAATCGCATGGA
ATTAA-TCGCGGCTATTATAGGATTAGAAGCCTTAAAGTT----T--------CCTT---
-------GTG-CTGTTAATTTATATACGGATTCTCGCTATTTAGTTGATGCCATGAATAA
---AGGATGGGCGAAGAAATGGCAGAAAAATAATTGGCAACGTAGTCAAAAAGAATCGGC
TAAAAATCCTGATTTGTGGGAAAGATTACTCACTAT-ATCC-G-A-A-CAACA-------
---------------TCAAGTTCAATTTCTTTGGGTTAAAGGTCATTCGGGAGATG-AAG
AAAATGA--ATGTTGCGATCGCTTGG---CTGT-CAAGGC---TTC-ACA----------
-----------------T---CAA----CC---G-G--ATTTA---------------C-
--------T--CC--C--------------------------------------------
----------AG----ATTTGG------------GT-------TATTC------------
-----------------C------CA---A-G-A--GAATTAA
>Cyanobacterium_sp_UCYN-A2
TT-GAA-AAA---------------AT--TAAAAAT-TTACACTGATGGTGCCTGTTC-T
GGTAACCCTGGCAAAGGGGGTTATGGAACAATATTAGT--TTTTAAT-AAG--TA-----
------TC--G-GAAAGAATTGTCAGGAGGTTATAGACTAACAACTAATAATCGTATGGA
GATCA-TATCTATTATCATTGGCTTAGAATCTTTAATAGA----A--------TCTT---
-------GCA-ATGTAAGTATTTATTCTGATTCTCGGTATGTAGTAGACAGTATGAATAG
---AGGATGGGCAAGTAAGTGGCAGTCTAACGACTGGAAACGTAACAAACAAGAAATAGC
TAAAAATTCTGATCTATGGAAAAGATTATTAGATTT-ATGC-A-C-T-AAGCA-------
---------------TAATGTCCAGTTTATATGGGTTAAAGGACATGCTGGACATC-CTG
AAAACGA--ACGTTGCGATCAATTAG---CTGT-TCTTGC---CTC-TAA----------
-----------------A---AAA----AT---G-G--AATTA---------------T-
--------T--TA--T--------------------------------------------
----------AG----ACAAAG------------TT-------TTT-G------------
-----------------AA--AGTAT---A-G-C--TAATTGA
>Cyanobacterium_UCYN-A
TT-GAA-AAA---------------AT--TAAAAAT-TTATACTGATGGTGCTTGCTC-T
GGAAATCCAGGTAAAGGAGGTTATGGAGTAATATTAGT--GTTTAAC-AAA--TA-----
------TC--G-AAAAGAATTATCAGGAGGCTATAGACTAACAACTAATAATCGTATGGA
AATAA-TATCGATTATTATTGGGTTAGAGGCTTTAACAAA----A--------TTGT---
-------GTA-ATGTAACTATTTATTCTGACTCTCGTTACGTAGTAGACACTATGAATAA
---AGGATGGGCAAGTAAATGGCAATCTAATAATTGGAAACGCAATAAACAAGAAATAGC
CAAGAATTCTGATCTATGGAAAAGGTTGTTAGATCT-ATGC-A-C-T-AAACA-------
---------------TAATGTTCAATTTATTTGGGTCAAAGGGCATTCTGGACATC-CTG
AGAACGA--ACGATGCGACCAATTAG---CTGT-CCTTGC---TTC-TAA----------
-----------------A---AGA----AT---G-G--AATTA---------------T-
--------T--CA--T--------------------------------------------
----------AG----ACAAGG------------TT-------TTT-G------------
-----------------AA--AGTAC---A-G-T--TAACTAA
>Crocosphaera_watsonii_WH_8501
AT-GAA-CAA---------------AG--TACAAAT-TTATACCGACGGGGCTTGTTC-T
GGCAACCCAGGGAAAGGCGGTTACGGGATTATTTTAGC--CTACAAT-GAA--CA-----
------CC--G-AAAAGAACTCTCCGGCGGTTATCGTTTAACCACTAATAACCGTATGGA
AATGA-TGGCCGCTATTATTGCCCTCGAAGCCCTAAATAA----A--------CCCT---
-------GTG-ATGTAATTTTATATACTGATTCCCGTTATGTAGTCGATGCTATTACCAA
---AGGATGGGCTAAAAAATGGCAAGCCAATGATTGGCAAAGAAACAAAAAAGAACAAGC
CAAGAATCCTGATTTATGGCAAAGATTATTAGACCT-ATGT-G-A-A-CAACA-------
---------------TCAAGTAGAATTTGTCTGGGTTAAAGGTCACGCCGGACACC-CCG
AAAATGA--ACAATGCGATCGCTTAG---CAGT-GGCCGC---TTG-TCA----------
-----------------A---GAA----GT---A-G--AATTA---------------T-
--------C--CA--T--------------------------------------------
----------TG----ATGCAG------------TT-------TAT-G------------
-----------------AA--G-------A-GCA--AAAGTAA
>Cyanothece_sp_CCY_0110
AT-GAA-AAA---------------AG--TACAAAT-TTACACAGATGGGGCTTGTTC-T
```

```
GGTAATCCCGGAAAAGGGGGTTATGGCATCATTTTAGT--CTATAAC-GAA--CA-----
------CC--G-AAAAGAACTATCAGGAGGGTATCGCTTAACCACTAATAATCGCATGGA
AATGA-TGGCCGCTATCATTGGTTTAGAAGCTTTAAAAAC----C--------CCCT---
-------GTG-AGGTGACATTATACACAGACTCTCGTTACTTAGTTGATGCTATCACTAA
---AGGATGGGCAAAAAAATGGCAAGCCAACGGTTGGAAAAGAAATAACAAAGAAGCTGC
AAAAAATCCTGATTTATGGCAAAAACTGTTAGATTT-GTGT-A-A-A-AAACA-------
---------------TGAAGTAAAATTTGTCTGGGTAAAAGGTCACGCAGGCCACC-CAG
AAAATGA--ACAATGCGATCGCTTAG---CTGT-CACAGC---AAC-CCA----------
-----------------A---CAA----TT---G-A--CACTT---------------G-
--------C--CA--T---------------------------------------------
----------TG----ATGAAG------------TG-------TAT-G------------
---------------------------------A--ACTTTGA
>Arthrospira_sp_PCC_8005
AT-GT-------------------------------------------------------
------------------------------------------GTGGT-AGT--CA-----
------CC--G-GAAGGAAATATCGGGAGGCTTCCGACTAACTACTAATAACCGCATGGA
AATGA-TGGCAGCGATCGCCGCCTTACGAGCCCTCAAATT----T--------CCCT---
-------GTT-CTGTGACCCTCTATAGTGATTCTAAATATCTCGTAGATGCTATGACCCT
---GGGATGGGCTAAACGTTGGCAAAAGAATGGATGGCGACGTAATCAGAAGGAATGGGC
GAAAAATCCCGATTTGTGGGCGCAATTATTAGGTTT-GTGC-G-A-G-GAACA-------
---------------TCAAGTCCGGTTTGTCTGGGTTAAGGGTCATGCAGGCGATC-GCG
AAAATGA--AATTTGCGATCGTCTTG---CTGT-AGAAGC---TAC-TCA----------
-----------------T---AGA----GA---T-A--GTTTA---------------C-
--------C--GC--C---------------------------------------------
----------AG----ATGCAG------------GA-------TAC-GAAAACCCACCCC
AACC----TCAAGA-CATC--GAT-T---C-TAT--GAGTTAA
>Lyngbya_aestuarii_BL_J
AA-AAT-TCATCAGA-ACTTCAAGAAG--TAATGTT-ATATACCGATGGGGCTTGTCA-G
GGAAATCCGGGGCCAGGGGGTTATGGAATTGTATTAAT--TCGTGGA-GAT--CA-----
------TC--G-AGAAGAATTATCAGGAGGTTTTCAGTTCACCACCAATAATCGCATGGA
AATGA-TGGCGGCAATTGTGGGGTTGGAAGTATTAGAGAA----A--------AAAA---
-------GCA-AAGTAACGCTTTATAGTGATTCTAAATATGTGGTTGATGCGATTGAAAA
---AGGGTGGGCTGAACGTTGGCAAACCAATGGCTGGAAACGAAATAAAAAAGAATTAGC
CATGAACCCCGATCTTTGGGAACAGTTATTAAAACT-GTGT-T-C-A-CAGCA-------
---------------TCAAGTAAAATTTGTTTGGGTCAAAGGTCATGCAGGCAATC-GTG
AAAATGA--ATGTTGCGATCGCCTAG---CAGT-TCAAGC---CTG-TCA----------
-----------------A---CAG----CA---A-A--ATCTA---------------C-
--------T--TC--A---------------------------------------------
----------AG----ATTTAG------------GC-------TAT-G----AAAA----
-TCC--AGAGATGC-AGCA--AATTT---C-GTT--GTTTTAA
>Lyngbya_sp_CCY_8106
AA-AAT-TCATCAAA-ACTTCAAGAAG--TAATATT-GTATACTGATGGGGCTTGTCA-G
GGAAATCCGGGGCCAGGTGGTTATGGGATTGTGTTAAT--TCGTGGA-GAT--CA-----
------TC--G-AGAAGAATTATCGGGGGGTTTTCAGTTCACAACCAATAATCGCATGGA
AATGA-TGGCGGCGATTGTTGGACTGGAAGTTTTAGATAA----A--------AAAA---
-------GTA-AAGTTAAGCTTTATAGTGATTCTAAATATGTGGTTGATGCAATTGAAAA
---AGGGTGGGCAGAACGTTGGCAAGCTAATGGCTGGAAACGAAATAAAAAAGAATTAGC
CATGAATCCGGATCTTTGGGAACAGTTACTAAAACT-TTGT-T-C-A-CAGCA-------
---------------TCAAGTAAAATTTGTTTGGGTCAAAGGTCATGCAGGAAATC-GCG
AAAATGA--ATGTTGTGATCGCCTAG---CAGT-TCAAGG---CTG-TCA----------
-----------------A---CAG----CA---A-A--ATCTA---------------C-
--------T--GC--A---------------------------------------------
----------AG----ATGTAG------------GC-------TAT-G----AAAA----
-TCC--AGAGATGC-AGCA--AATTT---C-GTT--GTTTTAA
>Roseobacter_denitrificans
```

```
AT-GCC-TGA-GT-T---------------GTTCGC-CTATACGGATGGTGCCTGTTC-G
GGCAACCCGGGCCCCGGCGGCTGGGGCGTTTTGCTGCAGGCCAAAGAGGG---CGACAGG
C-TGGTCAAGGAACGCGCGCTCAAGGGCGGTGAGGCACATACCACCAACAACCGGATGGA
GCTTT-TGGCCGCCATCAACGCGCTGGAATCCCTCAGCCG----C--------GCCA---
-------GCA-CGATCACCGTCGTGACCGACAGCAACTACGTCAAAAATGGCATCACCGG
---C---TGGATTCATGGCTGGAAGCGAAACGGCTGGAAAAATGCCGCCAAGAAACCCGT
CGCGAACGCAGAACTCTGGCAAAGGCTGGATGAGGC-CAA--C-GCA-CGTCA-------
---------------CGACGTCACGTGGAAATGGGTCAAGGGGCACGCCGGGCATG-CGG
AAAACGA--ACGCGCGGACGAATTGG---CGCG-CG---C---G---G--GC--------
----------------AT--GGCC----CC------------G---------------T-
--------T---------------------------------------------------
------------------------------------------------------------
------------------C------------A-A--ACCATGA
>Roseobacter_sp_MED193
AT-GGC-TGA-TC-T---------------CTATGC-CTATACCGATGGCGCCTGCTC-T
GGCAATCCCGGCCCTGGCGGCTGGGGCGCCCTGTTGCAGGCCAAAGATGG---TGGTA-G
CGTCATCAAGGAAAAAGAACTCAAAGGCGGCGAGGCCAATACCACCAACAACCGGATGGA
GCTAC-TAGCTGCCATCAATGCACTGGAAAGTCTAGACCG----C--------CCCT---
-------CGG-CGCTGACCGTGGTGACCGACAGCAATTACGTGAAAAACGGCATCACCGG
---C---TGGATCTTTGGCTGGAAGAAAAACGGCTGGAAAAACGCCGCCAAAAAACCGGT
TAAAAATGCCGAGCTGTGGCAGCGTCTGGATGCGGCGCAA--A-G-C-CGCCA-------
---------------TCAAGTGACCTGGGAATGGGTCAAAGGTCACGCAGGCCATC-CCG
AAAACGA--ACGCGCCGATGAGCTGG---CCCG-CG---C---A---G--GT--------
----------------AT--GGCC----CC------------G---------------T-
--------TC--------------------------------------------------
-----------A----AGAAAA------------GC-------A----------------
-------------A-ATCC------------A-A--AGCATGA
>Escherichia_coli_K12-_W3110
AT-GCT-TAA-AC-A----------GG--TAGAAAT-TTTCACCGATGGTTCGTGTCT-G
GGCAATCCAGGACCTGGGGGTTACGGCGCTATTTTACG--CTATCGCGGA---CG-----
------CG---AGAAAACCTTTAGCGCTGGCTACACCCGCACCACCAACAACCGTATGGA
GTTGA-TGGCCGCTATTGTCGCGCTGGAGGCGTTAAAAGA----A--------CATT---
-------GCG-AAGTCATTTTGAGTACCGACAGCCAGTATGTCCGCCAGGGTATCACCCA
---G---TGGATCCATAACTGGAAAAAACGTGGCTGGAAAACCGCAGACAAAAAACCAGT
AAAAAATGTCGATCTCTGGCAACGTCTTGATGCTGCATTG--G-G-G-CAGCA-------
---------------TCAAATCAAATGGGAATGGGTTAAAGGCCATGCCGGACACC-CGG
AAAACGA--ACGCTGTGATGAACTGG---CTCG-TG---C---CGC-G--GCG-------
-----------AT---GA--AT-C----CC------------A---------------C-
--------ACTGG--A--------------------------------------------
----------AG----ATACAG------------GC-------T----------------
----------------ACC--AAG-T---T-G-A--AGTTTAA
>Alteromonas_macleodii_English_Channel_615
GT-GGC-GCA-AA-A-GA---C---CA--TTCATAT-TTATACCGACGGCTCCTGTCT-A
GGCAACCCAGGCCCTGGTGGGTATGGTGCTGTATTAAT--TTATAAACAG---CA-----
------TA---AGAAAGAGCTAAGCGACGGATTTGCGCATACCACAAATAACCGCATGGA
GCTGC-TGGCCCCTATCGAAGCGCTAAACAGCCTGACTGA----G--------CCTT---
-------GTG-CGGTAGAACTCACTACCGACAGCCAGTATGTAAAAAATGGGATTAACCA
---A---TGGATCCATAACTGGCGCAAAAACGGTTGGCGTACGACTGATAAAAAGCCAGT
GAAAAATGCCGATTTATGGCAACGGTTAGATGAAGCAGTG--A-A-A-AAGCA-------
---------------TCAAGTGAATTGGCACTGGGTCAAAGGCCACTCGGGGCACC-CCG
AAAATGA--ACGCTGCGATGAGCTTG---CTCG-CGGGGC---CGC-C--GAA-------
-----------GC---GA--AA-C----CA------------A---------------C-
--------TCAAA--T--------------------------------------------
----------CG----ATGAAG------------GT-------T----------------
----------------T-----TG-T---T-G-G--CAACTAA
```

>Candidatus_Pelagibacter_ubique_HTCC1062
AT-GAT-T-----------------------AAAAT-TTATACAGATGGATCATGTTT-A
AATAACCCCGGTAATGGTGGTTGGGCTGCAATTATAAA--CCAAAAT-GG---TGA----
-------A--GTCAAAGAAATTAGTGGGAGCGTAAAAGACACTACTAATAATAAAATGGA
ATTGA-TGGCTCCTATTATGGCTCTAAAACAAATTAAACA----A--------AATG---
-------ATC-AAATTGAAATCTATACCGACTCACAATATGTAAGATTAGGAATTACTGA
---A---TGGGTGCATAAATGGATAAAAAATAATTGGCAAACTTCAAAGAAAGAACCTGT
TAAGAATAAAGAACTTTGGATACAACTATACGAGCT-AACA-A-A-T-CAATT-------
---------------TGAAATAAAATGGATTTGGGTTAAAGCACATGCAGGTAATG-CCC
TAAATGA--AGAAGTAGATTTACTCG---CAAA-AAA--A---G------CTG-------

----------------CTG----------------A------------------------
-----------------AT--------------T--AAACTAA
>Alpha_proteobacterium_IMCC1322
AT-GAG-CAC-TA-G----------TG--TGACGAT-CCATACCGACGGGTCATGCCT-G
AGCAATCCTGGCCCCGGCGGCTGGGCAGCGATTCTGAA--TTGGCGG-GG---CAC----
-------G--GAAAAAGAATTATCAGGTAGTGTGGCCGATACGACCAACAATCGCATGGA
ATTGC-AAGCCGCCATTGAAGGACTGAATGCGCTAAGCCG----T--------CCAA---
-------TGC-AGGTCGAACTTCATACTGACAGCAAATATGTCATGAATGGCGTCACTGA
---C---TGGATGGCACGCTGGAAAGCAAATGGCTGGCGTACGGCGGCCAAGAAACCTGT
CGCCAATCAGGATTTATGGGAACAGCTTGATGCGGC-CACC-C-A-A-CGTCA-------
---------------TCAGATCACCTGGCATTGGGTTAAAGGTCATGCAGGTAACG-AAC
TCAATGA--ACGTTGTGACGTGCTGG---CACG-AAG--C---G------CCG-------

----------------CCG----------------A-------A----------------
---------------AGCC--------------T--GAAGTGA
>Marinobacter_sp_ELB17
AT-GAG-CGG-TA-A----------CG--TGATTAT-GTACACTGACGGCGCCTGCAA-G
GGCAACCCCGGGCGCGGCGGTTGGGGAGTGGTGCTGCG--CTGGGGC-GA---GGT----
------TT--G-CAAAACCTTGCACGGTGGCGAGCAGCACACCACCAATAACCGCATGGA
GCTGA-TGGCCGCGATTGAAGGGCTGAAAGCGTTGAAGCG----T--------GATT---
-------GTG-ACGTAGAGCTATATACCGACTCCCAGTACGTGCGCAAAGGCATCACCGA
---G---TGGCTGGCCGGCTGGAAACGCAACGGTTGGAAAACAGCGGCTAAAAAGCCGGT
TAAAAATGACGATCTTTGGAAAGCCCTGGATGAACA-AAGC-G-A-G-CGCCA-------
---------------CCGGGTAAACTGGCACTGGGTGAAAGGGCACGCCGGCGTGC-CGG
ATAACGA--ACTGGCCGACCAGCTGG---CCAA-CCA--A---G------GCG-------

----------------TTGAAG-----------AGC-------T----------------
---------------GACC-------------------GGTTAA
>Marine_bacterium_HP15
AT-GAC-CGG-CA-A----------GG--TAACCCT-CTATACGGATGGCGCCTGCAA-A
GGCAACCCCGGGCCCGGCGGCTGGGGCGTGGTTTTGCG--CTATGGC-GA---CAA----
------TC--G-GAAGACCCTGCACGGCGGGGAGACGAATACCACGAACAACCGCATGGA
ATTAA-TGGCAGCCATCCGTGGTCTGGAGGCCCTGAAGCG----C--------CCGT---
-------GCG-AGGTGGAGCTGTTTACGGATTCGCAGTACGTGCGCAAAGGCATTACGGA
---A---TGGATGGCAGGCTGGAAGCGGAACGGCTGGAAGACCTCCGCGAAAAAGCCCGT
GAAGAACGAAGATCTTTGGCGCGAGCTGGATGC-CG-AAGTGG-C-C-CGGCA-------
---------------CCAGATTAACTGGCACTGGGTAAAAGGGCACTCTGGTGTTC-CTG
ACAATGA--GCTGGCGGATGAACTGG---CCAA-TCG--C---G------GCG-------

----------------TCGACG-----------AGC-------T----------------

----------------TTCC------------G-G--CGCATAG
>SAR86_cluster_bacterium_SAR86A
AT-GAA-AAC---------------AG--TAAATAT-ATATACAGACGGTGCATGCAG-A
GGAAATCCGGGAGATGGTGGATGGGGTGCTTTAGTAGA--GTATCAA-AA---TTT----
------TA--G-TAAAGAGTATTTTGGTGGCCAGAAAAATACTACTAATAATCAAATGGA
ATTAA-AAGCTGCAATTGAGGGTCTTAAAGCATTGAAAGA----A--------ACAT---
-------GCA-ATGTTAATTTAACCACTGATTCGAAATATGTTATGGATGGAATTACTTC
---T---TGGATAGATAATTGGAAAAAAAATAATTGGAAAAGTTCATCAAAAAAGGATGT
TAAAAATAAAGATCTATGGATAGAGTTGGATAAATA-CAGG-T-C-T-ATGCA-------
---------------TAATGTAAAATGGATTTGGGTTAAAGGCCACTCAGGGCATG-AGA
AAAATGA--AATCGCTGATATGTTGG---CAAA-CAA--G---G------GTA-------

----------------TAG----------------A-------T----------------
-------------------------------A-G--CATTTAA
>Synechocystis_sp_PCC_6803
AT-GGC-CTC-TA-C-CCCCAATTCCG--TAACTCT-CTACACCGATGGTGCCTGCTC-C
ATGAATCCTGGCCCTGGTGGCTATGGCGCAGTGATTCT--CTACGGT-GATGGTC-----
------GT--CGGGAGGAATTGTCCGCCGGTTATAAAATGACCACCAATAATCGCATGGA
AATTA-TGGGGGCGATCGCCGCCTTGAGTCATTTACAA-G----A--A-----CCGT-C-
C--------C-AAGTGTTGCTCTATACCGATTCCCGCTATATGGTCGATGCCATGAGCAA
GGGT---TGGGCCAAGAAATGGAAAGCCAACGGATGGCAAAGGAATGCCAAGGAAAAGGC
CAAAAACCCCGACCTGTGGGAAACGATGTTAACGCT-GTGC-G-A-A-AAACA-------
---------------TCAAGTTACATTCCAGTGGGTCAAAGCCCACGCTGGCAATA-AGG
AAAATGA--GCGGTGCGATCGCCTAG---CGG-------T---GGC-CGCCTA-------
-----------TC---AA---AAT----A-------------------------------
------------------------AT----------------------C--CCA----A-
----CTTAGTAG----ATGAAG------------GC-------TT---------------
-----------------C------GG---C-A-A--ATTCTAG
>Synechocystis_sp_PCC_6714
AT-GGC-TCC-CA-C-TGCAAACTCTG--TAACCCT-CTACACCGATGGTGCCTGCTC-G
AGAAACCCTGGCCCTGGAGGCTATGGGGCAGTGATTCT--TTACGGT-GATGGTC-----
------GC--CGAGAGGAGTTGTCTGCCGGTTATAAAATGACCACCAATAACCGCATGGA
AATGA-TGGGGGCGATCGCCGCTTTAAGTTATTTGGAA-G----A--A-----CCCT-C-
C--------C-AGGTGTTGCTCTACAGCGATTCCCGCTATGTGGTGGATGCAATGACTAA
GGGC---TGGGCCAAGAAATGGCAAGCGCAAGGATGGCAAAGAAACGCCAAGGAAAAGGC
TAAAAATCCGGATCTCTGGGAAATAATGTTAGCCCT-ATGC-A-A-A-AAACA-------
---------------CCAGGTCACCTTTCAGTGGGTCAAAGCCCATGCTGGCAATA-AAG
AAAATGA--ACGGTGCGATCGCCTAG---CGG-------T---GGC-GGCCTA-------
-----------CC---AA---AAC----A-------------------------------
------------------------AT----------------------C--CCA----G-
----CTTAGTGG----ATAAGG------------GC-------TT---------------
-----------------T------GG---C-A-A--GTTCTGA
>Chloroflexus_sp_Y-400-fl
TT-CTTCCTC-CA-C-AAACGGGATTG--TGATGAT-GTACACCGACGGTAG-TGCTCTC
GGCAATCCTGGGCCGGGCGGGTACGGCGTTGTGCTGCG--CTACAAT-GACCA-------
-------T--TACCAGGAACTCACCGGTGGTTTTCGGCTCACGACCAACAACCGCATGGA
GTTAA-TGGCCTGTATTGTCGGGCTGCGTGCACTCAAG-C----G--A-----CCGG-T-
G--------T-CGGTACGCCTGCACAGCGACTCGCGCTACGTCGTCGATGCTTTTCGCAA
AGGC---TGGATACACCGCTGGCAGGCACAAAACTGGATGCGCAGCGAGACCGAACCGGT
AAAAAATGCCGATCTCTGGGCTGAATTGCGCTTGCT-GTGT-G-A-A-GGTCA-------
---------------TCAGGTTGAGTTCGTCTGGGTGCCCGGTCATCAGGGCGTAC-CTG
ACAATGA--ACGTTGTCACGAACTGG---CCA-------T---CGC-TGCCGC-------
-----------AG---CT---CGC----C-------------------------------
------------------------CA----------------------G--ACC----TG

CCGCC-----GG----ATGTTG------------CCTTTGAGATG---------------
-----------------CAGTGGAGT---C---G--CACATAA
>Synechococcus_sp_JA-3-3Ab
CACGGC-TGGCCG-ATTGCCCTCCTAG---TGGCAT-TTTTACCGACGGGGGCTGTTC-C
CCCAATCCAGGGCCAGGGGGATGGGCGGTGGTGGTGGT--TGAGGAT-GACCAGA-----
------TT--GTGGATGAGTTTTACGGAGGGGATCCCGCCAGCACCAACAACCGTATGGA
ATTGACCGGCCTC-ATTCG-GGCTTTGCAATATTGTCGCGA----C-A-----CCCAGAG
CAA-CAGC-A-TAGACACCATCTACAGCGATTCCCACCTTTGCGTACAGACCTACAACGA
---T---TGGATGGAGCGCTGGGCAGCGCAGGGGTGGCGGCGG--CGC-ACGGGGCCGGT
GGAAAACTTGGATCTGGTGCAGGAGCTGTATCAACTCAAGC-AGC-AGTGCCC-------
---------------GCAGGTGCGGGTGGCCTGGATCCCGGCCCATCGGGGTCTGCGCTG
G-AACGAGTATGTG--GATGAGCTGGTCGC------------------------------

---CC---------------
-----------------GC-GCCCGA---C---C--CAAGTAG
>Synechococcus_sp_JA-2-3Ba
GCCGTC-TGGCTG-CCTGCCCCCGCAG---CGGCAT-CTTCACGGACGGGGGATGTTC-T
CCCAATCCGGGGCCAGGGGGATGGGCCGTGGTGGTGGT--TCAAGAT-GATCGGG-----
------TT--GTGGAAGAGTTTTGGGGAGGGGATCCCGATAGCACCAGCAACCGCATGGA
GTTGACCGGCCTC-ATCCG-GGCTTTGCGGTATTGCCGCCA----C-------CCCAGGG
CAA-CGGC-G-TCGATACCATCTACAGCGACTCCCACCTTTGCGTGCAGACTTACAACAA
---T---TGGATGGAGCGCTGGGCAGCCCAGGGATGGCGGCGG--CGC-ACAGGGCCAGT
GGAAAACTTGGATCTGGTTGAGGAGTTATATCAACTCAAGC-AGG-AGTGCCC-------
---------------TCAGGTGCGGGTGGTCTGGATCCCAGCCCACCAAGGTCTGCGCTG
G-AACGAGTACGTG--GATGGGTTGGTTGCCC-------A---GGC--------------

------TCGAGC----CAGAGT------------GACCGTTCCTC---------------
-----------------CAG-AAGTT---A---G--GGGTTGA
>Cyanothece_sp_PCC_7425
AT-GAA-C-G--------CAGAAATTA--GCAAAAT-TTATACTGATGGAGCTTGTTC-C
GGCAATCCTGGTCCGGGGGGATGGGGGGTTGTGGTTTA--CTTTGGG-GATGGCT-----
------CT--GTGCATGAGATGGGGGGGCAGCAGCGGCCACCACAAACAATCGGATGGA
AATGC-AGGCTGCGATCGCCGCGCTGGAATTTTGTCAGGC----T--G-----CCGG-C-
CATC---CCC-CAGTTATCCTCTATACCGACAGTGAGTATGTCAAAAAAGGGATTACAGA
---G---TGGCTACCAGGCTGGAAACGCAAGGGATGGAAAACCGCTCAGGGTAAACCCGT
GCTCAATCAAGACCTCTGGCAGGTGCTGGATCAACT-G----A-A-C-ACCAA-------
---------------AAGTGTGAACTGGCAGTACGTGCGGGGCCACTCTGGTAATG-CTG
GCAATGA--GCGCTGTGATGCGATCG---CCCG-AGCCTT---TGC-CGCCGG-------
-----------AA---GG---CAA----CC---A-G------------------------

---------------------A----G-----CTGC------------------------
---------------G-TC--AGAGC---C-G-AGACCTGTAA
>Halothece_sp_PCC_7418
AT-GTC-TCA-AC-T-CCCCGAAATTG--ATTGTAT-TTATACTGACGGCGCTTGTAC-G
GGAAACCCAGGCCCTGGCGGGTGGGGCGCTGTCTTTTA--CTTTAAA-AATGGTT-----
------CA--GTATATGAAATCGGTGGCGCGGAGCCGGAAACGACCAATAATCGGATGGA
AATGC-AAGCAGCGATCGCGTCATTAGAGTTATTTCAAGC----C--A-----GTGA-A-
CAAAAAACAC-CGATTGTTCTCTACAGTGACAGTCAGTATCTGATTAAAGGTCTCACCCA
---A---TGGCTCAAAGGCTGGAAGAAAAAAGGCTGGAAAACGTCAACGGGCAAAGCTGT
TTTAAATCAAGACCTGTGGACAACATTAGACCAACT-G----A-ATC-ACTCT-------
----------------CTTATTGAGTGGAAGCACGTGCGCGGTCACGAAGGAATTG-CAG
GGAATGA--ACGGTGCGACGCGATCG---CGCG-GACTTT---TGC-TGCTGG-------
-----------AA---AA---CGT----CC---T-C--ATTT------------------

```
-----------AA--A-GAAAAATTT-----------------------AGATT----T-
-------TCCTC----TTCCCG-C---T-----AAC------------------------
---------------T-CC--GATAC---C-G-TAAT-GAGTG
>Microcoleus_sp_PCC_7113
AT-GCC-TTC-TA-C-TCGCCAAATTA--AATGTTT-TTACACCGATGGCGCTTGCGC-T
GGCAACCCTGGTCCCGGCGGCTGGGGTACTGTAGTTTA--TTTTACC-GATGGCT-----
------CC--GTTTATGAAATGGGTGGTGGGACTGCTCAAACCACAAACAACCGAATGGA
AATGC-AAGCAGCCATTCAGGCGTTGAAGGTTCTCAAGGC----A--T-----CGGC-T-
CAAACCGAAT-CCGTCATCCTCTACACCGATAGTGAATATGTCAAGAATGGCGTCACCAA
---A---TGGGTGAAAGGCTGGAAAAAGAAAGGCTGGAAGACAGCACAGGGAAAAGCTGT
TTTAAATCAAGATTTGTGGGAAACTCTTGATGAGCT-G----A-A-T-TCTCC-------
---------------CTTGGTGGAGTGGCAGTATGTTCGGGGGCACAGTGGCAATG-AGG
GCAATGA--GCGTTGTGATACAATCG---CTCG-TGCTTT---TGC-TGTTGG-------
-----------CA---AG---C------CC---T-TGGTACTC---------------G-
--------A--TC--A-GGCGTTCGA-----------------------CCTGA----G-
-----TAC--TC----CACCCTCC--A------ATC------------------------
-------------A-CTCA--AAC----------TCCAACTCA
>Synechocystis_sp_PCC_7509
AT-GTC-TTC-TT-G-CGGCACAATTC--AAAGCGT-GTATACAGATGGAGCTTGCAC-T
GGCAATCCTGGCCCTGGGGGTTGGGGCGTAGTTGTTTA--CTACACA-GATAAAT-----
------CA--GTGCGCGAATTTGGCGGCAATGAAAAGCAAACGACCAATAATCGTATGGA
GATGC-AGGCAGCGATCGCAGCATTAACTTTTTTAACCGA----A--A-----CACC-G-
CCCACAGAAC-CAATAACGTTGTATACCGATAGCGAATATTTAATTAAAGGTGTAACTCA
---G---TGGGTAAAAAATTGGAAAAAAAAAGGCTGGAAAACCGCCACTGGTAAGCCCGT
ACTAAATCAAGACCTGTGGGAAAACCTCGACCAATT-G----A-A-T-AGTAA-------
---------------ATTGGTATTGTGGCAACACGTCCGGGGTCATGCGGGAAATG-TGG
GTAATGA--AAGGTGCGATGCGATCG---CACG-GGCGTT---TGC-TAATGG-------
-----------AA---AA---A------CC---T-TATCGCTA---------------C-
--------A--AC--A---AGGCAAA-----------------------CC---------
--------AAAC----CTCCCA-C---A-----AAC------------------------
---------------AACA--GAAT--------TAGCTGAAAA
>Cylindrospermopsis_raciborskii_CS-505
AT-GTC-AAA-TC-A-ACGCATAATTA--AAAGTAT-ATATACCGATGGAGCTTGCAA-G
GGAAACCCAGGTCCTGGTGGTTGGGGTGTGGTTGTTTA--CTTTCAT-GATGGTT-----
------CA--ATTCATGAAATGGGGGAAGCATCTGACTACACTACAAACAATCAAATGGA
AATCC-AAGCTGCGATCGGGGCACTTGAATTATTAGCTCA----A--T-----CCGG-A-
CAAGTTGAAC-CTATTACTCTATATAGCGATAGCGAATATCTAATTAATAGTGTGACTAA
---A---TGGATACATGGATGGAAAAAAAAGGGTTGGAAAAAATCAGATGGCACTCCGGT
GCAAAACCAAGAGCTACTAAAAATTATAGACAAGCT-G----A-A-C-ACCAA-------
---------------GCTAGTCAGGTGGCAACATGTACGTGGACATGCGGGTAATA-TAG
GTAATGA--AAGATGCGATGTTATTG---CCCG-TTCTTT---TGC-GAGTGG-------
-----------CG---AA---ATT----CC---C-C--CTTTG---------------C-
--------A--AC--A---A--TTAT----------------------C--CCC----C-
----C---AA------CAGTTG-C---TAAC--TAA-------TT---------------
-------------G-TCAA--GAAAC---C-T-T--AACCCAA
>Raphidiopsis_brookii_D9
AT-GTC-AAA-TC-A-ACGCATAATTA--AAAGTAT-ATATACTGATGGAGCTTGCAA-G
GGAAACCCAGGTCCTGGTGGTTGGGGTGTGGTTGCTTA--CTTTCAT-GATGGTT-----
------CA--ATTCATGAAATGGGGGAAGCATCTGATCACACTACAAACAATCAAATGGA
AATCC-AAGCTGCGATCGCAGCCCTTCAATTATTCGCTCA----A--T-----CCGG-A-
CAAGTTGAAC-CTATTACTCTGTATAGTGATAGCGAATATCTAATTAACAGTGTGACTAA
---A---TGGATACATGGATGGAAAAAAAGGGGTTGGAAAAAATCAGATGGCACTTCGGT
GCAAAACCAAGATCTACTAAAAGTTATAGACAAGCT-A----A-A-C-ACCAA-------
---------------TCTGGTCAAGTGGCAGCATGTACGTGGACATACGGGTAATA-TAG
GTAATGA--AAGATGCGATGTTATTG---CCCG-TTCTTT---TGC-GAGTGG-------
```

-----------CA---AA---ATT----CC---C-C--CTTTG---------------C-
--------A--AC--A---G--TTAT----------------------C--CCC----C-
----C---AA------CAGTTG-C---TAAC--TAA-------TT---------------
--------------GTCAA--GAAAC---C-T-T--AACCCAA
>Nostoc_sp_PCC_7120
AT-GTC-TAC-CC-A-ACCGATAATCG--AAAGTAT-ATACACTGATGGTGCTTGCAC-T
GGCAATCCTGGCCCTGGCGGCTGGGGTGTAGTTGTCTA--TTTTAGT-GATGGTT-----
------CA--GTTCACGAAATGGGCGATGCTGCCAAGCATACCACCAATAATAAGATGGA
AATGC-AGGCGGCGATCGCCGCTCTCAAATTTCTCCACGA----C--T-----CCGG-A-
CAAGCTGAAC-CCATTACCCTCTATACCGATAGTGAGTATCTAATTAACTGCGTCACCAA
---G---TGGGTAAAAGGTTGGAAAAAAAAGGGTTGGAAAAAATCTGATGGGAATCCAGT
TCAGAATCAAGACCTTCTAGAAACTTTAGATGAATT-A----A-A-T-AGCCG-------
---------------TAAGGTAAATTGGCATCATGTTCGAGGTCATTCTGGCAACA-TCG
GTAACGA--GCGTTGTGATGTGATTG---CTCG-CTGTTT---TGC-AACTGG-------
-----------TA---GA---ATG----CC---C-T--CTCTA---------------C-
--------A--AC--A---G--TTAT----------------------C--TCC----C-
----C---GT------CA--TG-CTCA---TAAATC-------TT---------------
-------------T-ACCT--GACAT---C-A-G--TAAAATA
>Rivularia_sp_PCC_7116
AT-GTC-TTC-TC-A-GCCTACAATTC--AAAATAT-ATATACTGATGGCGCTTGTAC-C
GGCAATCCAGGTCCCGGTGGTTGGGGTGTTGTTGTTTA--CTTCGAT-GATGGTT-----
------CG--ATTCACGAAATCGGTGATGCATCAAAAAAAACCACCAATAACAGGATGGA
AATGC-AAGCCGCGATCGCAGCTTTGGAATTTTTAGAATC----T--T-----CCGG-G-
CAAAACAAAC-CCGTTACTCTTTACACCGACAGCGAATATCTAATTAACTGCGTTACGAA
---G---TGGGTGAAAGGATGGAAGCGTAAAGGTTGGAAGAAATCCGACGGAAAACCCGT
GCTTAACCAAGATTTATTGGAAATTCTAGATAATCT-T----A-A-T-AATCA-------
---------------AAAGATTAAATGGCAACACGTTCGCGGTCACGCTGGTAATA-TAG
GTAACGA--ACGTTGCGACACAATCG---CCCG-TGGTTT---TGC-TTCAGG-------
-----------GA---AA---ATA----CC---A-T--CGCTA---------------A-
--------A--GC--A---A--ATGC-----------------------T-G------G-
--ATA-----------TAAATT----TCAGTGATGC-------TA---------------
-------------A-TT-----TCGCACAA-A-A--AGCCGAA
>Nodularia_spumigena_CCY9414
AT-GTC-CAA-CC-A-ACGCAAAATTC--AAAGCAT-CTACACCGATGGCGCTTGTAC-T
GGGAACCCTGGCCCTGGGGGTTGGGGTGTGGTCGCTTA--TTTCAGT-GATGGCA-----
------AA--ATCCACGAAATGGGTGATGCTGCCAGCTATACGACTAATAATAAAATGGA
AATGC-AAGCGGCGATCGCAGCTTTAAATTTCTTCCACGC----A--T-----CAGG-A-
CAAGCTGAAC-CCATCACCCTCTATACCGATAGCGAATACTTGATCAACTGCGTGACTAA
---G---TGGTTACCAGGATGGAAAAAAAGAGGCTGGAAAAAAGCAGACGGTAAACCTGT
CCAGAACCAAGAACTTTTAGAACTTCTCGATCAACT-C----A-A-T-ACTCG-------
---------------CCAAGTAAATTGGCAACACGTCCGGGGACATTCTGGTAACA-TAG
GTAACGA--GCGCTGTGATGTGATTG---CTCG-CTCCTT---TGC-TAGCGG-------
-----------TC---AA---ATC----CC---A-T--CTCTA---------------C-
--------A--AG--A---A--TTAT----------------------C--C------G-
----C---TA------CTGAGT-C---CTACAAATC-------TT---------------
-------------T-AC-----ATTCTTTA-G-G--TGAAGCA
>Calothrix_sp_PCC_7103
AT-GGC-TTC-AC-T----AACAATTG--AGAGTAT-ATACACAGACGGCGCCTGTAC-A
GGGAACCCAGGACCAGGAGGTTGGGGTGTGGTGGTTTA--TTTTGAT-GATGGTT-----
------CG--GTTCATGAACTTGGAGATGGTTGTCGTCAAACAACGAATAACAGGATGGA
AATGC-AAGCTGCCATCGCAGCACTTCAATATTTGGAAGC----G--T-----CAGG-G-
CAAAGTTACC-CTATTACTTTGTACACAGATAGTGAGTATTTGATCAATTGCGTTACCAA
---G---TGGGTTAAAAGCTGGAAAAAGAAGGGTTGGAAGAAAGCTGATGGTAAGCCAGT
TCAGAATCAGGATTTACTCGAAATTCTTGACGGACT-T----A-A-C-AGTCG-------
---------------TAAGGTTAGATGGGAACATGTTCGAGGTCATGCTGGTAATA-TAG

GTAACGA--AAGATGCGATACCATTG---CGCG-GGCTTA---TGC-GGCTGG-------
-----------AA---AA---GTG----CC---A-G--TATTA---------------A-
--------A--GC--AAAGC--CAAG-----------------------ACG------C-
--AGA-----AA----CTATTG-C---TTTC--G-A-------TG---------------
-------------T-AAAA--ACAAA---A-G-T--TTATGTA
>Richelia_intracellularis_HH01
AT-GTC-TCC-AG-T-TCATAAAATCC--AAAGTAT-ATACACCGATGGTGCCTGCAC-T
GGGAACCCTGGTCCTGGTGGTTGGGGTGTGGTTATCTA--TTTTAAT-GATGGTT-----
------CG--ATTCATGAAATAGGCGAGCCATGTGATTGCACTACTAATAACCGGATGGA
GATAG-AAGCTGCAATTGCAGCTTTGCGATATCTGCAAAC----T--A-----GTGA-A-
AAAAATGAAC-AAATTACCCTTTACACAGATAGTGAATACTTAATTAACTGTGTAACAAA
---A---TGGGTAACAGGCTGGAAAGCAAAGGGGTGGAAGAAAGCCGATGGCAAACCAGT
TGTTAATCAAGACTTGCTAGTAACTTTAGATGAACT-T----A-A-C-AGTCA-------
---------------TCACATAAAATGGCAACATGTCAGGGGTCATTCAGGAAATG-TGG
GAAATGA--ACGTTGTGATATGATCG---CTCG-TGCATT---TGC-CAATGG-------
-----------TA---CC---GAA----CC---A-T--TCCTA---------------A-
--------C--AC--A---A--ATTT----------------------CT-A------G-
----C---TGCA----ATTCAC-A----ACC-AC-A-------AA---------------
--------------ATGAA--TATGA---T-T-T--GGCTGCA
>Leptolyngbya_sp_PCC_7104
AT-GGC-AAT-TA-T----------TG--AGCGAAT-CTATACAGACGGAGCCTGCTC-C
GGCAATCCTGGCCCCGGCGGCTGGGGAACCGTGCTGTA--CCTGGCC-GAGGGCG-----
------GC--GTGCATGAGCTGGGCGGCGGCGAGGCCCAAACCACCAACAACCGGATGGA
AATGC-AGGCTGCGATCGCAGGTCTGACCCTGCTGCGCGATAGCG--G-----CCA----
--GACCCAGC-CTGTCACCCTGCATACCGATAGCGAGTACGTGCTCAAGGGCATCACCCA
---G---TGGATTGCCGGCTGGAAGCGGCGGGGCTGGGTGAACTCGGCCAAAAAGCCGGT
CCTCAACCGCGACCTGTGGGAGGTTTTGGATGCCTT-G----A---C-CACCGAGGTCAA
TCAAACCCTCGATCGCCCCCTCCAGTGGGTCTACGTGCGCGGCCATTCGGGCGA-T-GTG
GGC------AACGAGCGCTGCGA-CA---C-------CAT---TGC-CCGGGC-------
-----------CT---TT---GCG----GC------------C-----------------
-------------------------------------AACCGCGCGATC--GCC----C-
----------------TGGCTA-------------C------------------------
-----------------GG--CATCG---T---T--GCCCTAG
>Leptolyngbya_sp_PCC_7376
AT-GAT-CA--------------------AACGAAT-TTATACCGACGGCGGCTGCAT-C
AATAATCCCGGTTCCGGTGGCTGGGGCGTTGTGATTCA--ATATCAA-AATGGCT-----
------CT--GTTTCCGAACTTGGCGATCATGAAGCCCAAACCACAAATAATCGCATGGA
GATGC-AAGCTGCCATCGAAGCCCTCAAACAATTTCGAGATAGCG--A-----TCA----
--GCGCGAAC-CCATCCAGCTCCTTACCGATAGTAAATACGTAATTGATGGCATTACGAA
---G---TGGATTAAAGGCTGGAAACGGAAAGGCTGGAAAAAATCATCCGGTGCACCAGT
CCTCAATAAAGATCTCTGGCAGGAAATGGATAAACT-G----A-A-T-CACCC-------
---------------TGCTGTGCAATGGACTCACGTTAAAGGCCATAGCGGTGAGC-CTG
GGAATGA--ACGTGCCGATGCGATCG---CCAA-CAGTTT---TGC-CCGTGG-------
-----------CA---AA---GCA----CC------------C---------------A-
-TTTT-AAA--AA--C------ACCTCGTTTACAAAACGGCGAAAAA-AT-ACA----A-
----------------AATCTT-------------C------------------------
-----------------CC--AAATC---C---A--GAGATAT
>Gloeobacter_kilaueensis_JS1
AT-GAT-C-----------------------CGGAT-CGTTACCGATGGCGCGTGCCT-G
GGCAATCCCGGCCCCGGCGGTTGGGCGGCGTTGAT-----CTTGCAG-GACGATC-----
------AA--ATCGAAGAATACAGCGGCTTCGAGGCCCAGACCACCAACAACCGCATGGA
GATGC-AGGCCGTGATCGAGGGGCTGCGCCGCGTGGGCG----CT--A-----CTG----
-------TCC-CGGTGACGGTAATCTCCGATAGCCAGTACGTCATCAAAGGCATGAGCGA
---A---TGGCTGCCCGGCTGGAAACGCCGCCAGTGGCGCACCGCCGCCGGTAAGCCCGT
CGAGAACCGCGACCTCTGGGAAGCACTCGAAAAAGT-G----G-C-G--GCGG-------

--------------GCCGGGTGCAGTGGCAACACGTCTACGGCCATCGCGGCCATG-CTG
AGAACGA--GCGGGCAAACGATCTGG---CCCA-GGCTGCCGCTGC-CCGCAGACCGGTG
CCGCCCGCTGTCG---AA---CCT----GC---TGC--TTTTG---------------C-
--AACCAGC--GC--T------CCCCGCTCAGACGGTATTACCTATC-----TC----A-
----------------GCTTGA-------------T------------------------
-----------------CG--ATGGC---C---A--GATCGAG
>Gloeobacter_violaceus_PCC_7421
AT-GTT-T-----------------------CGGAT-AGCGACCGACGGGGCCTGCAG-C
GGCAATCCCGGTCCGGGGGGATGGGCGGCCCTGGT-----CGTGGGG-GAGGGCA-----
------CC--TACGAAGAAGTGTTCGGTTTCGAGCCGCACACCACCAATAACCGCATGGA
GATGC-GCGCCGTCATCGAAGGTTTGACGCGCGTACCCG----CG--G-----ACG----
-------CCA-AGGTGAAAGTGCTCACCGACAGCCAGTACGTGATCAAAGGGATGAGCGA
---G---TGGCTGCCCGGCTGGAAGCGGCGCGGCTGGATCACTTCCACCGGCAAACCCGT
CGAGAACCGGGATCTGTGGGAAGCGCTGGAGCGGGC-G----G-T-A--GGGG-------
--------------GGCGCGTGCTCTGGGAGCATGTGCGCGGCCACACCGGCCACC-CCG
AGAATGA--GCGGGCCAACACGCTCG---CCCA-GACGGC---AGC-CCGCGG-------
-----------CG---GT---GCT----CC---GGC--CGTTG---------------G-
C-GCC-CGG--AC--T------GTGCCCACCAATGGAACGACCTATCTG--AGT----C-
----------------TGGTGG-------------A------------------------
-----------------CG--GGCAC---C---T--CAG-CCG
>Prochlorococcus_marinus_str_NATL2A
AT-GTC-TAA-AG-A-AGAAAAATTAGCGATTGCTGCAGCAACCGATGGTGCATGCAG-T
GGTAATCCTGGTCCGGGTGGCTGGGGAGCATTAATCAG--ATTCCAG-GATGGAA-----
------GC--GAGATTGAATTCGGTGGGAACTCCCCCGAGACAACGAATAATCGCATGGA
ATTAC-AGGCTGCATTATTTATTCTAGAAAAATTAAAA-AA--CA--T-----AAA--A-
TTTGCC-CCTTCACTAACTATTAAAACTGATAGCAAATATTTAATTGATGGAATGGACAA
---A---TGGATGCCAAATTGGAAAAAGAAAGGCTGGAAAACTGCCTCTGGCAAACCAGT
TCTCAATCAAGATTTGTGGAAAGCTCTTGACCATCC-T----G-A-A-CTACC-------
---------------AAAAATCAAACTTCAATATGTCAAAGGTCATAGCGGAGAAA-AGG
ATAATGA--CAGAGTCGATGCAATTG---CGGT-AGCTTT---TTC-TAAAGG-------
-----------TA---G-GAAAAT----AC---A-A--CT--------------------
---TA-A----A----G-
----------------ATTTTG-------------C------------------------
-----------------------------C-A-A--CAACTAA
>Prochlorococcus_marinus_str_MIT_9313
AT-GGC-TGA-TG-G-TCGTGGACGGGTTGTGGCTGCTGCCACTGATGGGGCTTGTAG-T
GGAAATCCTGGCCCTGGGGGCTGGGGGGCACTGCTGCG--CTTTGAG-GATGGGA-----
------GC--GTGGAGGAGTTCGGAGGCTATGCGCCGGCCACGACCAACAACCGCATGGA
ACTTC-AGGCGGCCCTTCATGTGCTGGAGCAGCTCAAAGAG--CT--T-----CCT--T-
GTCACC-CCG-ATTTAAAGATCCGTACCGACAGCAAATATTTAATTGATGGTTTGAGCAA
---A---TGGATGGCGGGATGGAAACGTAAGGGTTGGCGTACGGCGGCTGGCAAGCCTGT
GCTGAATCAAGATCTCTGGCGAGCTCTAGACCGTGC-T----C-G-C-TTGGA-------
---------------TCATGTGCCCCTGGCTTATGTGAAAGGTCATAGCGGTGACC-CAG
ACAATGA--AAGGGTTGACCAAATCG---CAGTGAGCTTT---T-C-CAAAGG-------
-----------AG---GTGGTACT----CC---A-G--CGTTG---------------A-
-TGTC-TGG--AC--A---G--GGGTCAGC-----------------------C----T-
----------------CTATTG-------------C------------------------
---------------ACTC--GGTCT---T-G-T--TGAGCCG
>Prochlorococcus_sp_MIT9303
AT-GGC-TGA-TG-G-TCGTGGACGGGTTGTTGCTGCTGCCACTGATGGGGCTTGTAG-T
GGAAATCCTGGCCCTGGGGGCTGGGGGGCACTGCTGCG--CTTTGAG-GATGGGA-----
------GC--GTGGAGGAGTTCGGAGGCCATGCGCCGGCCACGACCAACAACCGCATGGA
ACTTC-AGGCGGCCCTTCATGTGCTGGAGCAGCTCAAAGAG--CT--T-----CCT--T-
GTCACC-CCG-ATTTAAAGATCCGTACCGACAGCAAATATTTAATTGATGGTTTAAGCAA
---A---TGGATGGCGGGATGGAAACGTAAGGGTTGGCGTACGGCGGCTGGCAAGCCTGT

```
GCTGAATCAGGATCTCTGGCGAGCTCTAGACCGTGC-G----C-G-C-TTGGA-------
---------------TGATGTGCCCCTGGCTTATGTGAAAGGTCACAGCGGTGACC-CAG
ACAATGA--AAGGGTTGACCAAATCG---CAGTGAGCTTT---T-C-CAAAGG-------
-----------AG---GTGGTGCT----GC---A-G--CGTTG---------------A-
-TGTC-TGG--AC--A---G--GGGTCAGC-----------------------C----T-
----------------CTATTG-------------C------------------------
---------------ACTC--GGTCT---T-G-T--TGAGCCG
>Synechococcus_sp_WH_8102
AT-GGC-TGA-AG-A-ACGTGGTCGGGTTGTGGCTGCAGCCACCGATGGAGCCTGCAG-T
GGCAACCCGGGTCCAGGCGGTTGGGGCGCTTTGCTGCG--ATTTGAA-GATGGCA-----
------GT--GTTGAGGAATTCGGCGGCCATGACCCGGCAACGACCAACAACCGCATGGA
ACTGC-AGGCGGCGCTGGAGTTGCTGCAGCGCCTGAAACAA--CT--G-----CCA--C-
GCCATC-CCG-ATCTGACGATTCGCACCGACAGCAAGTACCTGATCGATGGGCTCGGCTC
---C---TGGATGAAGGGCTGGAAGCGCAAGGGTTGGAAGACAGCGGCTGGCAAGCCTGT
GCTCAACCAGGATCTCTGGAAAGCCCTGGATGCAGC-T----C-G-G-CTGGA-------
---------------TGATGTGCCGCTGGCCTACGTGAAAGGTCACAGCGGTGATC-CGG
ACAACGA--CCGTGTCGACCGGATCG---CAGT-TGCTTT---TTC-CCAGGG-------
----------GCGTTCGT-T--GG----AT---C-T--CGGCC---------------AG
---CCAGCC--GT--T---G--AGAG---------------C----GA-----C----G-
----------------TTGCGG-------------C------------------------
---------------AGTC--GATGT---C-G-A--TGCTGAT
>Synechococcus_sp_CC9605
AT-GGC-GGA-AG-G-ACGGGGACGGGTCGTGGCTGCAGCAACGGATGGTGCCTGCAG-C
GGCAATCCAGGTCCGGGGGGTTGGGGCGCGTTGTTGCG--TTTCGAA-GATGGCA-----
------GC--GTTGAGGAATTCGGTGGCCATGCGCCCGACACTACCAACAACCGCATGGA
ACTGC-AGGCGGCATTGGAGGTGCTGCAACGGCTGGAGCAG--CT--G-----CCC--C-
GTCATC-CGG-ATCTCACCCTGCGCACTGACAGCAAATATCTGATCGATGGCCTGGGCTC
---C---TGGATCAAAGGCTGGAAGCGCAAAGGTTGGAAAACGGCTGCTGGCAAGCCCGT
GCTCAACCAGGACCTCTGGAAGGCCCTGGATGCTGC-C----C-G-T-CTGGA-------
---------------TGACGTCCCCCTGAGCTACGTCAAAGGCCACAGCGGTGATC-CCG
ACAACGA--ACGGGTGGATCGCATCG---CCGT-GGCGTA---CTC-CCATAA-------
-----------CG---CTC---AG----CC---G-C--CCTTG---------------G-
C--TCTGAA--GC--A---G--GGATC------------------G--T----C----G-
----------------GAGCTG-------------C------CCT---------------
-------------C-CTCC--AAGGC---C-G-C--ACCAGGC
>Prochlorococcus_sp_W11
AT-GAA-TAG-TA-G-TCAAAATATTGCAATTGAAGCTGCTACTGATGGAGCCTGCAG-T
GGTAACCCTGGTAACGGAGGATGGGGAGGTTTAATTTT--ATTTGAG-GATGGAA-----
------GT--GAATTAGAAATTGGAGGTTTTGAAAAAAATACTACCAATAATAGGATGGA
ACTTA-TGGCTGCCATCAAAACTCTAGAAAAATTAAAACAA---T--T-----CAA--A-
CTAAAAAAAA-ATTTTAAATTAAGAACAGATAGTAAATACTTAATTGATGGCTATAGTAA
---T---TGGATATATAATTGGAAGAAAAACGGTTGGAAAACAAGTACTGGTAAATTAGT
TCAGAATCTAGATTTATGGCAAAAGATTGATGGATT-A----A-G-A-ATTAA-------
---------------TGAAGTAAGCATGGAATTTGTTAAAGGGCATAGTGGAGATA-AAT
ATAATGA--AAGAGTAGATCTAATAG---CAACTAATTAT---A-G-TAAAGG-------
-----------TA---T------T----TATAA----G---T-----------AGAAAAA
AAGCG-A-----CAAG---A--AAAT---------------ATT--G------ATCAAC-
----------------TTGATT-------------T------------------------
---------------GGCA--G-------C-A-C--CCCAAGA
>Prochlorococcus_sp_W8
AT-GGA-TAT-CA-A-AAATAACATTGCAATCGAAGCTGCTACTGATGGAGCTTGTAG-T
GGTAATCCTGGGAATGGTGGATGGGGTGGCTTAATAAT--TTTTTCT-GATGGCA-----
------GT--GAAATTGAAATTGGCGGTGCTGCAGAAAACACTACCAATAATAGAATGGA
ACTTA-TTGCAGCAATAAAAACAATTGAAAAACTTAAAAAT---T--T-----CAG--A-
TTAAAGGAAA-ATTTTAAATTAAGAACTGATAGTAAATATCTTATAGAAGGTTATACAAA
```

```
---T---TGGATTAAAAACTGGAAAAAAAATGGTTGGAAAACAAGTAATGGAAAATCTGT
ACAAAATTTAGATTTATGGCAACAAATTGACTCTTT-A----A-G-A-ATTCC-------
---------------AGGTTTAACTATGGAATTTGTAAAAGGTCATAGTGGAGATC-CTT
CTAATGA--AAGAGTTGATTTTATTG---CAACAAACTAC---A-G-CAAAGG-------
-----------AA---TAAAT-AT----CG---A----C---C---------------AG
AATTC-A-----AAAA---A--AAAGA-----------------CAT------TTAATT-
----------------TAGATA-------------A------------------------
---------------AAAA--GAAAT---T-G-C--GCCAGAA
>Prochlorococcus_sp_MIT9515
AT-GAA-TAG-TA-A-AACAAATATTGCTATTGAAGCTGCAACAGATGGTGCTTGCAG-T
GGAAACCCGGGACCTGGTGGATGGGGAGGTTTAATAAT--CTTCATT-GACAATA-----
------GC--GAAATAGAAATAGGTGGTTCAGAGCAGAATACTACTAATAATCGAATGGA
ACTCA-CTGCAGCAATAAAAACTCTCGAAAAGTTAAAAAAT--TA--T-----CAA--T-
-TAAAAGAAA-ACTTTAAATTAAGAACAGATAGTAAATATGTTATTGAAGGTTATACAAA
---A---TGGATCAGTAATTGGAAGAAAAATGGATGGAAAACGAGTGCAGGAAAATCTGT
ACAGAACTTAGATTTATGGCAAAAAATAGATGAATT-A----A-G-A-ATACA-------
---------------AGGCTTAGAGATGGAATTCGTAAAAGGTCATAGCGGGATA-AAC
AGAATGA--GAGAGTTGATTTAATTG---CCACAAGCTAT---A-G-TAAGGG-------
-----------AA---T------T----CC---A----C---T----------AGATAGT
GAAGT-T-----AAAA---A--ATTA---------------T--CTT------CAGAAA-
----------------TTGATT-------------C------------------------
---------------AAAA--GAAAC---C-G-G--ATCTTCG
```

Supplementary Alignment 3

Aligned homologs of the *Trichodesmium erythraeum* twintron host gene *Tery_4732*. These alignments were were done with ProbCons (see Methods) and used to build the Bayesian tree shown in Figure 2B.

```
>Trichodesmium_erythraeum_IMS101
ATG--A--A-TGA-------A---------------ACAACTAAC---------------
GA---A-------TTA---------------GTC---------GAA-AATA--TGCCTAG
GGAGCAGAGGGTT--GGGCAACTGCGTAACTTAATTGAAACCCTACATATAGCCGATGAA
GTGGCCAATAAAGGCTATCTAATCACTAGCTCTGAACTGGCAGATTTAATGGATATCAAT
GCCAGTGCAGTTACTAGTCGTGGAGATCATTGGGTATGGCGGAACTGGGTTGTATCTAGA
GTCCGTCGTGAAGGGAACCAAAGT-----GCGATTCGTTT--------------------
--------C---------------------------------------------------
---------------------------T--------------------------------
-------GCTAA
>Cyanothece_sp_PCC_7425
ATG--T--C-TGA-------GTTGCAAGAACTGATCGCTACCGTC---------------
AC---G-------ACC---------------GATTCC---GAGGAT-CACC--TCAGTCG
CACTCTCCGGGTC--GGACAGTTGAAGAATTTAATTGAAACCCTGCGGATTGCGGAGGAA
ATTTCTAAACAGCGCTATTTAATTAGTAGTTCCGAACTGGCAGATCTGATGGATGTCAAT
GCCAGCGCCGTAACCAGTCGGGGGGATGAATGGGTCTGGCGGAACTGGGTTGTTTCCAGG
GTGCGGCGGGAAGGGAACCAGATTCTCTGGCAATTGGAACGGGT----------------
--------G---------------------------------------------------
---------------------------G--------------------------------
-------ATTAG
>Cyanobacterium_sp_UCYN-A2
ATG--A--T-TGA-------C---------------GCAACTGTC---------------
AA---T-------ACA---------------ATTGAA---GAATTT-GATC--TTTCTCG
TGAAGAGAGAGTC--ATTGAAATTCAAAACCTCATTGAAACCTTGCGTATTGCTGATGAA
ATAGCTGAGAAAGGCTATCTAATTACCAGTTCTGAATTAGCTGATTTAATGGATATTAAT
GCCAGTGCTGTTACAAGTCGTGGCGATCACTGGTCATGGCGAAACTGGGTAGTTTCTAGA
GTTCGTAGAGAAGGAAACCAAATTCTCTGGCAACTAGAAAAGAT----------------
--------T---------------------------------------------------
---------------------------G--------------------------------
-------ATTAA
>Synechocystis_sp_PCC_6714
ATG--G--A-AAA-------T---------------ATTATCAAT---------------
AA---T-------GTG---------------GAA------GAGTAT-CTGT--TATCGAG
GGAAGAACGGATC--CTCCAGGTGCGTAATTTAGTGGAAACCCTACGGGCCGCTGAAGAA
ATTGCCAAGCAGGGCTACTTAATTACCAGTTCAGAGTTGGCAGATTTAATGGATATTAAC
CCCAGTGCTGTCACTAGCCGGGGAGATCATTGGTCTTGGCGCAATTGGGTGGTGTCTCGG
GTGAGAAGGGAGGGGAACCAAATTCTCTGGCAGTTGGAGCGGGT----------------
--------G---------------------------------------------------
---------------------------G--------------------------------
-------ATTAG
>Lyngbya_aestuarii_BL_J
------------------------------------------------------------
------------------------------------------------------------
------------------------------------------TTGCATATTGCTGATGAA
ATTGCAAGTAAGGGTTACTTGATCAGTAGTTCGGAATTGGCTGATCTGATGGATGTCAAT
GCTAGTGCTGTCACCAGTCGCGGTGATAACTGGGCTTGGCGGAATTGGGAAGTTTCCAGA
```

GTTCGCCGTGAGGGAAATCAAATCCTTTGGCAATTGGAACGGGT----------------
--------A---
---------------------------G--------------------------------
-------ACTAA
>Synechococcus_sp_NKBG15041c
ATG--A--C-TGA-------A---------------ACCCAGAGT---------------
TT---T-------GTTT---------CT---GACGAT---GCCACCA-CCG--ATTCTCA
CCATGAGCGGGTG--CAACGGTTAACCGATGTCATCGAAAGCCTCCGCATTGCCGACGAA
ATTGCAGAAAATGGCTATTTCATCAGTAGTTCTGAGCTGGCAGATTTGATGGATGTTAAC
GCCAGTGCTGTCACCAGTCGCGGCCATGAGTGGACATGGCGCAATTGGATTGTTTCCCGT
GTGAAACGTGAAGGCAATCAAATTCTCTGGCAACTAGACCGCAT----------------
--------CATTG---------------------------GTGGTGA-------------
--------------TG---AGACGACGG--------------------------------
-------CCTAA
>Cyanobacterium_UCYN-A
ATG--A--T-TGA-------C---------------GCAGCTGTC---------------
AA---T-------ACA---------------ATTGAA---GAATTT-GATC--TTTCTCG
TGAAGAGAGAGTT--ATTGAAATTCAAAATCTCATTGAAACCTTGCGTATTGCTGATGAA
ATAGCTGAAAAAGGCTATTTAATTACTAGTTCTGAATTAGCCGATTTAATGGATATTAAT
GCTAGTGCTGTTACGAGTCGTGGTGATCATTGGTCATGGCGAAACTGGGTAGTTTCTAGA
GTTCGTAGAGAAGGAAACCAAATTCTTTGGCAACTAGAGAAGAT----------------
--------T---
---------------------------G--------------------------------
-------ATTAA
>Synechocystis_sp_PCC_6803
ATG--G--A-AAA-------T---------------ATTGTCAAT---------------
AA---T-------GTG---------------GAA------GAGTAT-CTGT--TATCGAG
GGAAGAACGGATT--CTCCAGGTGCGTAACTTGGTAGAAACCCTCCGGGCCGCCGAAGAA
ATTGCCAAGCAGGGGTATTTAATTACCAGTTCGGAGTTGGCTGACTTGATGGATATTAAC
CCCAGTGCCGTCACCAGTCGGGGGGATCATTGGTCATGGCGTAACTGGGTCGTTTCCAGG
GTGAGAAGGGAAGGGAACCAAATCCTCTGGCAGTTGGAGCGGGT----------------
--------A---
---------------------------G--------------------------------
-------ACTAA
>Thermosynechococcus_elongatus_BP-1
ATG--G--T-GCA-------A---------------CCATCCTTGGGGCTTGATGGTGAC
GG---TGAATGGTTGG---------------GGG------GAA----AATC--ACCCCAG
TGGTCTGAGCGCCGTGCCCGCCTTGAAGAACTCATTGTGACACTGCGAATTGCCGATGAA
TTGGCGGCTGGTAAATACTTGATTACCAGTGGCGAATTGGCAGAATTGATGGATGTCCAC
GCCAGTGCCGTCACTAGTCGCGGTGAAGAATGGGTATGGCGCAACTGGCAGATCTCGCGG
GTACGCCGTGAGAGTAACCAAATTCTCTGGCAATTGGAACGCAT----------------
--------T---
---------------------------GATGTGGC---CAAAGAGCAGTCCTCAGAATTA
CCCCCGACGTAG
>Leptolyngbya_sp_PCC_7104
ATG--G--C-AGA-------C---------------TCTGGAACT---------------
TCTCCC-------ATT---------------AGCGAAAACTTTGAT-TCTT--TACCCCG
AGAGGTCAGGGTT--GATAGCCTCCGCAACGTGCTCGAAACCCTGCAAATTGCCGACGAA
ATTGCCAAACAGGGCTATTTAATCACCAGTTCAGAACTGGCCGATTTAATGGATGTGAAC
GCCAGTGCTGTCACCAGCCGAGGAGAATTTTGGGCCTGGCGAAACTGGTCGGTGTCACGG
GTGCGGCGAGAAGGGAACCAAATTCTTTGGCAAATTGAGCGCAT----------------
--------C---
---------------------------G--------------------------------
-------ATTAG
>Lyngbya_sp_CCY_8106

```
ATG--A--A-CGA-------C---------------ATAACGAAT---------------
GA---T-------GCA---------------CAA------GAA----GTGT--TACCCCG
TGAGGTTAGGGTT--AACCAACTTCGTAATTTGATAGAAACCTTGCATATTGCTGATGAA
ATTGCCAGCAAGGGTTACTTGATCAGTAGTTCTGAATTGGCTGATCTGATGGATGTCAAT
GCTAGTGCTGTCACCAGTCGCGGTGATAACTGGGCTTGGCGGAATTGGGAAGTCTCTAGA
GTTCGCCGTGAGGGAAATCAAATCCTTTGGCAATTGGAACGGGT----------------
--------A---------------------------------------------------
---------------------------G--------------------------------
-------ACTAA
>Cyanothece_sp_CCY_0110
ATG--A--T-TGA-------C---------------TCATCTGTC---------------
AA---T-------ACA---------------ATTGAA---GAATTT-GATC--TCTCCCG
TGAAGAGAGAGTC--ATCGAAATTCAAAACCTCATTGAAACCCTACGCATTGCTGATGAA
ATCGCTGATAAGGGTTACTTAATCACTAGCTCAGAATTAGCGGATTTAATGGATATAAAT
GCCAGTGCTGTCACCAGTCGGGGTGATCATTGGTCTTGGCGTAACTGGGTTGTTTCACGG
GTTCGCCGAGAAGGAAATCAAATTCTTTGGCAACTCGAAAAAAT----------------
--------T---------------------------------------------------
---------------------------G--------------------------------
-------ATTAA
>Arthrospira_sp_PCC_8005
ATG--A--C-TGA-------G---------------CCAATCAAC---------------
GA---G-------TCC---------------AAT---------GAG-AGTT--TACCCCG
GGAATTTCGAGTC--AATCAACTTAGGAATCTCATAGAAACCCTGCATATTGCCGACGAA
ATTGCTAGTAAAGGCTATTTGATCAGTAGTTCAGAACTTGCTGATTTAATGGATATTAAC
GCTAGTGCGGTCACCAGTCGCGGCGATAACTGGGCGTGGCGAAATTGGGAAGTTTCCCGG
GTTAGACGGGAAGGCAATCAAATCCTCTGGCAATTGGAGAGGGT----------------
--------T---------------------------------------------------
---------------------------G--------------------------------
-------ATTAG
>Planktothrix_NIVA-CYA_98
ATG--A--C-TGA-------C---------------TTAATTAAT---------------
GA---T-------TCA---------------CTA---------GAA-AACC--TACCGCG
CGAGGTTAGGGTG--AACCAACTTCGCAATTTGATCGAGACCCTGCATATTGCTGATGAA
ATTGCTACCAAGGGCTACTTAATCAGTAGTTCTGAACTGGCCGATCTCATGGATATTAAT
GCCAGTGCTGTCACCAGTCGAGGGGATAACTGGGCATGGCGCAATTGGGAAGTATCCAGA
GTGCGGCGAGAAGGTAATCAAATTCTCTGGCAATTAGAACGAGT----------------
--------T---------------------------------------------------
---------------------------A--------------------------------
-------ACTAG
>Oscillatoria_acuminata_PCC_6304
ATG--G--A-CAC-------A---------------ACTGCCCATTCCACTA------CT
GA---A-------TCC---------------CTT---------GAC-AACT--TACCCCG
TGAGGTGAGGGTC--ACTCAACTCCAGTACCTGATGCAAACCCTGGAAATCGCCGATCGC
ATCGCCACCAAAGGCTATCTGATCACGAGTTCCGAACTAGCCGACCTGATGGATGTCAAT
GCCAGCGCCGTCACCAGTCGCGGCGACAACTGGCCTTGGCGTAACTGGATCGTCTCCCGG
GTGCGACGAGAAGGAAACCAAATTCTCTGGCAACTAGAACGAAT----------------
--------T---------------------------------------------------
---------------------------GATTTATTTAACGGATCCGGTGAGGATGATGGG
GCCGGAGAATAA
>Crocosphaera_watsonii_WH_8501
ATG--A--T-TGA-------C---------------TCATCTGTC---------------
AA---T-------ACA---------------ATTGAAGAATTC----GATC--TCTCCCG
TGAAGAGAGAGTC--ATTGAAATTCAAAACCTCATTGAAACCCTACGGGTTGCTGAGGAG
ATCGCTAGTAAAGGTTACTTAATTACTAGCTCAGAATTAGCTGATTTAATGGACATCAAT
GCCAGTGCAGTCACCAGTCGTGGTGATCATTGGTCTTGGCGTAACTGGGTTGTTTCGAGG
```

GTTCGCCGAGAAGGAAACCAAATTCTTTGGCAACTGGAAAAAAT----------------
--------C---
---------------------------G--------------------------------
-------ATTAA
>Acaryochloris_marina_MBIC11017
CTC--C--C-GAAGTGAATCA---------------ACATCCT---C-----------CC
GA---T-------CAT---------------GCA------GAA----CATC--CTGCCAG
TGAATCGAGGATC--TCTCATCTGCAGGATTTAGTCGAAACGTTGGAAATTGCCGATCAC
ATTGCGAAAGAGGGTTATCTAATCAGCAGTTCTGAATTAGCAGATCTAATGGCGGTCCAC
GCCAACGCGGTCACTAGCAGAGGGGATGAATGGGTGTGGCGCAATTGGCTAATCTCCCGT
GTCCGTCGGGAAGGCAACCAGATTCTGTGGCAGTTAGAGCGTCT----------------
--------T---
---------------------------AAC------------------------------
------TCCTAG
>Synechococcus_sp_PCC_7002
ATG--A--C-AGA-------T---------------CTCACCAGC---------------
CT---T-------G------------CC---CCTGAA---GACATTA-CCC--TGCCCTA
CCATGAACGAGTT--AAGCGCTTAACAGACCTCATCGAAAGTCTCCGTATTGCCGATGAA
ATTGCGGAAAATGGTTATTTTATTAGTAGTTCTGAGTTGGCAGATCTAATGGATGTGAAT
GCCAGTGCCGTCACCAGTCGGGGCCATGAATGGACTTGGCGCAATTGGATTGTCTCCCGG
GTCAAACGAGAAGGTAATCAGATCCTTTGGCAACTCGACCGCAT----------------
--------TATC------------------------------GGTGA-------------
--------------TG---AGACGACGA--------------------------------
-------CCTAA
>Cylindrospermopsis_raciborskii_CS-505
ACT--A--T-GGA-------A---------------ACATCTACA---------------
GG---A-------ATAT---------CT---GTGGTGACACAGGAA-GAAA--AACCCCC
GGAAATGAGAATA--TTACAAATCCGTAATCTCATGGAAACCCTAAAGATTGCGGACGAA
ATTGCCCAGAAAGGTTACTTAATCACCAGTTCCGAACTAGCAGATTTAATGGATGTTCAT
GCCAGTGCAGTGACAAGTCGCGGTGATCAGTGGAGATGGCGTAACTGGATAGTGTCACGA
GTCAGACGAGAAGGGAATCAGATTTTGTGGGAGCTGGAAAGGGG----------------
--------CGATC---------------------------AAGTGGA-------------
--------------AA---AGGATGATA--------------------------------
-------ATTAA
>Raphidiopsis_brookii_D9
ACT--A--T-GGA-------A---------------CCATCTACA---------------
GG---A-------ATAACAGGAATATCT---GTGGTGACACAGGAA-GAAA--AACCCCC
GGAAATGAGAATC--TTACAAATCCGTAATCTCATGGAAACCCTAAAGATTGCAGACGAA
ATTGCCCAGAAAGGTTACTTAATCACCAGTTCTGAACTAGCAGATTTAATGGATGTTCAT
GCCAGTGCCGTGACAAGTCGCGGTGATCAATGGCGATGGCGAAACTGGATAGTGTCACGG
GTTAGACGTGAGGGGAATCAGATTTTGTGGGAGCTGGAAAGGGG----------------
--------CGATC---------------------------AAGTGGA-------------
--------------AA---AGGATGATA--------------------------------
-------ATTAA
>Halothece_sp_PCC_7418
ATATGC--AAGAACTAGAGGA---------------ACGGACA-----ATC---------
GA---G-------GAT---------------TCT-------------CGCCTGGTGCAAG
--GCGATCGGATG--GAAGGACTGAATCATTTAATTGAATCCCTCAGAATTGCCGATGAA
ATTGCCGAAAAAGGCTATCTCATTAGTAGTTCTGAACTTGCGGATTTAATGAATGTTAAC
GCCAGCGCCGTTACCAGTAGAGGCAATCACTGGTCTTGGCGCAATTGGGTCGTTTCACGA
GTTCGACGGGAGGGAAACCAAATTCTCTGGCAACTAGAACGAGC----------------
--------A---
---------------------------GAA------------------------------
------GGCTAA
>Leptolyngbya_sp_PCC_7376

```
ATG--A--C-TGA-------C---------------TCCGCTACA---------------
AA---C-------T------------CT---ACAGAAGAACTTG----TTT--TATCCCG
TCAAGAGCGCGTT--CACCAGCTATCTGCCTTAATTGAGAGCCTCCGTATCGCCGATGAA
ATCGCAGAGAAAGGCTACTTTATCAGTAGCTCCGAACTTGCTGATCTCATGGATGTAAAT
GCCAGTGCTGTAACTAGCCGTGGCCACGAATGGACATGGCGTAATTGGATCGTTTCCCGT
GTGAAGCGTGAAGGTAACCAAATTTTGTGGCAACTAGACCGCAT----------------
--------TATG------------------------------GGCGA-------------
--------------TG---CCGAGGCTT--------------------------------
-------CCTAA
>Calothrix_sp_PCC_7103
ATG--A--T-AGA-------T---------------GCTTCTATCTCCTATGCGAGCAAC
AA---T-------ACT---------------AAT---------GAA-GAGA--TACCACG
CGAAATGCGGGTA--ACGCAACTTCGCAACCTCGTTGATACCCTCCGTATCGCCGATGAA
ATTGCTGATAAAGGTTATTTAATAACCAGTTCTGAACTAGCTGATTTAATGGATGTTCAT
GCCAGCGCTGTTACCAGTCGTGGAGATCAGTGGCGCTGGCGAAATTGGGTTGTCTCTCGC
GTTCGTAGAGAAGGTAATCAAATACTTTGGGAACTCGAACGTGGAGATAAAATTCAGGAA
GAGAGCAAT---------------------------------------------------
---------------------------G--------------------------------
-------AGTAA
>Microcoleus_sp_PCC_7113
ATG--A--T-GGA-------T---------------TCACCTGTT---------------
TC---T-------TCCC---------CTGCTGGTGCA---ATTGAA-GACT--TACCTCG
CGAGGTGAGAGTT--GCTCAACTCCGTAACTTGGTGGAAACTCTCCACATCGCCGATGAG
ATAGCCCGCCAAGGCTACCTGATTAGCAGTTCTGAGCTGGCTGACTTAATGGATGTTAAT
GCCAGTGCCGTGACTAGTCGCGGCGACCACTGGCCTTGGCGAAATTGGATTGTGTCACGA
GTCCGACGTGAAGGCAATCAAATTCTTTGGCAACTTGAACGAGT----------------
--------GGATC---------------------------AAGTGAATACAG--------
-------------CAG---ATGATGAAG--------------------------------
-------GTTAA
>Rivularia_sp_PCC_7116
ACT--A--T-GGA-------A---------------TCATCTAGC---------------
GC---T-------TCTG---------CTACCTTGCAAAACGAA----GATT--TACCACG
CGAAACAAGAGTC--TCGCAACTCCGCAGTTTAGTTGAAACTCTTCGCATTGCTGACGAA
ATAGCCGAAAAAGGTTACTTAATCACAAGTTCCGAATTAGCGGACTTAATGAACGTCCAT
GCTAGTGCTGTTACTAGCCGTGGAGACCAATGGCGTTGGAGAAACTGGATAGTTTCCCGA
GTACGTCGCGAGGGTAATCAAATACTTTGGGAACTCGAACGCGC----------------
--------CGATA---------------------------AACTTGAC---G--------
-------------ATG---ATGAAGGAG--------------------------------
-------AGTAA
>Acaryochloris_sp_CCMEE_5410
CTC--C--C-GAAGTGAATCA---------------ACATCCT---C-----------CC
GA---T-------CAT---------------GCA---------GAA-CATC--CTGCCAG
TGAATCAAGGATC--TCTCATCTGCAGGATTTAGTCGAAACGTTGGAAATTGCCGATCAC
ATTGCGAAAGAGGGTTATCTAATCAGCAGTTCTGAATTAGCAGATCTAATGGCGGTCCAC
GCCAACGCGGTCACTAGCAGAGGGGATGAATGGGTGTGGCGCAATTGGCTAATCTCCCGT
GTCCGTCGGGAAGGCAACCAGATTTTGTGGCAGTTAGAGCGTCT----------------
--------T---------------------------------------------------
---------------------------AAC------------------------------
------TCCTAA
>Synechocystis_sp_PCC_7509
AT---A--T-GGA-------T---------------TCACCAGCC---------------
CC---A-------ATAA---------ATACTCATGC------AGAC-GAAT--TACCCCG
CGAGGTCAAAGTG--GGACAGTTGCGCCACTTGATTGATACGCTGCGTATAGCGGAAGAA
ATTGCGACCAAAGGCTACCTAATTACAAGTTCCGAACTTGCCGATTTGATGGATGTCCAC
GCCAGCGCCGTCACCAGTCGCGGAGACGAGTGGCGCTGGCGCAACTGGATTGTGTCGAGA
```

```
GTAAGACGAGAAGGCAATCAAATTCTGTGGGAATTAGAACGAGG----------------
--------AGACG---------------------------ATGTTAGT---TCGGCAACT
AGCTATAACTCCGACC---CCGCCACTG--------------------------------
-------AGTAG
>Richelia_intracellularis_HH01
ATG--A--T-TGA-------C---------------TCATCCAAT---------------
GC---C-------ACTG---------CTTCACTAAAT---AGTGAG-ACGA--TTTCTAG
TGAATTAAGGATG--GTGCACATCCGTAACCTATTGGAAACCCTTCATGTCGCTGACGAA
ATTGCAGCTAAAGGTTATTTGATAACCAGTTCCGAACTGGCAGACTTAATGGGTGTTCAT
GCCAGTGCTGTTACTAGTAGGGGAGATGAATGGCGCTGGCGTAACTGGGTAGTTTCACGT
GTTAGAAGAGAAGGTAATCAAATTCTTTGGGAACTAGAGCGGGG----------------
--------TGATC---------------------------TATTTGAT---G--------
-------------AGG---AAGATGAAA--------------------------------
-------ATTAA
>Nodularia_spumigena_CCY9414
GCT--A--T-GGA-------A---------------CCATCCAGC---------------
GC---A-------CAAG---------CT---GCGGCCGCAAATGAA-GAAA--AACTGTC
TACAACCAGGGTA--AAGCAACTCAAAAACTTGCTGGAGACTCTGCGTATAGCTGAGGAA
ATTTCGGAAAAAGGCTACTTAATCACCAGTTCTGAACTAGCAGACTTGATGGATGTCCAC
GCCAGTGCTGTAACTAGTCGGGGAGACGAGTGGCGCTGGCGGAACTGGATAGTGTCACGG
GTAAGACGCGAAGGAAATCAAATTCTCTGGGAACTGGAACGAGG----------------
--------CGATTGCGGTTCGCGCAGTGCCGGAGGCGATCGCCTAGA-------------
--------------AACCGAAGACGAAA--------------------------------
-------TATGA
>Nostoc_sp_PCC_7120
ACT--A--T-GGA-------A---------------CCATCTACC---------------
GC---A-------CACG---------TT---GCGGCTACAATCGAG-GAAA--ACCCGCC
GGAAAAGCGGGTG--GAACAACTCCGCAACTTAGTAGAAACTCTGCATATTGCAGACGAA
ATTGCGGCGAAAGGTTATTTAATCACTAGTTCTGAGTTGGCAGACTTAATGGATGTCCAC
GCCAGTGCTGTTACTAGTAGGGGAGATCAGTGGCGCTGGCGCAACTGGATTGTTTCACGG
GTACGCCGTGAGGGTAATCAAATTCTCTGGGAATTAGAACGAGG----------------
--------CGATC---------------------------GCTTGGG-------------
--------------GG---GTGAAGATG--------------------------------
-------AATAA
>Synechococcus_elongatus_PCC_6301
GTT--CGGA-TGC-------C---------------TGAAGTTGT---------------
GA---C-------CCA---------------AGAGTG---G-----AGCAG--TAACCCT
CGCGATCGCAGT---CAACGCCTGCGCGATGTGCTGGACTGCTTGCAAATTGCTGCAGCA
ATTGCTGAGCAGCAATTTTTGATCACGAGTTCAGAGCTGGCTGACCTGATGGATGTCAAC
GCCAGTGCGGTCACCAGTCGGGGCGAAGAGTGGATTTGGCGCAACTGGCGGGTGAAGCGC
GATCGCCGTGAAGGCAATCAAATCCTCTGGCAGCTCGAGCGCAT----------------
--------T---------------------------------------------------
---------------------------C--------------------------------
-------ATTGA
```

Supplementary Alignment 4 Alignment of the *T. erythraeum* IMS101 twintron and its host gene exons with the respective sequences from metagenomic datasets. TrichoMGDRAFT_c100672 originates from Hawaii (Pacific Ocean), the TCCM contigs originate from Bermuda (Atlantic Ocean). Identical residues in all three sequences are represented by a dot. The black box and the sequence below it show the consensus of the alignment. The transcription start site (TSS) of the pre-transcript in *T. erythraeum* IMS101 is indicated by an arrow, the mRNA exons (green) and intron IEP ORFs (red and blue for the inner and host intron, respectively) are boxed. The starts of the host intron (blue) and the inner intron (red) are indicated by open boxes. Note that interestingly, the intron sequence is generally better conserved than the two IEP ORF sequences.

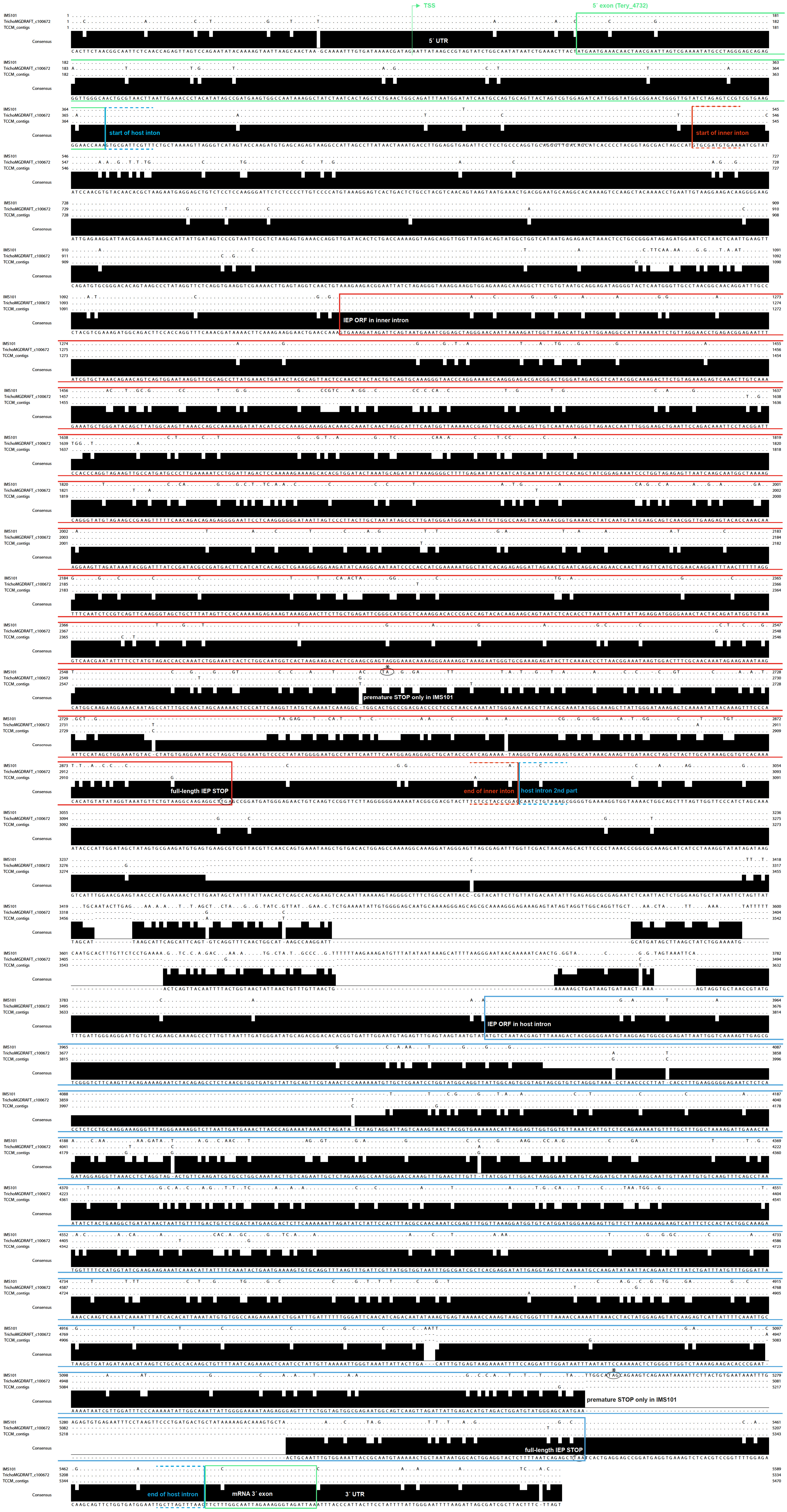

Supplement: Supplementary Materials [file srep16829-s1.pdf]
